# Genomic and Transcriptomic Analysis Identified Novel Putative Cassava lncRNAs Involved in Cold and Drought Stress

**DOI:** 10.3390/genes11040366

**Published:** 2020-03-28

**Authors:** Rungaroon Suksamran, Treenut Saithong, Chinae Thammarongtham, Saowalak Kalapanulak

**Affiliations:** 1 Biotechnology Program, School of Bioresources and Technology, King Mongkut’s University of Technology Thonburi (Bang KhunThian), Bangkok 10150, Thailand; rungaroon.poy@mail.kmutt.ac.th; 2 Bioinformatics and Systems Biology Program, School of Bioresources and Technology, King Mongkut’s University of Technology Thonburi (Bang KhunThian), Bangkok 10150, Thailand; treenut.sai@kmutt.ac.th; 3 Center for Agricultural Systems Biology, Systems Biology and Bioinformatics Research Group, Pilot Plant Development and Training Institute, King Mongkut’s University of Technology Thonburi (Bang KhunThian), Bangkok 10150, Thailand; 4 Biochemical Engineering and Systems Biology Research Group, National Center for Genetic Engineering and Biotechnology at King Mongkut’s University of Technology Thonburi (Bang KhunThian), Bangkok 10150, Thailand

**Keywords:** lncRNA, cassava, comparative approach, cold and drought stress

## Abstract

Long non-coding RNAs (lncRNAs) play important roles in the regulation of complex cellular processes, including transcriptional and post-transcriptional regulation of gene expression relevant for development and stress response, among others. Compared to other important crops, there is limited knowledge of cassava lncRNAs and their roles in abiotic stress adaptation. In this study, we performed a genome-wide study of ncRNAs in cassava, integrating genomics- and transcriptomics-based approaches. In total, 56,840 putative ncRNAs were identified, and approximately half the number were verified using expression data or previously known ncRNAs. Among these were 2229 potential novel lncRNA transcripts with unmatched sequences, 250 of which were differentially expressed in cold or drought conditions, relative to controls. We showed that lncRNAs might be involved in post-transcriptional regulation of stress-induced transcription factors (TFs) such as zinc-finger, WRKY, and nuclear factor Y gene families. These findings deepened our knowledge of cassava lncRNAs and shed light on their stress-responsive roles.

## 1. Introduction

Non-coding RNAs (ncRNAs), once dubbed as ‘junk’ sequences in genomes, have been shown to regulate diverse cellular processes [1]. These RNA molecules are quite abundant in genomes, approximately 90%, as only a small fraction of the transcribed molecules in the genome are translated [2]. In mammals, ncRNAs are thought to span over 70 percent of the whole genomic regions [3], and a higher percentage is expected in plants. Non-coding RNAs are quite diverse. Based on size, they can be broadly categorized into short and long ncRNAs, with the latter being over 200 nucleotides long [1]. In relation to their functional relevance, housekeeping ncRNAs, e.g., transfer RNAs (tRNAs), ribosomal RNAs (rRNAs), small nuclear RNAs (snRNAs), small nucleolar RNAs (snoRNAs), and RNase P RNAs, are constitutively expressed and are involved in basic cellular processes, whereas regulatory ncRNAs, e.g., small interference RNAs (siRNAs), microRNAs (miRNAs), and long non-coding RNAs (lncRNAs), are timely expressed in particular conditions [4]. Some studies have reported the involvement of ncRNAs in a range of regulatory processes in animals, humans, and plants. The let-7 microRNA precursor containing a small RNA complementary to 3’UTR of the heterochronic switch genes lin-14 and lin-28, regulates cell fate transformation and developmental timing in *Caenorhabditis elegans* [5]. Xist lncRNA was found to play a role in transcriptional silencing of the X-chromosome in mammals by directly interacting with SMRT/HDAC1 Associated Repressor Protein (SHARP), which is essential for both silencing and exclusion of RNA polymerase II from the inactive X-chromosome during development [6]. In Arabidopsis, the lncRNA COLD ASSISTED INTRONIC NONCODING RNA (COLDAIR) was reported to be involved in epigenetic silencing of the floral repressor gene, *Flowering Locus C* (*FLC*), during vernalization to enable flowering [7].

Long non-coding RNAs are among the most studied ncRNAs in plants, in addition to microRNAs. They are reported to be structurally conserved but subtle in primary sequences and low levels of expression [8]. Generally, lncRNAs are classified, based on their locations, as intergenic (lincRNA) and intronic lncRNAs [9]. Besides, *cis*-natural antisense lncRNAs (*cis*-NATs or lncNATs), which are transcribed from the same genomic loci as their targets but from the complementary strands [10], and *trans*-NATs, which are transcribed from different locations as their targets and allow multiple transcript complementarity with few mismatches, are other important groups of lncRNAs. Research has explored lncRNAs in plants to gain insights into their regulatory functions, particularly in relation to development and stress response. In Arabidopsis, Alternative Splicing Competitor long noncoding RNA (ASCO-lncRNA) mediates alternative splicing patterns during lateral root development, and its overexpression hinders lateral root formation [11]. Zhang et al. [12] showed that the XLOC_057724 lncRNA played a role in rice panicle development and fertility. Its inactivation resulted in a decrease in spikelet fertility [12]. Regulatory lncRNAs are also reported to regulate stress response mechanisms in plants. Overexpression of *DRIR* lncRNA enhanced drought and salt tolerance in Arabidopsis via modulating downstream stress-responsive genes [13]. Silencing of GhlncNAT-ANX2 and GhlncNAT-RLP7 enhanced fungal (*Verticillium dahlia* and *Botrytis cinerea*) resistance in cotton [14]. 

Despite being an important staple crop, knowledge of lncRNAs in cassava is very limited, with only a few reported studies [15,16,17,18,19]. 682 lincRNAs, including lncNATs linked to cold or drought response [15] and 15 lncNATs responsive to *Xanthomonas axonopodis* pv. *manihotis* [20], have been reported. Recently, Ding and colleagues suggested the potential role of lncRNAs involved in hormone metabolism under simulated drought stress [16] and melatonin treatment [17]. Wu et al. suggested that four lncRNAs contained the binding sites of miR156 and miR159, which were functionally well-known as miRNAs involved in ABA- and drought-response [18]. Xiao et al. suggested that two lncRNAs mediated drought tolerance by regulating stomatal density in autotetraploid cassava via co-expressed target genes encoding for subtilisin-like proteases [18]. These reports are valuable and beneficial for more understanding in functional lncRNAs under drought stress in cassava. However, these were identified via a transcriptomics approach, which limits the identification scope to transcripts expressed in the studied conditions. 

**Figure 1:**
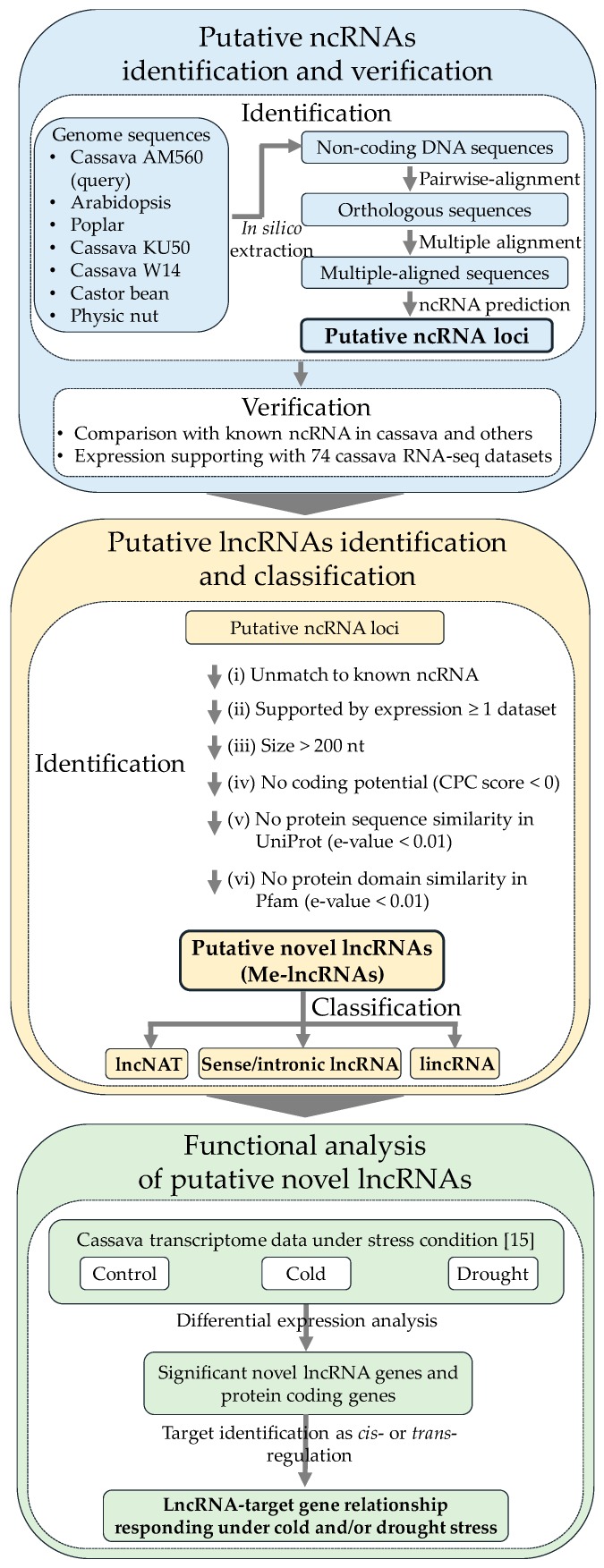
Overview of workflow for putative non-coding RNAs’ (ncRNAs) identification and verification, putative long non-coding RNAs’ (lncRNAs) identification and classification, and functional analysis of putative novel lncRNAs. LncNAT: long non-coding natural antisense transcript. Sense/intronic lncRNA: Sense/intronic long non-coding RNA. LincRNA: long intergenic non-coding RNA. CPC score: Coding potential calculation score.

Genome sequence-based and computational approaches have been used for genome-wide prediction of putative ncRNAs based upon the homology of RNA sequence and structure. The genome sequence-based approach employs sophisticated features, including probabilistic models, thermodynamic stability, and structural covariation analysis, to ensure robust ncRNA prediction [21] and has been applied in a wide range of species, including bacteria [22,23], insects [24], humans [25], and plants [26]. In this study, we integrated the genomics- and transcriptomics-based approaches to identify putative ncRNAs at a genome-wide scale. We discovered 56,840 putative ncRNAs of cassava, about half of which were verified expression by transcriptome data. Comparisons with known ncRNAs revealed a partial overlap between the transcripts and a distinct set of lncRNAs. The newly identified lncRNAs were further investigated to ascertain their regulatory functions in cold and drought conditions, resulting in a set of promising abiotic stress-responsive lncRNAs, along with their targets. An overview of the workflow is shown in Figure 1.

## 2. Materials and Methods 

### 2.1. Genomes and Annotations

The genome sequence of cassava cultivar AM560 (v6) and the annotation were retrieved from the Phytozome12 database [27]. Likewise, genome sequences of related plant species used in this study: Arabidopsis (TAIR10), Poplar (v3) and Castor bean (v0.1), along with their annotation, were obtained from the same source. The genome sequence of Jatropha was retrieved from the Jatropha genome database [28], and sequences of cassava cultivars KU50 and W14 were obtained from the Chinese cassava database [29].

### 2.2. Identification of ncRNA Loci Based on Comparative Approach

Non-coding sequences in the genic (intron and untranslated regions) and intergenic regions of cassava and the other six plant species were extracted from their genomic sequences. To prepare query sequences for RNAz, the non-coding sequences of cassava cultivar AM560 were aligned with those of the six plant species, firstly through pairwise alignment using BLASTn (e-value < 0.01) then the resulting best hits for each pairwise alignment were combined through multiple sequence alignment using MUSCLE [30]. Only sequences that were ≥50 bps in length and were conserved in at least three plant species, including cassava AM560, were selected for ncRNA prediction using RNAz [31]. Oversized query sequences (>400 nt), were cut into 120 nt windows with 80 nt sequence overlap. The probability of each 120 nt window containing structural ncRNAs was predicted. The query windows with a probability (*P*) value > 0.5 were designated as positive windows. The overlapping adjacent positive windows were merged and designated as predicted ncRNA loci. Then, the highest *P* value of the merged windows was used to represent the confidence of the predicted ncRNA loci. Putative ncRNA loci with *P* > 0.9 were classified as high-confident predicted ncRNA loci.

### 2.3. Verification of Predicted ncRNA Loci

#### 2.3.1. Comparison with Known ncRNAs based on Sequence or Structural Similarity

Previously reported cassava ncRNAs from RNAcentral release 13 (https://rnacentral.org/), NCBI release 100 (https://www.ncbi.nlm.nih.gov/), GreeNC release 1.12 (http://greenc.sciencedesigners.com/), CANTATAdb release 2 (http://cantata.amu.edu.pl/), miRbase release 22 (http://www.mirbase.org/), and seven publications [15,20,32,33,34,35,36] were compared with our predicted ncRNA loci by sequence similarity. The sequence similarity analysis was performed via detecting the mapped positions of sequences on the cassava AM560v6 genome using BEDTools [37]. For structural similarity, covariance models and Hidden Markov Models of RNA families of both prokaryotes and eukaryotes, from Rfam (https://rfam.xfam.org/) [38], were compared with our putative ncRNA loci using the INFERNAL package (e-value < 0.01).

#### 2.3.2. Expression Support Using Cassava RNA-Sequencing Datasets

A total of 74 transcriptome-wide RNA-sequencing (RNA-seq) reads, generated from 71 polyA-tail libraries and 3 strand-specific total RNA libraries, were used as expression evidence for the predicted ncRNAs. These were retrieved from cassava SRA accessions SRP101302 [15], SRP042139 [32], SRP076160 [39], and SRP096257 [40] in NCBI and from RNA-seq datasets [41] provided by the Genomic Research Laboratory, National Center for Genetic Engineering and Biotechnology (BIOTEC), Thailand. Reads with adapter sequences or those with low quality were trimmed and/or filtered out by Trimmomatic [42]. Then, quality assessment was conducted by FastQC [43]. After preprocessing, around 3.5 billion qualified reads were aligned with the cassava reference genome (AM560v6) using the STAR aligner [44]. The abundance of reads was determined at the boundaries of putative ncRNA loci. Predicted ncRNA loci with ≥10 reads covering the entire ncRNA length in at least one dataset were considered as expression evidence. The reads were normalized using GeTMM [45] and used to quantify expression levels of the putative ncRNAs.

### 2.4. Identification of Putative lncRNA Loci

We collected putative ncRNA loci longer than 200 nt with expression support but no sequence or structural similarity to known ncRNAs. Those with no coding potential (CPC score < 0), no protein sequence similarity to UniProt database (https://www.uniprot.org/) with e-value < 0.01, and no protein domain similarity to Pfam database (https://pfam.xfam.org/) with e-value < 0.01 were kept for further analysis and lncRNA biotype classification. The genic ncRNA loci that showed no overlap with coding sequences (CDS) were identified as sense/intronic lncRNA loci. The intergenic ncRNA loci with a distance of over 500 nt to flanking protein coding genes were identified as long intergenic ncRNAs (lincRNAs). The ncRNA loci flanked by protein coding genes at a distance of >500 nt and overlapping protein coding genes on the complementary strands with expression supported by strand-specific RNA-seq datasets were determined as long non-coding natural antisense transcripts (lncNATs). 

### 2.5. Functional Analysis of Putative lncRNAs Involved in Cold or Drought Stress by RNA-seq Transcriptome Analysis

We further analyzed potential functions of the putative lncRNAs by integrating with cassava transcriptome data under cold or drought stress [15]. The normalization of reads and differential expression analyses of lncRNAs and protein-coding genes were performed using DESeq2 [46]. Protein coding genes and putative lncRNAs with an absolute log2 fold change ≥ 2 and q-value ≤ 0.05, between the stress and control conditions, were considered significantly differentially expressed and potentially related to relevant stress. Then, their functions were inferred from their predicted targets. *Cis*-regulatory targets of differentially expressed lncRNAs were predicted from differentially expressed protein coding genes (in the same condition) located within 10 Kb upstream or downstream. *Trans*-regulatory targets were identified by (i) potential direct binding between the differentially expressed lncRNA sequences and corresponding mRNAs of differentially expressed protein coding gene(s) in the same condition based on sequence complementarity and optimal free energy of hybridization [47], or (ii) potential target mimicry of differentially expressed lncRNA sequences with the same miRNA binding to differentially expressed protein coding genes in the same condition, using psRNATarget with ≤2 mismatch allowed [48].

### 2.6. GO Enrichment Analysis and Visualization

Functional annotation of differentially expressed lncRNA loci was based on their target genes. GO enrichment analysis of target genes was performed using GOATool [49]. Enriched GO terms were identified by the Benjamini Hochberg’s approach at False Discovery rate (FDR) ≤ 0.05. Visualization of results was performed using ggplot2 in R [50]. Multiple alignments of RNA and protein sequences were performed using LocARNA tool [51] and T-Coffee tool [52], respectively. RNA-seq read coverage and mappings were displayed by IGV tool [53]. 

## 3. Results and Discussion

### 3.1. Putative ncRNAs’ Identification and Verification

Based on the overall workflow (Figure 1) in this study, non-coding DNA sequences were extracted from both genic (3’UTR, 5’UTR, intron) and intergenic regions of the cassava AM560 genome. Approximately two hundred thousand sequences covering approximately 97% of the genome were obtained and explored for ncRNAs identification. Using the RNAz tool, 56,840 ncRNA loci were predicted (Figure S1). These covered only 0.73% of the genome sequences in terms of the DNA base content, whereas protein-coding sequences (CDS) accounted for 3% of the genome, based on annotation from Phytozome12. This result is corroborated by the genome-wide study of ncRNAs in humans, which reported a low DNA base content (0.07%) for genes encoding putative ncRNAs [54].

In addition, we considered the number of our predicted ncRNAs (56,840) by comparing with the number of previously identified ncRNAs and lncRNAs in other organisms. The number of putative ncRNAs in this work was performed by the genome sequence-based approach, independent from investigated experimental conditions. All non-coding sequences in the genomes, based on genome sequence and annotation information, have offered all possibilities for ncRNAs’ prediction. Therefore, size, quality, and complexity of the genome are major effects for the numbers of predicted ncRNAs. When comparing to reported number of putative lncRNAs from condition-dependent approaches like transcriptome-based in Arabidopsis (≥37,238), wheat (≥58,218), barrelclover (≥23,324), maize (≥20,163) [9], human (20,000 ncRNAs) and mouse (18,000 ncRNAs) [55], it suggested that our reported number (56,840 ncRNAs) was not overestimated. Additionally, riboregulator as ncRNAs might be essential for plant adaptation and survival under stimuli or environmental perturbation in a sessile organism like plants, such as cassava. This might be the reason for a higher number of ncRNAs in cassava. 

In order to consolidate, we verified our 56,840 predicted cassava ncRNAs by using two methods, comparing with previously published ncRNAs in any organisms including cassava based on sequence and structural similarities, and by finding expression support from several available cassava transcriptome datasets. Based on our analysis, 5008 of the 56,840 putative ncRNA loci shared sequence similarity and/or structural similarity with known ncRNAs. Most of the loci matched previously reported cassava ncRNAs in RNAcentral, NCBI, GreeNC, CANTATAdb, miRBase, and in several publications, including lncRNAs involved in cold and drought stress [15], miRNAs from genome-wide screening [36], miRNAs related to the root developmental process [32], and miRNAs responsive to biotic stress [20,33] and abiotic stress [34,35]. In addition, based on the covariance model generated with Rfam data, 915 of the 5008 loci shared structural similarity to known ncRNAs in eukaryotes or prokaryotes. Rationally, these 5008 putative ncRNAs (approximately 9% of our predicted ncRNAs) were considered to be known ncRNAs.

**Figure 2:**
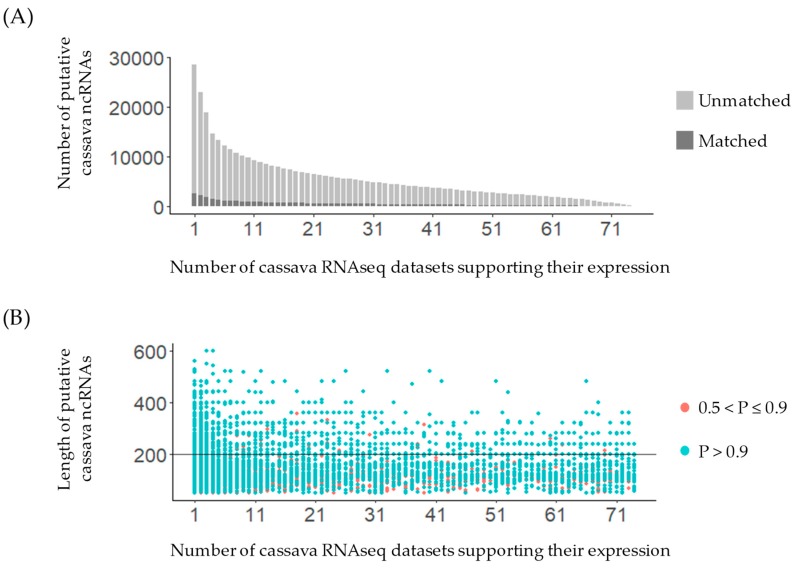
Consolidation of predicted ncRNA loci. (**A**) Inverse cumulative frequency graph of 56,840 putative ncRNA loci with number of RNA-seq datasets supporting their expression (Mapped reads ≥ 10 and coverage of breadth equal to 100%). (**B**) Characteristics of 51,832 putative ncRNA loci unmatched with previously published ncRNAs in terms of length, number of RNA-seq datasets supporting their expression, and the level of confidence based on RNAz-derived probability of being ncRNAs. The black line represents the cutoff for long and short putative ncRNAs.

Moreover, 28,633 of the 56,840 putative ncRNAs (>50% of our predicted ncRNAs) were supported by expression evidence, both in terms of the number of RNA-seq datasets and the level of expression (see more detail in the Materials and Methods Section 2.3.2). Although 91% of the predicted ncRNAs do not have a match to any other known ncRNAs, over 50% of them (28,633) do have a match to cassava RNA-seq data, at least one dataset (Figure 2A). Notably, 195 putative ncRNA loci were supported by all 74 expression datasets, which strongly suggests they truly exist in cassava given their ubiquitous expression in various tissues and conditions. In addition, these likely function as universal modulators in various conditions, or have house-keeping functions. However, further experimental validation is necessary, as in all cases. 

Furthermore, RNAz-analysis revealed that all predicted loci are structural ncRNAs. The expression of loci with evolutionarily conserved secondary structure at 0.5 probability was comparable to those predicted at 0.9 probability (Figures S2–S7). The putative ncRNAs with *P* > 0.9 had wide-ranging expression support, from 1 to the entire 74 datasets (Figure 2B). Those with low *P* (0.5 < *P* ≤ 0.9) showed a similar pattern with several RNA-seq datasets supporting their expression. More than 8% of them were supported by over 37 RNA-seq datasets. In terms of gene expression, the expression levels of both protein coding genes and putative ncRNAs were comparable (Figures S2–S7). Many putative ncRNAs showed high levels of expression, more than the 95th percentile rank, which suggests their true existence in the cassava genome. Typically, average expression levels of protein-coding genes were relatively higher than those of ncRNA genes reported in previous publications [15,56,57]. However, in this work, the expression levels of both protein coding genes and putative ncRNAs are analyzed for each individual RNA-seq dataset (Figures S2–S7). We found that the expression level of putative ncRNAs was either higher or lower than those of protein-coding genes, depending on transcriptome conditions such as cultivars, tissues, and treatment. For example, the expression level of putative ncRNAs was higher than protein-coding genes in cold or drought stress conditions (Figure S2), whereas inverted results were found in leaf and storage root tissues of cassava KU50 cultivar (Figure S3).

For the remaining 25,819 unsupported ncRNAs, they might not be expressed and show their expression in the available RNA-seq data used for the verification since the existing expression data limited on the studied conditions. Like the unmatching with known ncRNAs, the homology search in which sequence or structural similarity is limited to known ncRNAs available in the databases. These might be the reasons for the remaining unsupported ncRNAs in almost half of the identified ncRNAs. The increasing of cassava expression data and known ncRNAs in databases might gain more supporting evidence of the remaining unsupported ncRNAs. However, the inevitable false positive by computational prediction was also kept in mind. According to the estimation of RNAz performance, high confident ncRNAs (*P* > 0.9) contained ~1% of false discovery rate and more than 80% of identified ncRNAs (46,526) in this work obtained the high confidence.

### 3.2. Potential Novel lncRNAs Classification and Characterization

Notably, 51,832 of the putative ncRNA loci did not match previously published ncRNAs (Figure 2B). Among them, 2229 ncRNA loci were identified as potential novel lncRNAs, called Me-lncRNAs. They had RNA-seq data support and comprised 62 lincRNAs, 626 lncNATs, and 1541 sense/intronic lncRNAs (see more detail in the Materials and Methods Section 2.3.2). Approximately 93% (2084 loci) of these potential novel lncRNAs were identified with high confidence (*P* > 0.9; Figure S8), and their expression levels were comparable to those of protein-coding genes (Figures S9–S14). Particularly, some of these lncRNAs showed high expression levels, more than the 95th percentile rank of expression. These promising putative lncRNAs, Me-lncRNAs, were then selected for further analysis in the next section.

**Figure 3:**
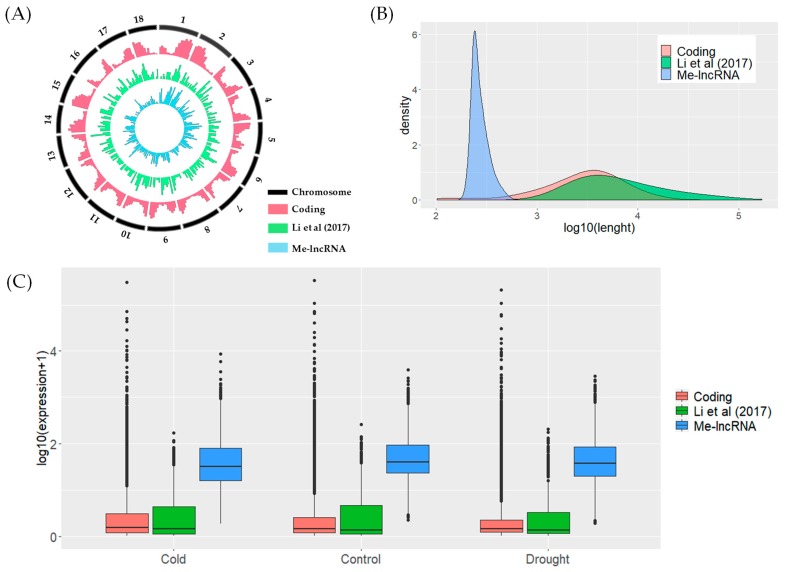
Comparison of protein-coding genes, lncRNA loci from Li et al. [15] and predicted novel lncRNA loci (Me-lncRNAs). (**A**) Distribution of coding genes, lncRNA loci from Li et al. [15] and Me-lncRNAs along 18 chromosomes of cassava. (**B**) Size distribution of coding genes, lncRNA loci from Li et al. [15] and Me-lncRNAs. (**C**) Boxplots of protein-coding genes, lncRNA loci from Li et al. [15] and Me-lncRNAs expression in control, cold and drought conditions.

During the time this research was performed, only one research work on cassava lncRNAs identification, by Li et al. [15], existed. They identified 318 lncRNAs that are responsive to cold and drought stress, using a transcriptome-wide approach. We compared their lncRNA catalog with Me-lncRNAs and, interestingly, found they are quite distinct. The authors suggested that the low number of lncRNAs identified in their work was due to the stringent screening criteria. Generally, an expression level cutoff of 0.5–1 Fragments Per Kilobase of transcript per Million mapped reads (FPKM) has been used for transcriptome-based lncRNA identification [15,56,57]. Although this may not seem very strict, the combination with other screening criteria may filter out ncRNA transcripts expressed at low levels. We observed that the 2229 potential novel lncRNAs (Me-lncRNAs) were distributed evenly across all 18 cassava chromosomes (Figure 3A). Notably, our method could not detect the full length of ncRNA genes as the lncRNAs in Me-lncRNA are shorter than 1000 nt (Figure 3B), which may be related to the prediction window applied. Probably, adjacent windows that did not pass the prediction criteria were filtered out. On the other hand, the transcriptome-based approach is able to identify ncRNAs with full-length transcript boundary [58]. Based on RNA-seq datasets analyzed in this work, expression levels of Me-lncRNAs were mostly higher than those of protein-coding genes and previously identified lncRNAs [15] (Figure 3C). Me-lncRNAs showed high expression levels over the 95th percentile rank of expression (Figures S9–S14). Additionally, we compared Me-lncRNAs with recently published cassava lncRNAs [16,17,18,19] and found no evidence of significant sequence similarity.

### 3.3. Functional Analysis of Potential Novel lncRNAs Involved in Cold and/or Drought Stress

**Figure 4:**
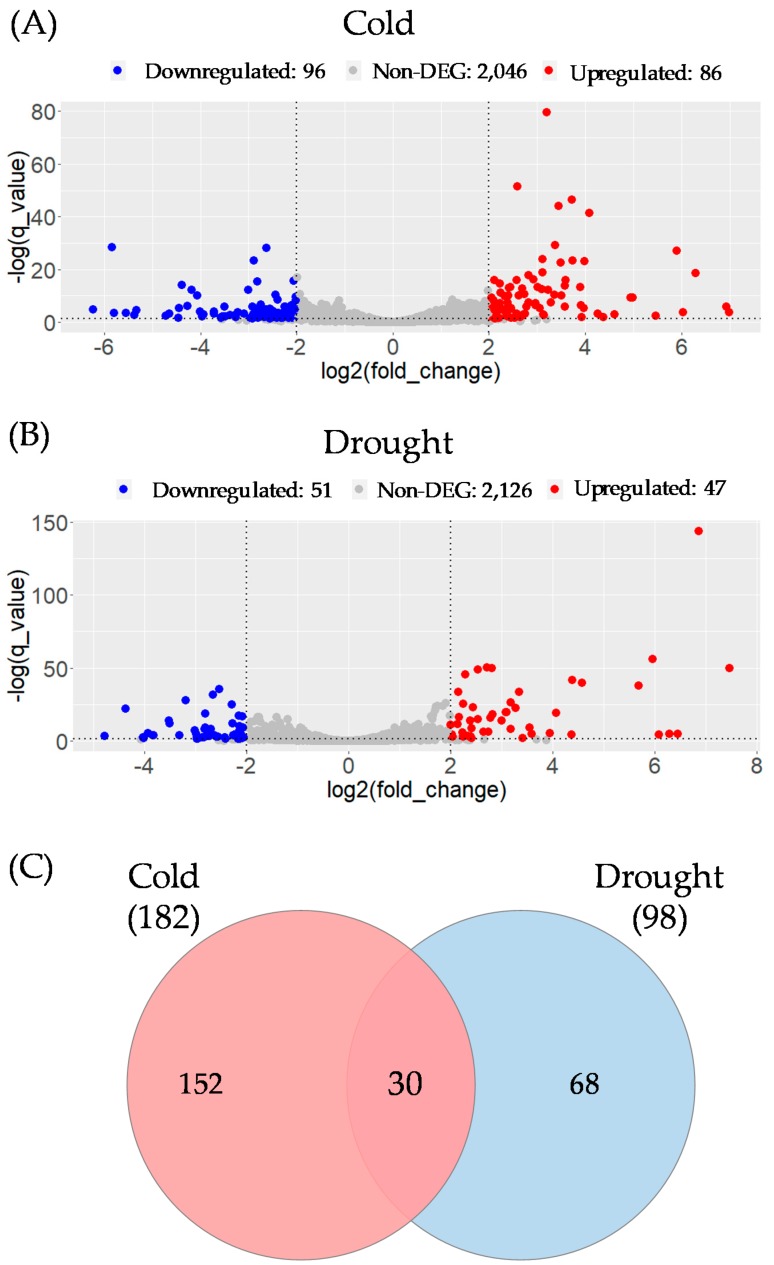
Potential novel lncRNAs with differential expression. Significant differentially expressed lncRNAs under (**A**) cold and (**B**) drought stress in cassava, upregulated or downregulated relative to control. (**C**) Venn-diagram represents differentially expressed lncRNAs under cold and/or drought stress.

Most plants, including cassava, are vulnerable to cold and drought stress. Cold and drought-responsive lncRNAs have been reported in plants [13,59]. Li et al. [15] reported 318 lncRNAs involved in cold/drought stress. Analysis with these RNA-seq datasets revealed 250 of the potential novel lncRNA or Me-lncRNAs, including 13 lincRNAs, 44 lncNATs, and 193 sense/intronic lncRNAs, showed significant differential expression (absolute log2fold change ≥ 2 and q-value ≤ 0.05) in cold and drought stress conditions, relative to controls. In the cold condition, 86 Me-lncRNAs showed increased expression, whereas 96 Me-lncRNAs showed decreased expression (Figure 4A). In the drought condition, 47 and 51 of the Me-lncRNAs were expressed at elevated and lower levels, respectively (Figure 4B). A total of 30 novel lncRNAs showed significant differential expression in both cold and drought conditions (Figure 4C), suggesting they are relevant universal response regulators. Therefore, the remaining 152 and 68 lncRNAs can be considered as possible modulators specifically relating to cold and drought, respectively. The catalog of 250 novel lncRNAs represents interesting candidates for further investigation to gain insights into molecular responses to abiotic stress in cassava.

**Figure 5:**
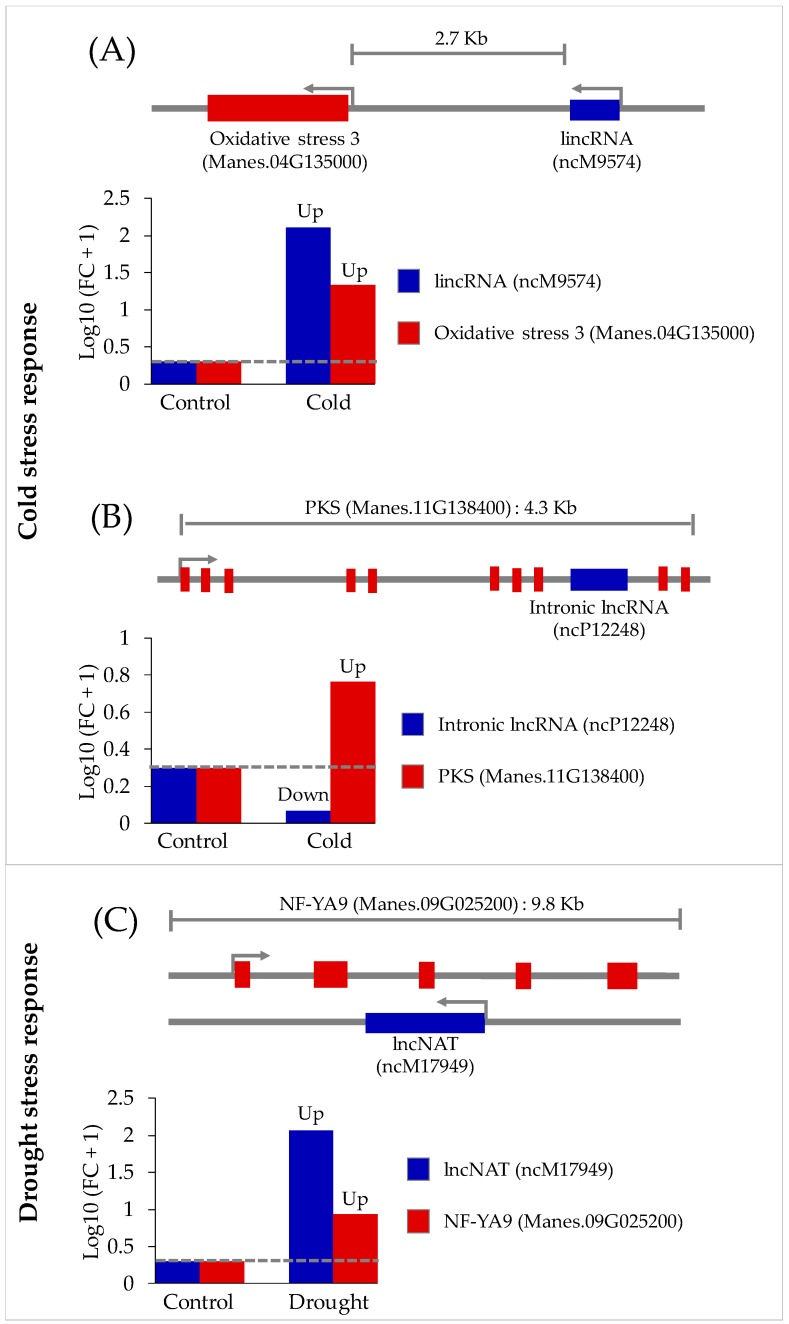
Potential novel lncRNAs that responded to cold or drought stress by *cis*-regulatory relationships with target genes. (**A**) Locus and fold change (FC) of lincRNA, ncM9574 and predicted target gene encoding oxidative stress 3 (Manes.04G135000) in cassava reference genome AM560v6. (**B**) Locus and fold change (FC) of intronic lncRNA, ncP12248 and predicted target gene encoding polyketide synthase enoyl reductase family protein (PKS) (Manes.11G138400), in cassava reference genome AM560v6. (**C**) Locus and fold change (FC) of lncNAT, ncM17949 and predicted target gene-encoding nuclear factor Y subunit A9 (*NF-YA9*) (Manes.09G025200) in cassava reference genome AM560v6. FC_control_ = Expression_control_/Expression_control_; FC_cold_ = Expression_cold_/Expression_control_; FC_drought_ = Expression_drought_/Expression_control_.

Long non-coding RNAs exert *cis*- and *trans*-regulatory modes in controlling gene expression. For the *cis*-regulatory function, genes at proximal locations are considered to be possible targets of relevant lncRNAs. We collected 635 proximal genes located within 10 Kilobases of individual 250 novel lncRNAs either upstream or downstream on the same or opposite strands. The expression levels of these 635 protein-coding genes, based on Li et al. [15] RNA-seq data, were then observed (Figures S15–S17). It was found that 98 proximal target genes with plausibly *cis*-regulatory control in cold or drought stress response were significant differentially expressed (absolute log2fold change ≥ 2 and q-value ≤ 0.05). Some of these interacting lncRNA-target gene pairs are shown in Figure 5. These lncRNA loci are promising candidates for further experimental characterization. In the cold condition, the expression of the Manes.04G135000 gene, located approximately 2.7 Kb downstream of the novel ncM9574 lncRNA, was observed to be significantly elevated with increased expression of ncM9574 (Figure 5A; Figure S18). In the cassava genome, Manes.04G135000 is annotated as oxidative stress 3. Low-temperature/chilling-induced oxidative stress has been reported in wheat cultivars [60]. Thus far, there are no annotated and characterized oxidative stress-responsive cassava lncRNAs. However, there have been a few reports on lncRNAs involved in oxidative stress processes in plants such as Medicago, tobacco, and poplar [61,62,63]. 

Another interesting lncRNAs acting as a *cis*-regulator controlling target genes under cold stress is ncP12248. This sense/intronic lncRNA is located in an intronic region of Manes.11G138400, which is annotated for polyketide synthase, an enoyl reductase family protein (PKS). Inverse differential expression of the ncP12248-Manes.11G138400 pair was observed in the cold condition (Figure 5B; Figure S19). There is limited information on plant lncRNAs modulating PKS family genes in abiotic stress conditions. Recently, molecular characterization revealed that PKS family genes were induced by drought and some other abiotic stress, suggesting their significance in abiotic stress response [64].

For drought stress, Manes.09G025200, annotated as a nuclear factor Y subunit A9 (*NF-YA9*) encoding gene, was predicted to be a target of another novel lncRNA, ncM17949. Expression levels of these two molecules increased similarly (Figure 5C). Co-expression of *NF-YA9* mRNA and lncRNAs in autotetraploid cassava under drought stress has been reported [19]. Nuclear factor Y (NF-Y) has been shown to confer drought tolerance in maize [65], Arabidopsis [66], and rice [67]. In this work, we observed that ncM17949 is located on chromosome 9 of cassava AM560v6, with Manes.09G025200 on the opposite strand. As mentioned earlier, elevated expression of both ncM17949 and Manes.09G025200 was observed based on strand-specific RNA-seq reads (Figure S20). Moreover, ncM17949 and mRNA of Manes.09G025200 showed direct binding interaction (Figure S21).

**Figure 6:**
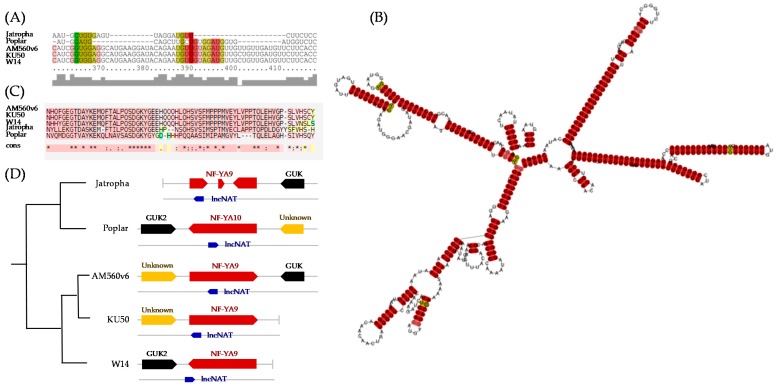
Characteristics of the putative long noncoding natural antisense transcripts (lncNAT), ncM17949. (**A**) Consensus alignment of the lncNAT ncM17949 from cassava AM560, at position 370–410, and its orthologous sequences from cassava KU50, cassava W14, Jatropha, and poplar annotated by LocARNA tool. (**B**) Consensus secondary structure of lncNAT, ncM17949 in cassava. (**C**) Similarity of nuclear factor Y protein sequences in cassava AM560, KU50, W14, Jatropha, and poplar annotated by T-Coffee tool. Alignments in pink, yellow, and green colors respectively, represent good, average, and bad consistency among the amino acid sequences. (**D**) Orthologs and syntenic features of lncNAT, ncM17949 in Jatropha, poplar, and cassava cultivars AM560v6, KU50, and W14.

Recently, syntenic lncRNAs have been reported in several organisms, including plants [68,69,70]. The conservation of genomic positions of lncRNAs and their target genes across related species has been investigated to consolidate their interactions [70]. In this study, the ncM17949-Manes.09G025200 interacting pair was investigated by sequence analysis. Results show the ncM17949 sequence shares relatively low similarity with corresponding intergenic sequences in poplar, Jatropha, and cassava cultivars W14 and KU50 (Figure 6A). Even though four of them (AM560v6, W14, KU50, and Jatropha) are Euphobiaceae plants, low primary sequence similarity among them is not unexpected in terms of ncRNA sequence conservation [71]. Notably, the consensus stable secondary structure of ncM17949 lncRNA homologs in cassava was predicted (Figure 6B). Interestingly, the homologs were found to be located in a particular genomic region opposite the respective NF-Y encoding sequences. The NF-Y encoding genes appeared to share reasonable amino acid sequence similarities (Figure 6C). Moreover, the flanking genes, a hypothetical protein and GUK (guanylate kinase), of ncM17949 lncRNA homologs were positionally equivalent among these closely related species (Figure 6D). Although other copies of NF-Y encoding gene sequences were found in the AM560v6 genome, only the Manes.09G025200 *NF-YA9* sequence was flanked by these two genes. These results revealed syntenic conservation of ncM17949 and Manes.09G025200 in Euphobiaceae genomes. To our knowledge, ncM17949 is the first cassava lncRNA reported to have positionally conserved synteny. 

**Figure 7:**
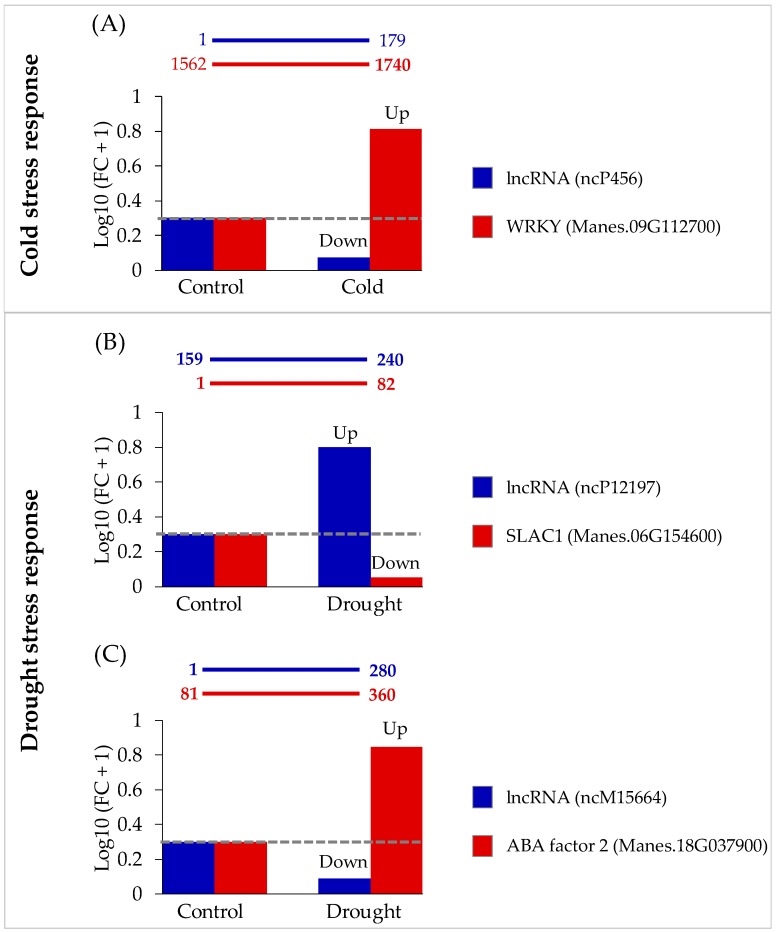
Potential novel lncRNAs that responded to cold and drought stress by a *trans*-regulatory relationship through direct binding to mRNAs of target genes. (**A**) Binding region of lncRNA ncP456 at position 1–179 and mRNA of target gene encoding WRKY DNA-binding protein 33 (Manes.09G112700) at position 1562–1740, including their fold change (FC) under control and cold conditions. (**B**) Binding region of lncRNA ncP12192 at position 159–240 and mRNA of target gene encoding SLAC1 (Manes.06G154600) at position 1–82, including their fold change (FC) under control and drought conditions. (**C**) Binding region of lncRNA ncM15664 at position 1–280 and mRNA of target gene encoding ABA responsive elements-binding factor2 (Manes.18G037900) at position 81–360, including their fold change (FC) under control and drought conditions. FC_control_ = Expression_control_/Expression_control_; FC_cold_ = Expression_cold_/Expression_control_; FC_drought_ = Expression_drought_/Expression_control_.

For *trans*-regulatory relationships, lncRNAs interact with their targets at distal loci. To perform their functions, regulatory complexes are formed by interaction with proteins and/or RNAs as described in detail elsewhere [1]. Direct binding between the novel lncRNAs and their target mRNAs were predicted: 182 lncRNAs that were differentially expressed in the cold condition interacted with 2956 protein-coding genes. Those that were differentially expressed in the drought condition, 98 lncRNAs in total, interacted with 802 protein-coding genes. For the cold condition, GO enrichment analysis of target genes showed dominant functions (FDR ≤ 0.05) in the molecular function category related to protein binding (GO:0005515). Among the enriched genes categorized in GO:0005515 were genes coding for several transcription factor protein families. Interestingly, four WRKY transcription factor gene sequences were found, namely, Manes.02G011500, Manes.04G102600, Manes.11G066500, and Manes.09G112700. The WRKY family transcription factors have been widely studied in various plants under cold stress [72,73,74]. Some TFs in the WRKY family have been reported as hubs in plant co-expression networks under abiotic stress, including cold stress [75]. In this work, a novel putative lncRNA (ncP456) was predicted to directly bind complementarily with Manes.09G112700, annotated as WRKY DNA-binding protein 33 (Figure S22). In addition, analysis with Li et al.’s [15] RNA-seq data for cold condition revealed inverse differential expression of ncP456 and its target. The expression of ncP456 decreased, while that of Manes.09G112700 increased significantly (Figure 7A; Figure S23). There was no evidence of significant sequence similarity between Manes.09G112700 and WRKY proteins previously reported to interact with stress-responsive lncRNAs [19]. Furthermore, for the drought condition, enriched functions of the 802 target genes of 98 lncRNAs were dominant (FDR ≤ 0.2) in 15 GO categories. Among these enriched gene groups those involved in the regulation of stomatal opening (GO:1902456) and abscisic acid (ABA)-responsive ones (GO:0009737) have been associated with drought response in plants [76]. lncRNAs regulating genes involved in ABA signaling and stomatal closure have been reported in Arabidopsis and tomato [13,59]. In this work, ncP12197 was predicted to bind directly with Manes.06G154600 coding for SLAC1 protein (Figure S24), which is reportedly involved in regulating guard cells for stomatal opening/closure during drought [77]. Besides, stomatal closure is triggered by elevated ABA level in guard cells in the drought condition [78]. RNA-seq data analysis revealed that ncP12197 expression increased, while that of Manes.06G154600 decreased (Figure 7B; Figure S25). In the drought condition, *SLAC1* is downregulated to reduce water loss. RNA-seq data analysis showed that ncM15664 expression decreased (Figure S26), whereas that of its predicted target (Manes.18G037900; ABA-responsive elements-binding factor 2) increased significantly in the drought condition (Figure 7C; Figure S27). Direct binding of lncRNAs to mRNA targets with dissimilar expression patterns has been reported [79]. It has been suggested that lncRNA binding to mRNA targets would reduce free mRNA transcripts available for translation. The decreased expression of ncp456 lncRNA in the cold condition enabled the increase in WRKY TF gene expression, an indication of the controlling ability of lncRNAs over related genes. In the drought condition, likewise, decreased expression of ncM15664 with increased expression of target ABA-responsive element-binding factor 2 (Manes.18G037900) was observed, in addition to increased ncp12197 with decreased target *SLAC1* (Manes.06G154600) expression. It suggested the potential involvement of these lncRNAs under cold or drought stress response in cassava. Although homologs of these target genes have been reported in previous works [16,19], ours represents the first report of their interaction with associated lncRNAs. This suggests the efficacy of genome-wide comparative approach for lncRNA identification, expanding the boundary for discovering plant lncRNAs responding to abiotic stress and more potential cold and/or drought stress-response lncRNAs beyond those previously reported lncRNAs from the transcriptome-based approach by Li et al. [15].

**Figure 8:**
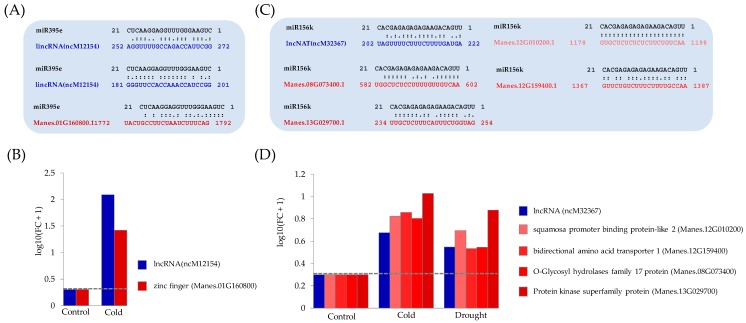
Potential novel lncRNA responded in cold and/or drought stress conditions by a *trans*-regulatory relationship through miRNA target mimicry. (**A**) Binding site of miR395e on lncRNA, ncM12154, and zinc finger (CCCH-type) family protein mRNA (Manes.01G160800.1). (**B**) Fold change (FC) of lncRNA, ncM12154, and target gene encoded for zinc finger (CCCH-type) family protein (Manes.01G160800) under control and cold stress conditions. *(***C**) Binding site of miR156k on lncRNA (ncM32367) and SQUAMOSA-promoter binding protein-like 2 mRNA (Manes.12G010200.1), bidirectional amino acid transporter 1 mRNA (Manes.12G159400.1), O-Glycosyl hydrolases family 17 protein mRNA (Manes.08G073400.1), and protein kinase superfamily protein mRNA (Manes.13G029700.1). (**D**) Fold change (FC) of lncRNA, ncM32367, protein-coding gene encoded for SQUAMOSA-promoter binding protein-like 2 (Manes.12G010200), bidirectional amino acid transporter 1 (Manes.12G159400), O-Glycosyl hydrolases family 17 protein (Manes.08G073400), and protein kinase superfamily protein (Manes.13G029700) under control, cold, and drought stress conditions. FC_control_ = Expression_control_/Expression_control_; FC_cold_ = Expression_cold_/Expression_control_; FC_drought_ = Expression_drought_/Expression_control_.

One regulatory mechanism of lncRNA functions is by target mimicry: lncRNA acts as a sponge of miRNA (miRNA binding to lncRNA rather than targeted mRNA, which then allows targeted mRNA to be translated), thereby preventing miRNA from binding to mRNA, which results in the translation process of mRNA [1]. We found that 21 of 250 differentially expressed putative lncRNAs could function as target mimics, plausibly targeting 14 miRNA families, namely, miR11891, miR1446, miR156, miR159, miR168, miR169, miR171, miR172, miR2111, miR393, miR395, miR399, miR403, and miR482. Most of them, except miR11891, miR1446, miR2111, and miR482, were reported as stress-induced miRNAs [80]. We found that ncM12154 could bind to the miR395e cassava sequence at two specific locations (Figure 8A). MiR395e homologs in different plants have been associated with stress responses. For example, miR395e specifically was cold-induced in wheat [80]. Interestingly, it was also observed that cassava miR395e was able to form base-pairing with a particular region of Manes.01G160800 cassava zinc finger (CCCH-type) mRNA (Figure 8A). Moreover, binding interaction between ncM12154 and mRNA transcript of Manes.01G160800 was observed (Figure S28). RNA-seq data analysis showed that, in the cold condition, expression levels of both ncM12154 and Manes.01G160800 mRNA increased relative to controls (Figure 8B; Figure S29). This increased ncM12154 expression, probably forms lncRNAs-miRNAs interaction, and avoids Manes.01G160800 zinc finger degradation, resulting in increased zinc finger mRNA level in the cold condition.

It has been proven that miRNAs can bind to more than one target. The putative ncM32367 lncRNA could bind to the cassava miR156k (Figure 8C). Also, miR156k was predicted to bind to four cassava mRNAs: Manes.12G010200.1, Manes.12G159400.1, Manes.08G073400.1, and Manes.13G029700.1 (Figure 8C). These mRNA sequences were annotated for the Squamosa-Promoter binding protein-Like 2 (*SPL*) gene, bidirectional amino acid transporter 1 gene, O-Glycosyl hydrolase family 17 protein gene, and protein kinase superfamily, respectively. Their expression levels increased in both the cold and drought conditions (Figure 8D; Figure S30). MiR156k homologs responsive to cold and drought stress have been identified in various plants, including rice, wheat, maize, barley, and Arabidopsis [80]. The functionally diverse SQUAMOSA-promoter binding protein-like family of transcription factors was firstly identified in snapdragon [81] and regulates various growth and developmental processes in plants, including hormone signaling and stimuli responses [82]. The *SPL* gene was reported as a target of cassava miR156, which is known to be involved in starch biosynthesis and plant development [83]. Recently, lncRNA was also identified as a miR156k target mimicry of the *SPL* transcript in cassava under drought stress [16]. Although the detail of this particular lncRNA sequence was not provided, we observed that the corresponding *SPL* locus (Manes.09G032800) did not overlap with its paralogous *SPL*, Manes.12G010200.1, detected in this work. That was probably because the target *SPL* locus (Manes.09G032800) was not significant differentially expressed based on the RNA-seq data analysis. Diverged regulatory relationships between SPL proteins and their targets have been suggested based on the evolution of this protein family [84]. Hence, the regulatory relationship of cassava miR156k:ncM32367 lncRNA:*SPL* Manes.12G010200.1 requires further characterization.

## 4. Conclusions

In this work, we reported on computational genomic screening and transcriptomic analysis of the cassava AM560 genome for ncRNAs, particularly lncRNAs’ identification. This cassava strain has been widely used for transcriptomic studies. A total of 56,840 putative ncRNA candidates were identified, approximately 50% of which were supported by previously reported ncRNAs and 74 publicly available RNA-seq datasets. Interestingly, we found 2229 potential novel lncRNAs, 250 of which were significantly differentially expressed, together with their potential target genes, in cold and/or drought stress conditions. Our study identified some potential novel regulatory modulators and suggested interesting ncRNA candidates involved in abiotic stress responses in cassava for further experimental validation. In addition, the landscape of putative ncRNAs is a valuable resource for genome annotation, transcriptome data integration, and understanding gene regulation in cassava. We suggest the advantage of a comparative approach for ncRNAs’ discovery, which was overlooked by a widely used transcriptome-based method. However, the false positive rate is a significant issue for this approach [85] and further experimental validation is still required. In addition, systematic analysis by reconstructing a regulatory network between ncRNAs and their target genes is recommended for more understanding in transcriptional regulation under stress responses in cassava. We also provided valuable data resources for those interested in experimental investigation. Our results would be beneficial and shed light on cassava yield improvement against abiotic stress under the global climate change crisis.

## Supplementary Materials

The following are available online at https://www.mdpi.com/2073-4425/11/4/366/s1: Figure S1: 56,840 predicted ncRNAs from RNAz tool with *P* > 0.5; Figure S2: Comparison of expression level between unmatched ncRNAs with known ncRNAs and protein coding genes in cassava RNA-seq data from Li [15]; Figure S3: Comparison of expression level between unmatched ncRNAs with known ncRNAs and protein coding genes in cassava RNA-seq data from Wang [32]; Figure S4: Comparison of expression level between unmatched ncRNAs with known ncRNAs and protein coding genes in cassava RNA-seq data from Wilson [39]; Figure S5: Comparison of expression level between unmatched ncRNAs with known ncRNAs and protein coding genes in cassava RNA-seq data from Pootakham [41]; Figure S6: Comparison of expression level between unmatched ncRNAs with known ncRNAs and protein coding genes in CBSV-resistant cassava RNA-seq data from Amuge [40]; Figure S7: Comparison of expression level between unmatched ncRNAs with known ncRNAs and protein coding genes in CBSV-susceptible cassava RNA-seq data from Amuge [40]; Figure S8: Expression supporting and confidence of potential novel lncRNAs; Figure S9: Comparison of expression level between Me-lncRNAs and protein coding genes in cassava RNA-seq data from Li [15]; Figure S10: Comparison of expression level between Me-lncRNAs and protein coding genes in cassava RNA-seq data from Wang [32]; Figure S11: Comparison of expression level between Me-lncRNAs and protein coding genes in cassava RNA-seq data from Wilson [39]; Figure S12: Comparison of expression level between Me-lncRNAs and protein coding genes in cassava RNA-seq data from Pootakham [41]; Figure S13: Comparison of expression level between Me-lncRNAs and protein coding genes in CBSV-resistant cassava RNA-seq data from Amuge [40]; Figure S14: Comparison of expression level between Me-lncRNAs and protein coding genes in CBSV-susceptible cassava RNA-seq data from Amuge [40]; Figure S15: Volcano plot represents differentially expressed coding genes under cold stress of cassava; Figure S16: Volcano plot represents differentially expressed coding genes under drought stress of cassava; Figure S17: Venn-diagram represents differentially expressed coding genes under cold and/or drought stress of cassava; Figure S18: RNA-seq read alignment on ncM9574 and its target; Figure S19: RNA-seq read alignment on ncP12248 and its target; Figure S20: RNA-seq read alignment on ncM17949 and its target; Figure S21: Direct binding of ncM17949 and its target; Figure S22: Direct binding of ncP456 and its target; Figure S23: RNA-seq read alignment on ncP456 and its target; Figure S24: Direct binding of ncP12197 and its target; Figure S25: RNA-seq read alignment on ncP12197 and its target; Figure S26: RNA-seq read alignment on ncM15664 and its target; Figure S27: Direct binding of ncM15664 and its target; Figure S28: Direct binding of ncM12154 and its target; Figure S29: RNA-seq read alignment on ncM12154 and its target; Figure S30: RNA-seq read alignment on ncM32367 and its target.

## Glossary

cassava lncRNAs (https://planet.kmutt.ac.th/Me-lncRNAs).
