# Supplementary material for: Genomic and Transcriptomic Analysis Identified Novel Putative Cassava lncRNAs Involved in Cold and Drought Stress
Source: Genes (Basel). 2020 Mar 28;11(4):366. doi: 10.3390/genes11040366 (PMC7230406; doi:10.3390/genes11040366)
Supplement: Supplementary file 1 [file genes-11-00366-s001.pdf]

Supplementary figures

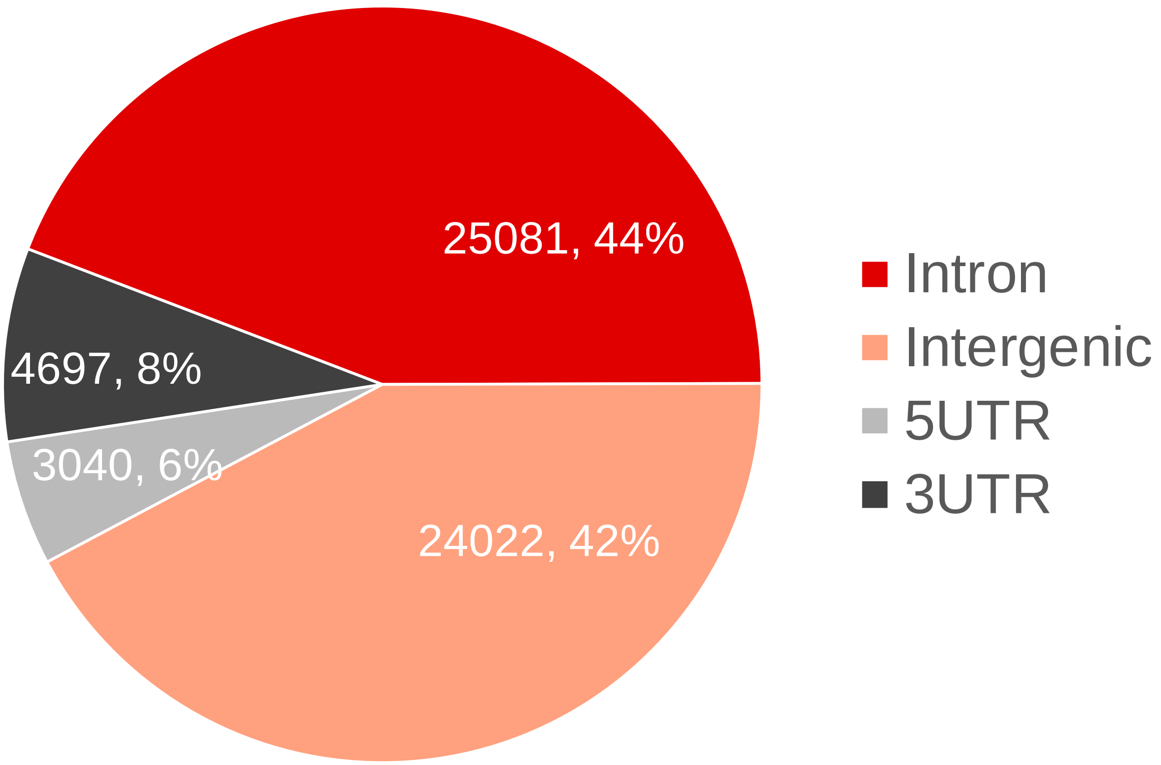

Figure S1: 56,840 predicted ncRNAs from RNAz tool with $P > 0.5$.

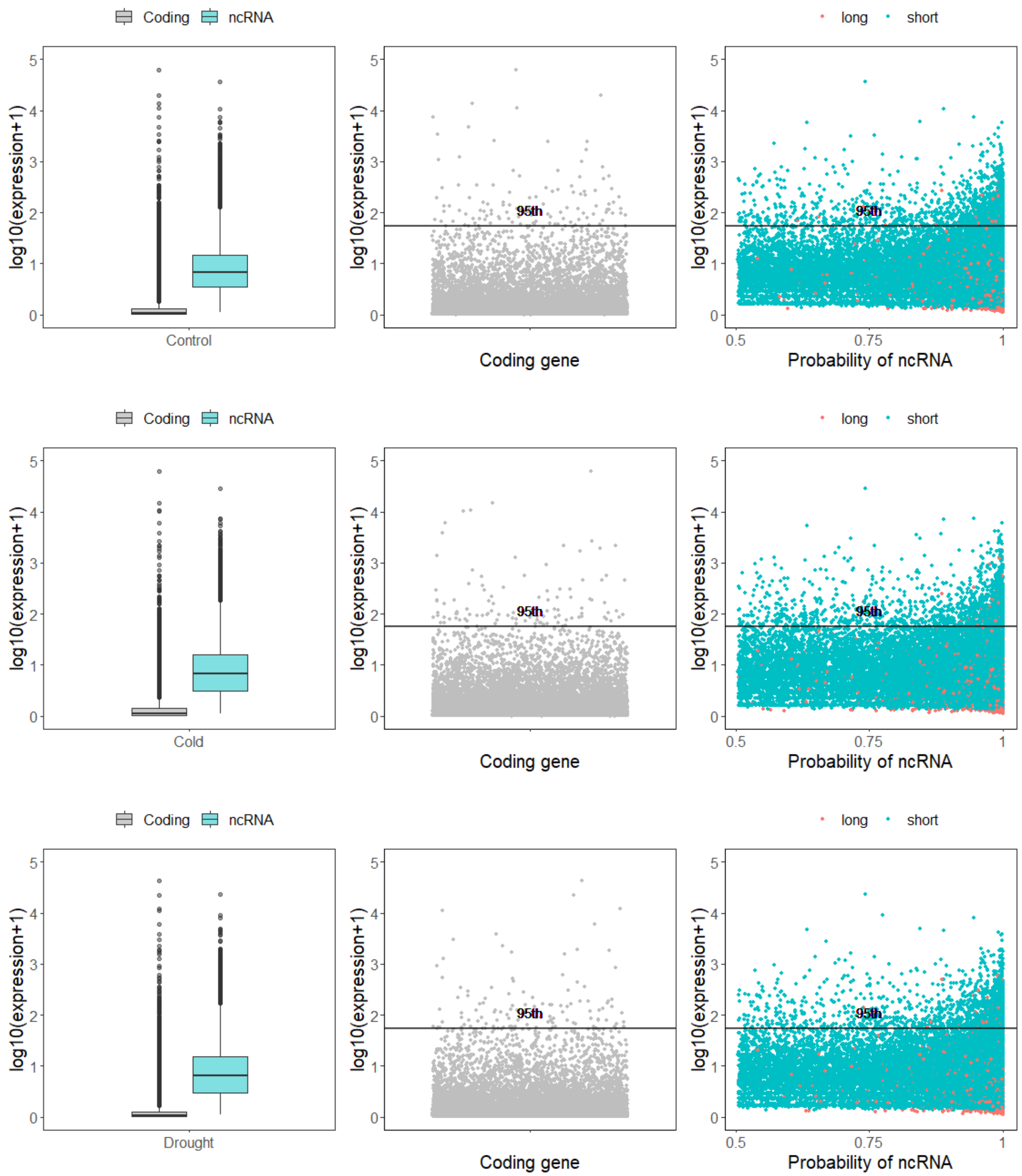

Figure S2: Comparison of expression level between unmatched ncRNAs with known ncRNAs and protein coding genes in cassava RNA-seq data from Li [15]. Y-axis represents expression level with normalization by GeTMM. The left graph represents boxplot of expression level in coding genes and unmatched ncRNAs. The middle graph represents scatterplot of expression distribution in coding genes. The right graph represents scatterplot of expression distribution in short and long unmatched ncRNAs, respectively. X-axis in the right graph determined the confidence (probability to be ncRNA) of unmatched ncRNA according to RNAz tool. Black line at y-intercept denotes 95th percentile rank of expression.

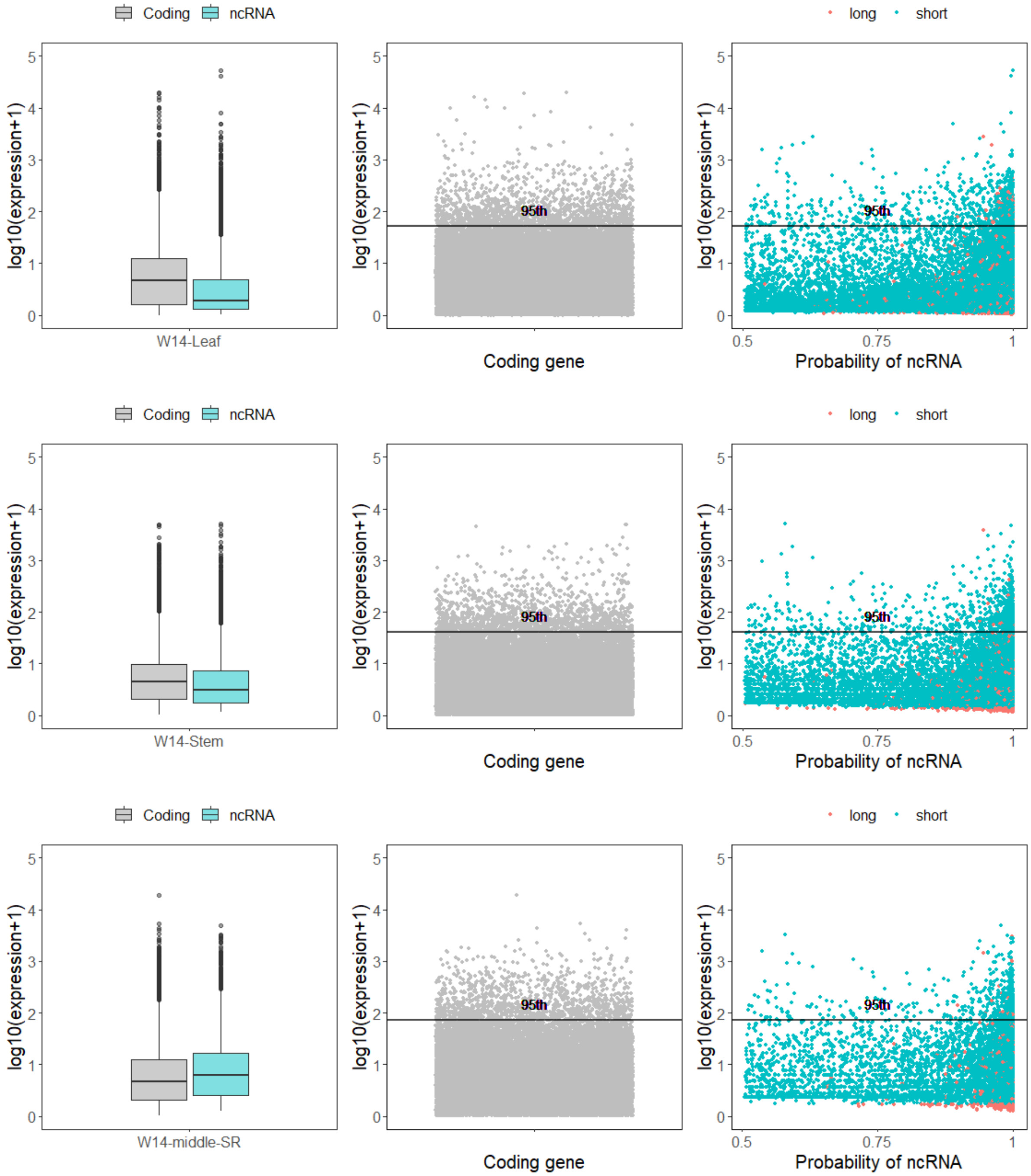

Figure S3: Comparison of expression level between unmatched ncRNAs with known ncRNAs and protein coding genes in cassava RNA-seq data from Wang [32]. Y-axis represents expression level with normalization by GeTMM. The left graph represents boxplot of expression level in coding genes and unmatched ncRNAs. The middle graph represents scatterplot of expression distribution in coding genes. The right graph represents scatterplot of expression distribution in short and long unmatched ncRNAs, respectively. X-axis in the right graph determined the confidence (probability to be ncRNA) of unmatched ncRNA according to RNAz tool. Black line at y-intercept denotes 95th percentile rank of expression.

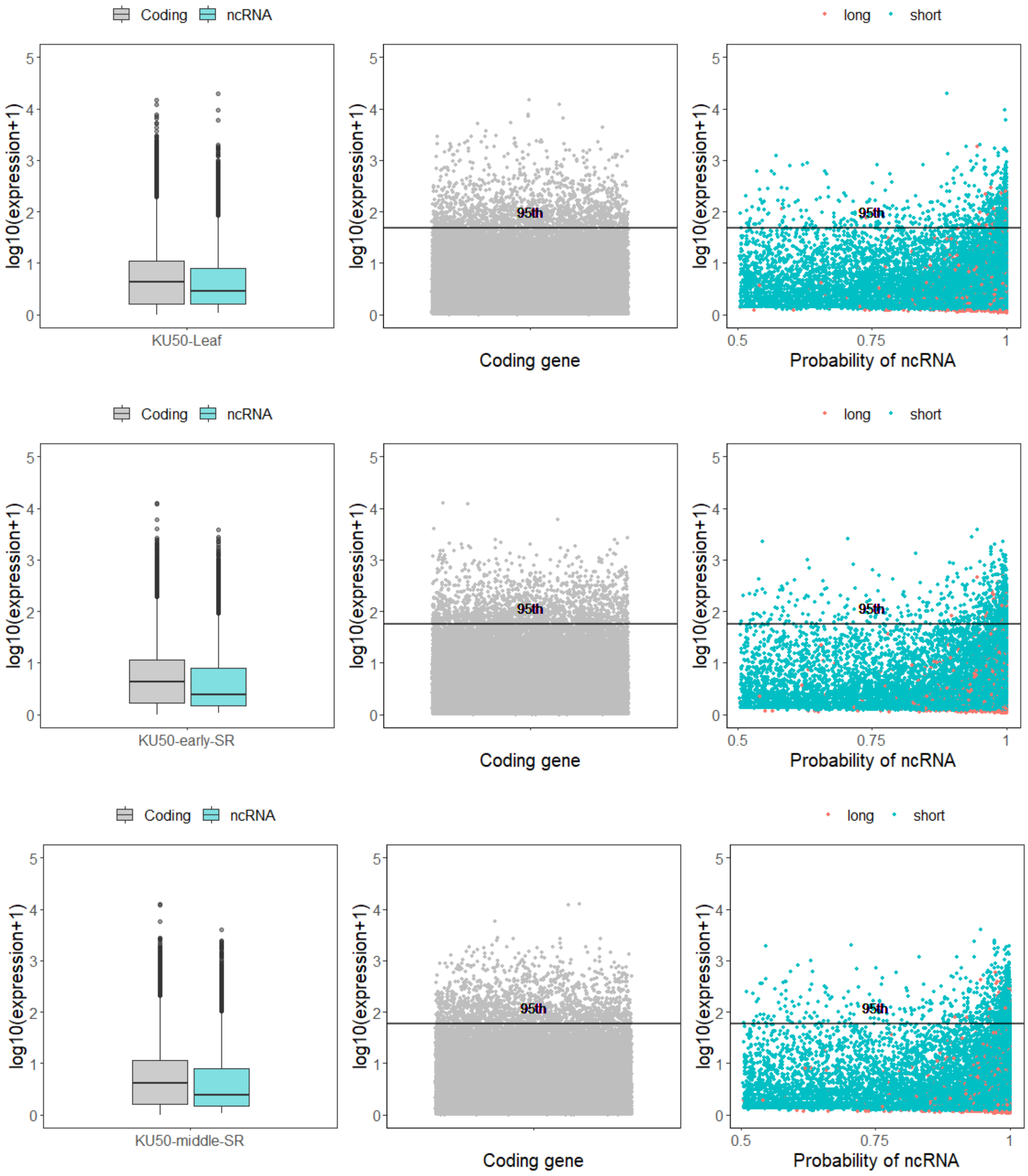

Figure S3: Comparison of expression level between unmatched ncRNAs with known ncRNAs and protein coding genes in cassava RNA-seq data from Wang [32]. Y-axis represents expression level with normalization by GeTMM. The left graph represents boxplot of expression level in coding genes and unmatched ncRNAs. The middle graph represents scatterplot of expression distribution in coding genes. The right graph represents scatterplot of expression distribution in short and long unmatched ncRNAs, respectively. X-axis in the right graph determined the confidence (probability to be ncRNA) of unmatched ncRNA according to RNAz tool. Black line at y-intercept denotes 95th percentile rank of expression (continue).

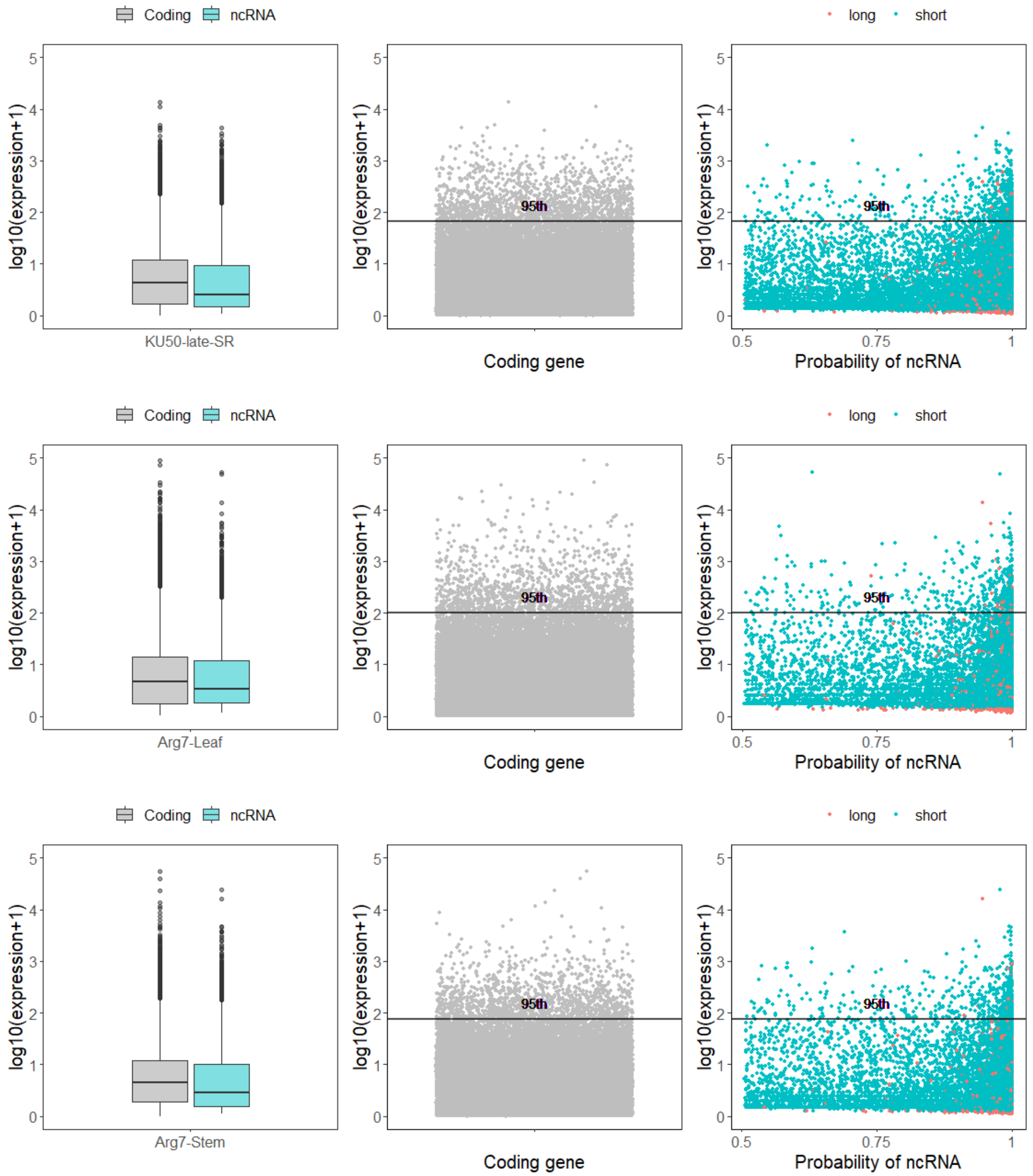

Figure S3: Comparison of expression level between unmatched ncRNAs with known ncRNAs and protein coding genes in cassava RNA-seq data from Wang [32]. Y-axis represents expression level with normalization by GeTMM. The left graph represents boxplot of expression level in coding genes and unmatched ncRNAs. The middle graph represents scatterplot of expression distribution in coding genes. The right graph represents scatterplot of expression distribution in short and long unmatched ncRNAs, respectively. X-axis in the right graph determined the confidence (probability to be ncRNA) of unmatched ncRNA according to RNAz tool. Black line at y-intercept denotes 95th percentile rank of expression (continue).

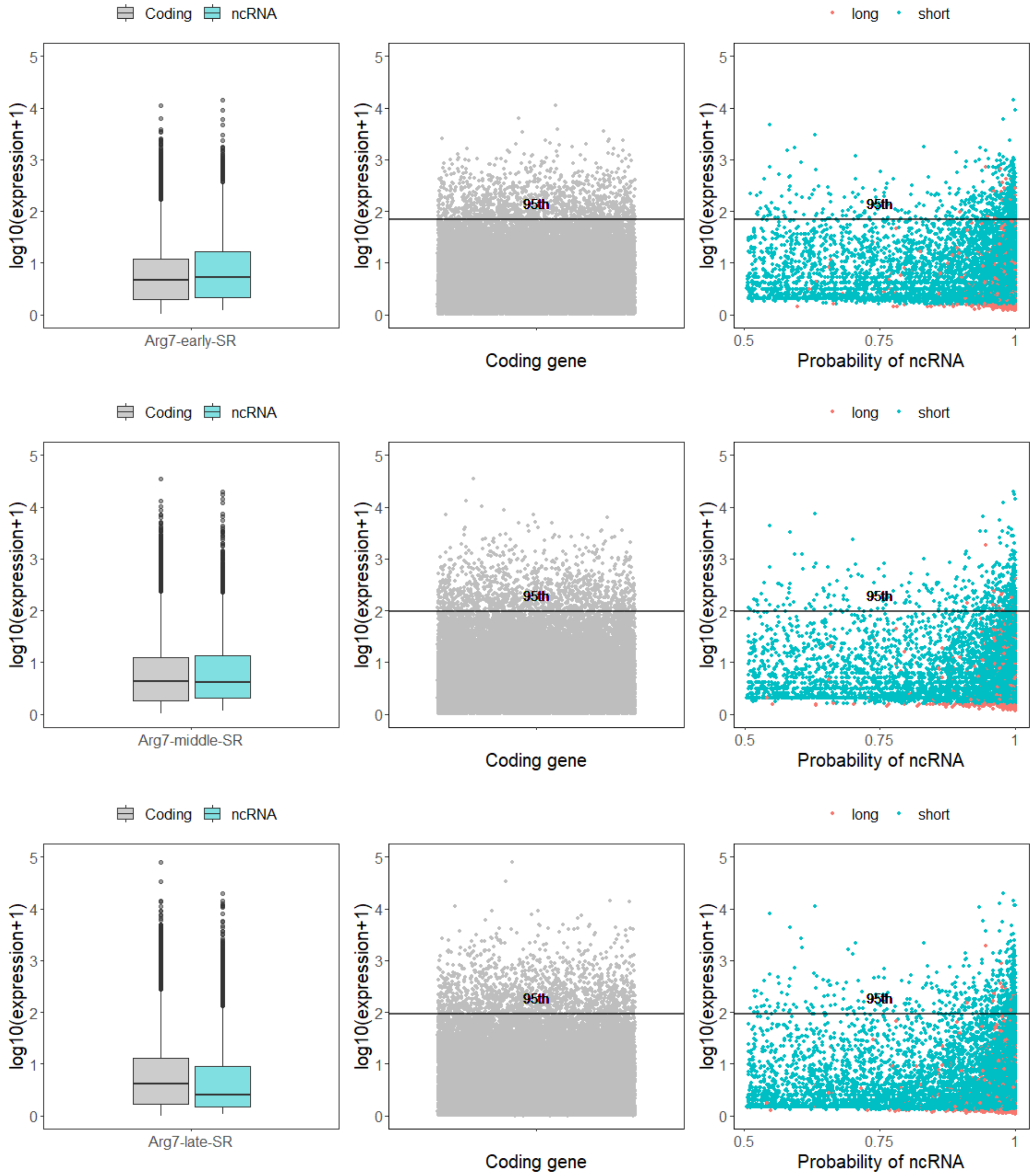

Figure S3: Comparison of expression level between unmatched ncRNAs with known ncRNAs and protein coding genes in cassava RNA-seq data from Wang [32]. Y-axis represents expression level with normalization by GeTMM. The left graph represents boxplot of expression level in coding genes and unmatched ncRNAs. The middle graph represents scatterplot of expression distribution in coding genes. The right graph represents scatterplot of expression distribution in short and long unmatched ncRNAs, respectively. X-axis in the right graph determined the confidence (probability to be ncRNA) of unmatched ncRNA according to RNAz tool. Black line at y-intercept denotes 95th percentile rank of expression (continue).

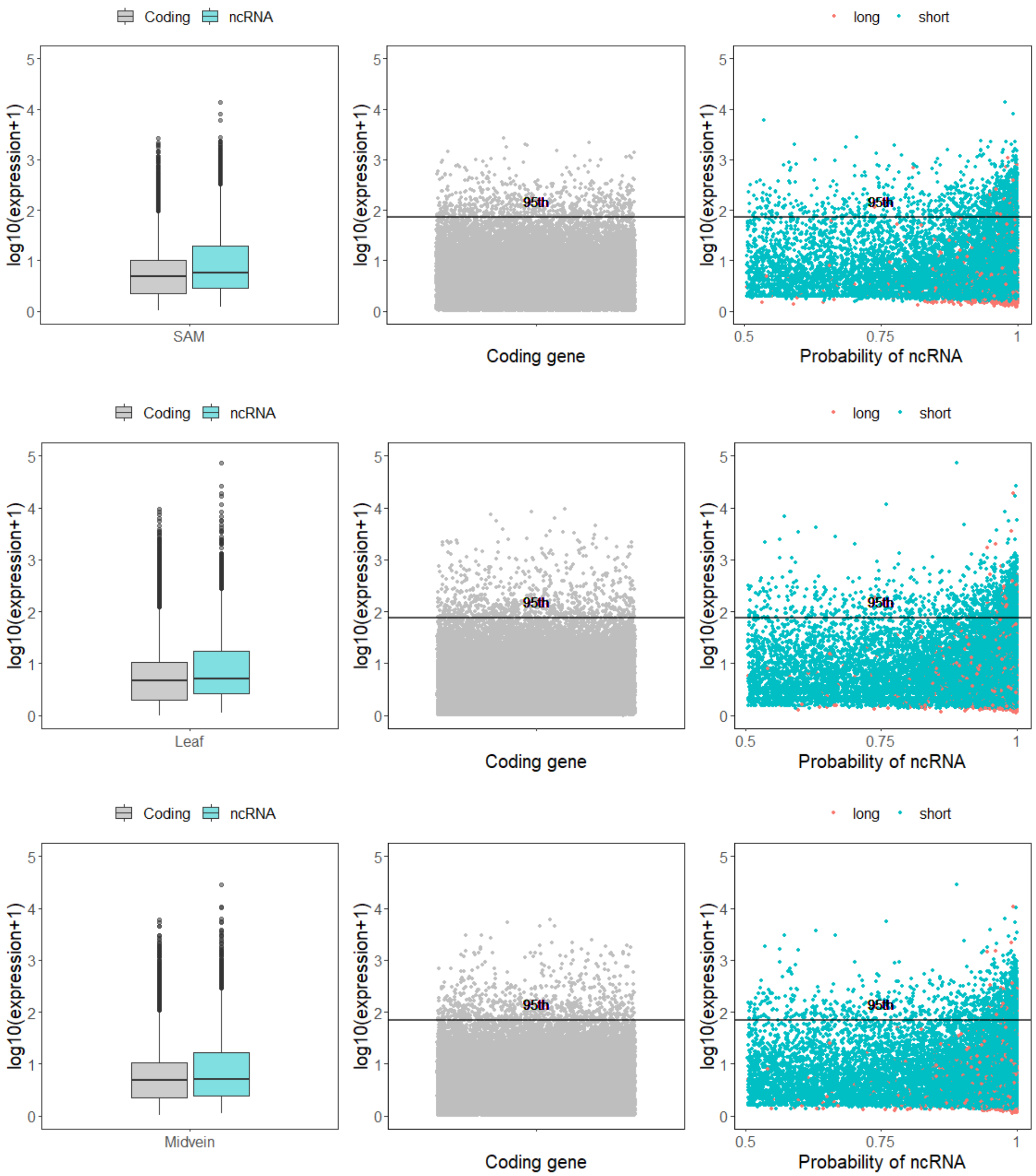

Figure S4: Comparison of expression level between unmatched ncRNAs with known ncRNAs and protein coding genes in cassava RNA-seq data from Wilson [39]. Y-axis represents expression level with normalization by GeTMM. The left graph represents boxplot of expression level in coding genes and unmatched ncRNAs. The middle graph represents scatterplot of expression distribution in coding genes. The right graph represents scatterplot of expression distribution in short and long unmatched ncRNAs, respectively. X-axis in the right graph determined the confidence (probability to be ncRNA) of unmatched ncRNA according to RNAz tool. Black line at y-intercept denotes 95th percentile rank of expression.

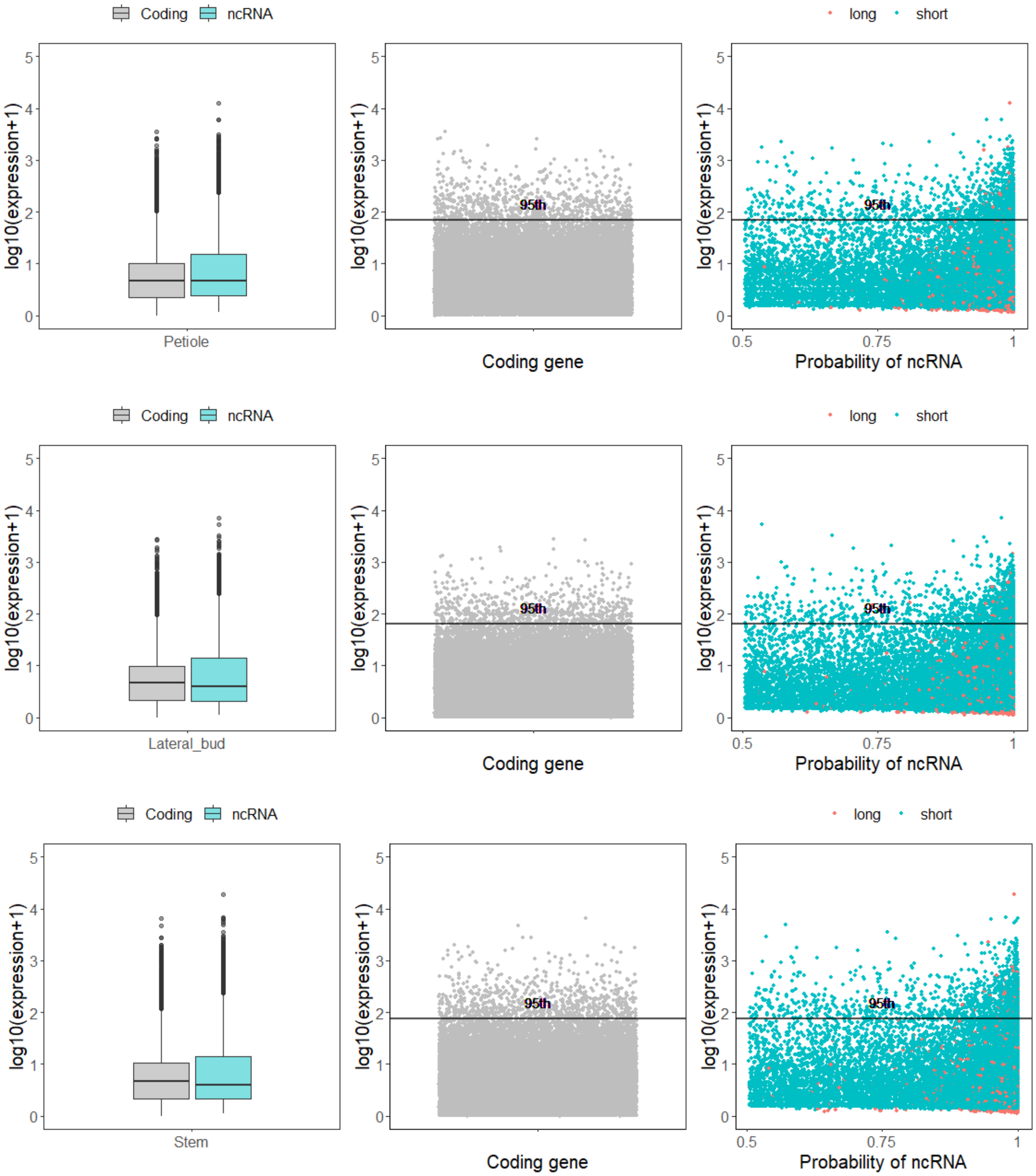

Figure S4: Comparison of expression level between unmatched ncRNAs with known ncRNAs and protein coding genes in cassava RNA-seq data from Wilson [39]. Y-axis represents expression level with normalization by GeTMM. The left graph represents boxplot of expression level in coding genes and unmatched ncRNAs. The middle graph represents scatterplot of expression distribution in coding genes. The right graph represents scatterplot of expression distribution in short and long unmatched ncRNAs, respectively. X-axis in the right graph determined the confidence (probability to be ncRNA) of unmatched ncRNA according to RNAz tool. Black line at y-intercept denotes 95th percentile rank of expression (continue).

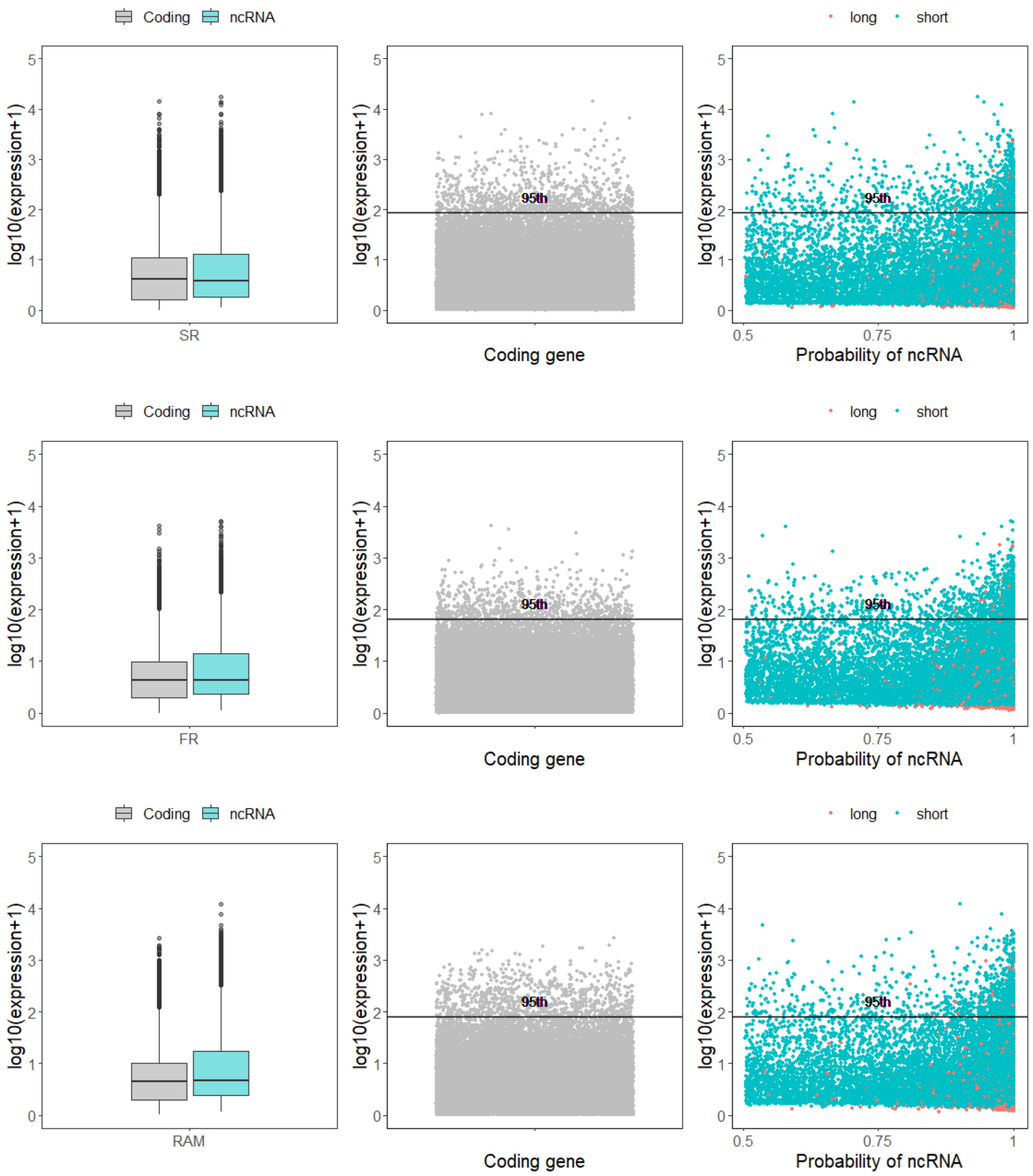

Figure S4: Comparison of expression level between unmatched ncRNAs with known ncRNAs and protein coding genes in cassava RNA-seq data from Wilson [39]. Y-axis represents expression level with normalization by GeTMM. The left graph represents boxplot of expression level in coding genes and unmatched ncRNAs. The middle graph represents scatterplot of expression distribution in coding genes. The right graph represents scatterplot of expression distribution in short and long unmatched ncRNAs, respectively. X-axis in the right graph determined the confidence (probability to be ncRNA) of unmatched ncRNA according to RNAz tool. Black line at y-intercept denotes 95th percentile rank of expression (continue).

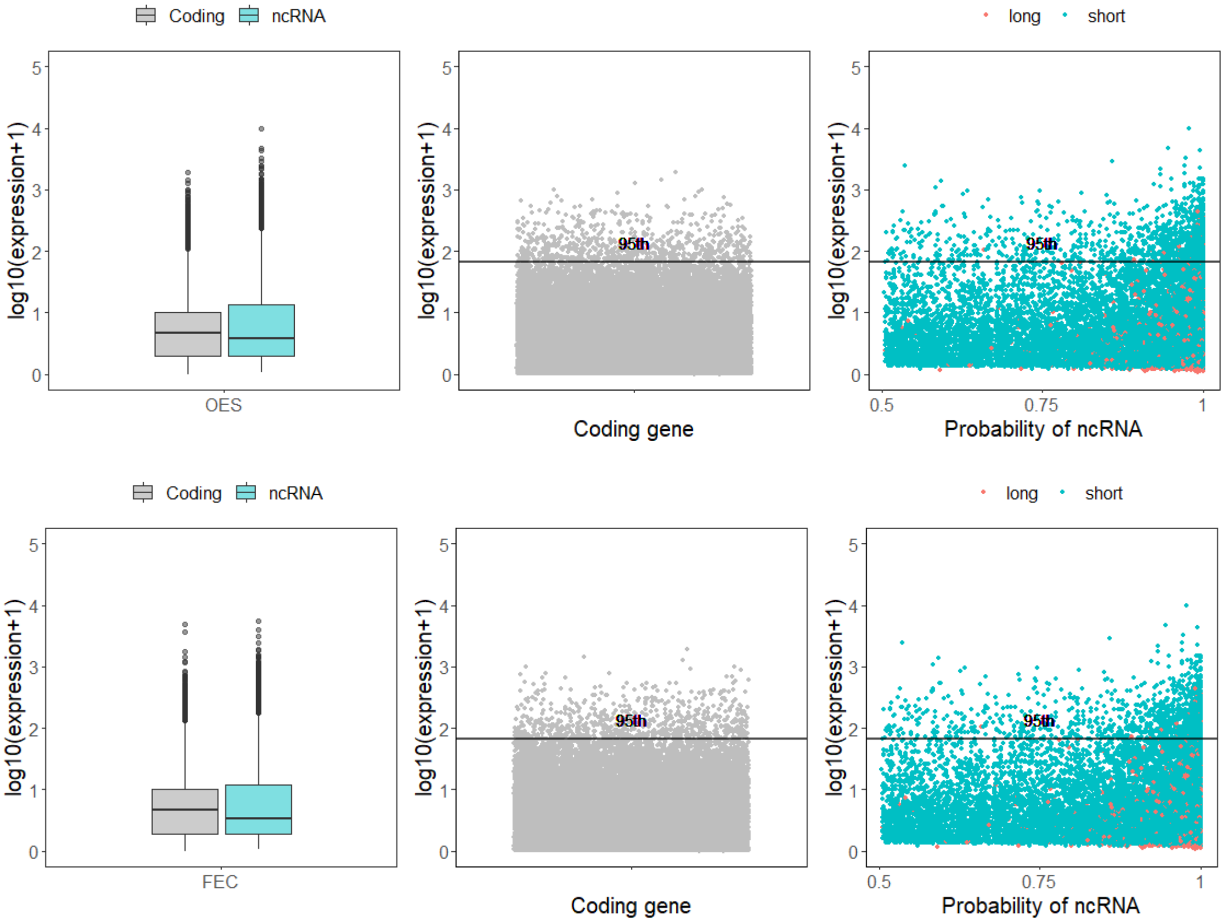

Figure S4: Comparison of expression level between unmatched ncRNAs with known ncRNAs and protein coding genes in cassava RNA-seq data from Wilson [39]. Y-axis represents expression level with normalization by GeTMM. The left graph represents boxplot of expression level in coding genes and unmatched ncRNAs. The middle graph represents scatterplot of expression distribution in coding genes. The right graph represents scatterplot of expression distribution in short and long unmatched ncRNAs, respectively. X-axis in the right graph determined the confidence (probability to be ncRNA) of unmatched ncRNA according to RNAz tool. Black line at y-intercept denotes 95th percentile rank of expression (continue).

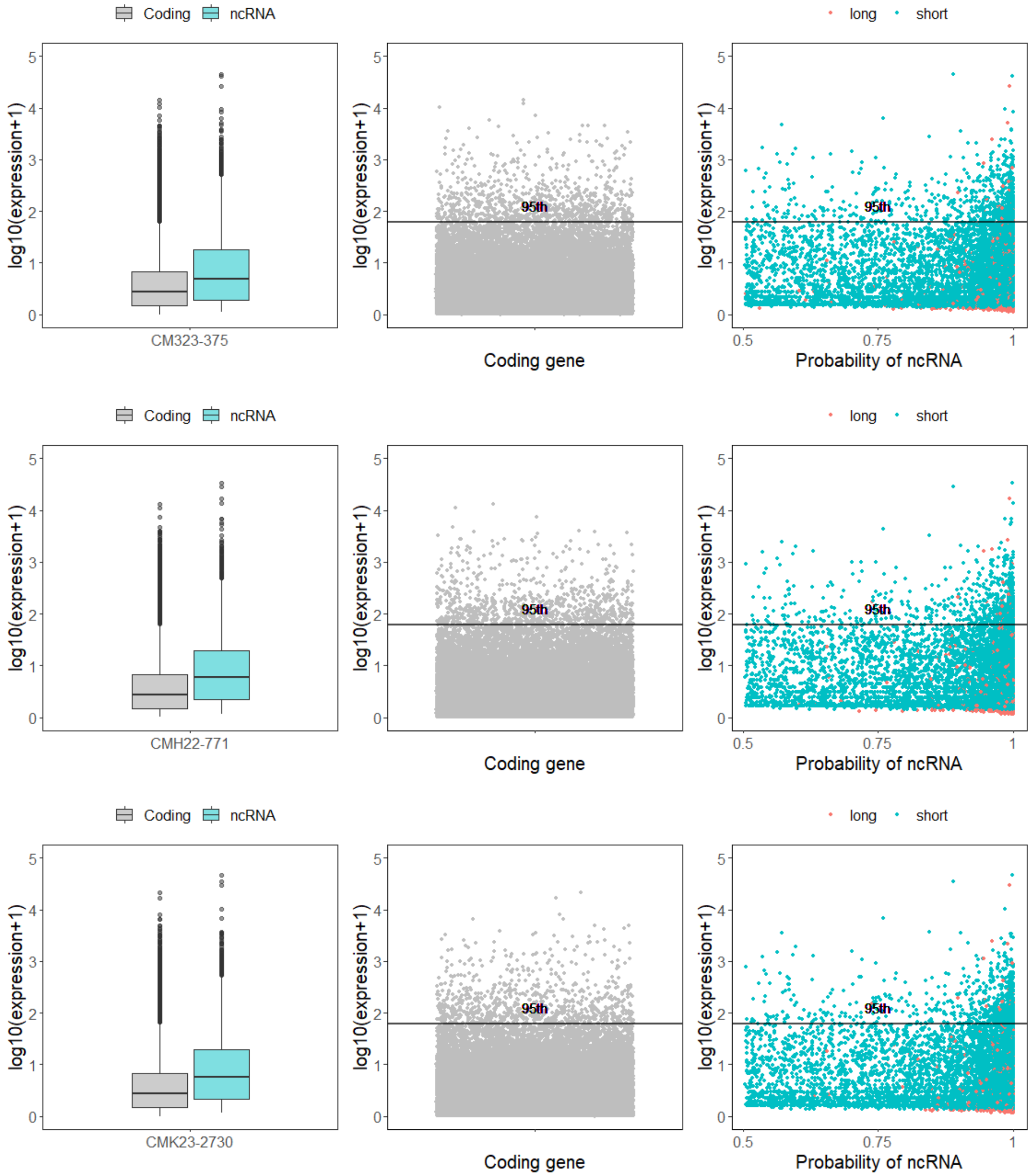

Figure S5: Comparison of expression level between unmatched ncRNAs with known ncRNAs and protein coding genes in cassava RNA-seq data from Pootakham [41]. Y-axis represents expression level with normalization by GeTMM. The left graph represents boxplot of expression level in coding genes and unmatched ncRNAs. The middle graph represents scatterplot of expression distribution in coding genes. The right graph represents scatterplot of expression distribution in short and long unmatched ncRNAs, respectively. X-axis in the right graph determined the confidence (probability to be ncRNA) of unmatched ncRNA according to RNAz tool. Black line at y-intercept denotes 95th percentile rank of expression.

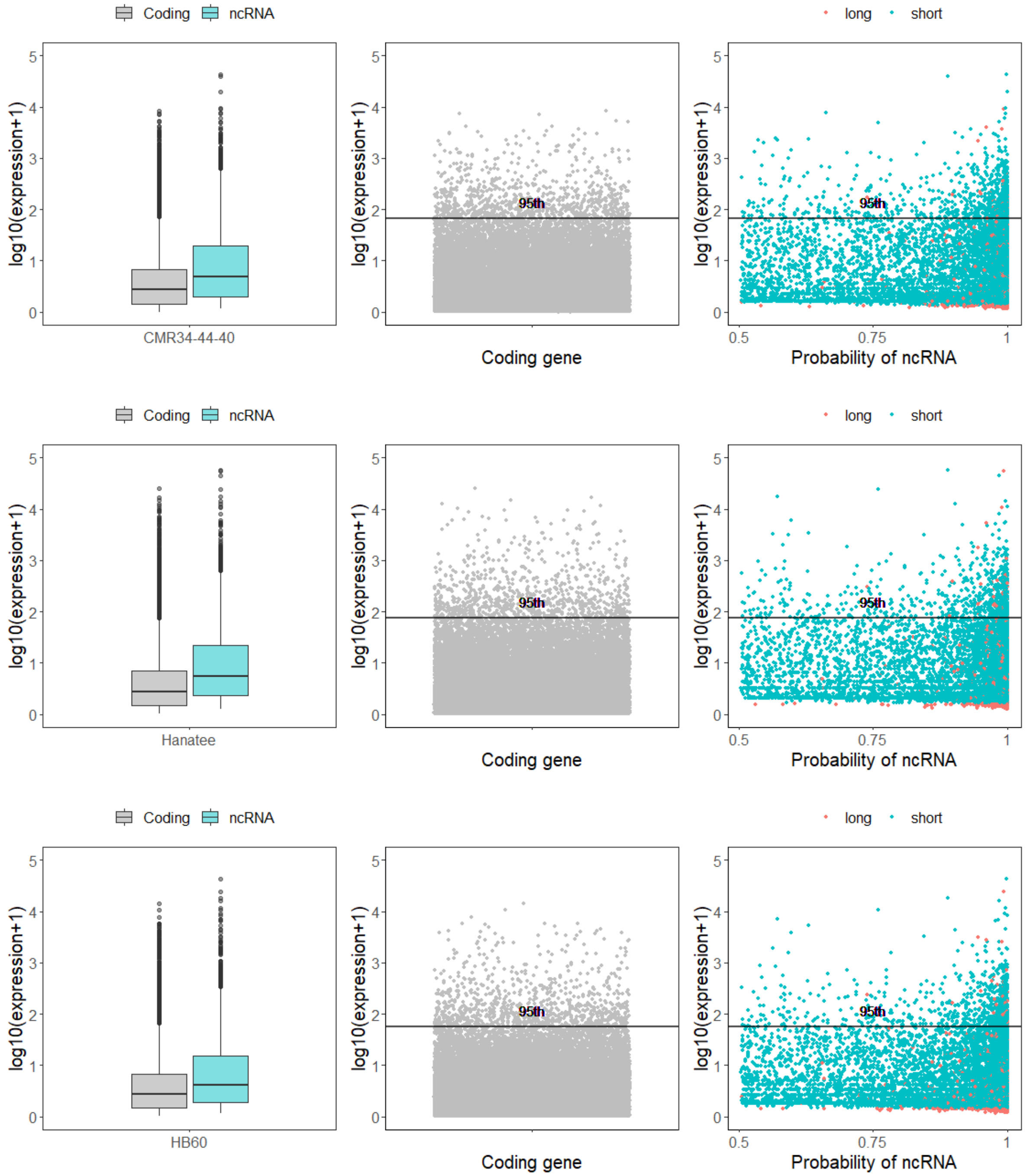

Figure S5: Comparison of expression level between unmatched ncRNAs with known ncRNAs and protein coding genes in cassava RNA-seq data from Pootakham [41]. Y-axis represents expression level with normalization by GeTMM. The left graph represents boxplot of expression level in coding genes and unmatched ncRNAs. The middle graph represents scatterplot of expression distribution in coding genes. The right graph represents scatterplot of expression distribution in short and long unmatched ncRNAs, respectively. X-axis in the right graph determined the confidence (probability to be ncRNA) of unmatched ncRNA according to RNAz tool. Black line at y-intercept denotes 95th percentile rank of expression (continue).

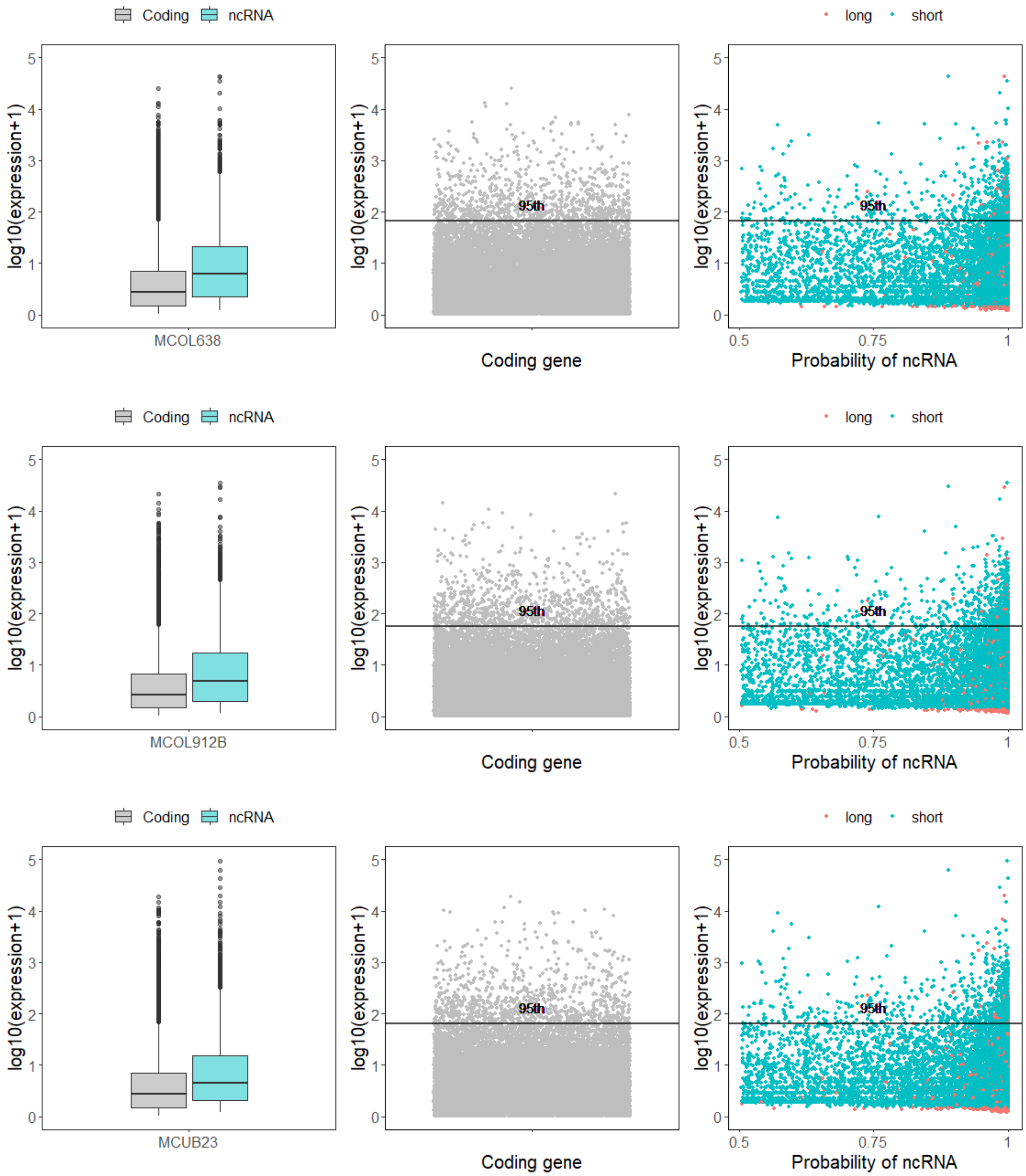

Figure S5: Comparison of expression level between unmatched ncRNAs with known ncRNAs and protein coding genes in cassava RNA-seq data from Pootakham [41]. Y-axis represents expression level with normalization by GeTMM. The left graph represents boxplot of expression level in coding genes and unmatched ncRNAs. The middle graph represents scatterplot of expression distribution in coding genes. The right graph represents scatterplot of expression distribution in short and long unmatched ncRNAs, respectively. X-axis in the right graph determined the confidence (probability to be ncRNA) of unmatched ncRNA according to RNAz tool. Black line at y-intercept denotes 95th percentile rank of expression (continue).

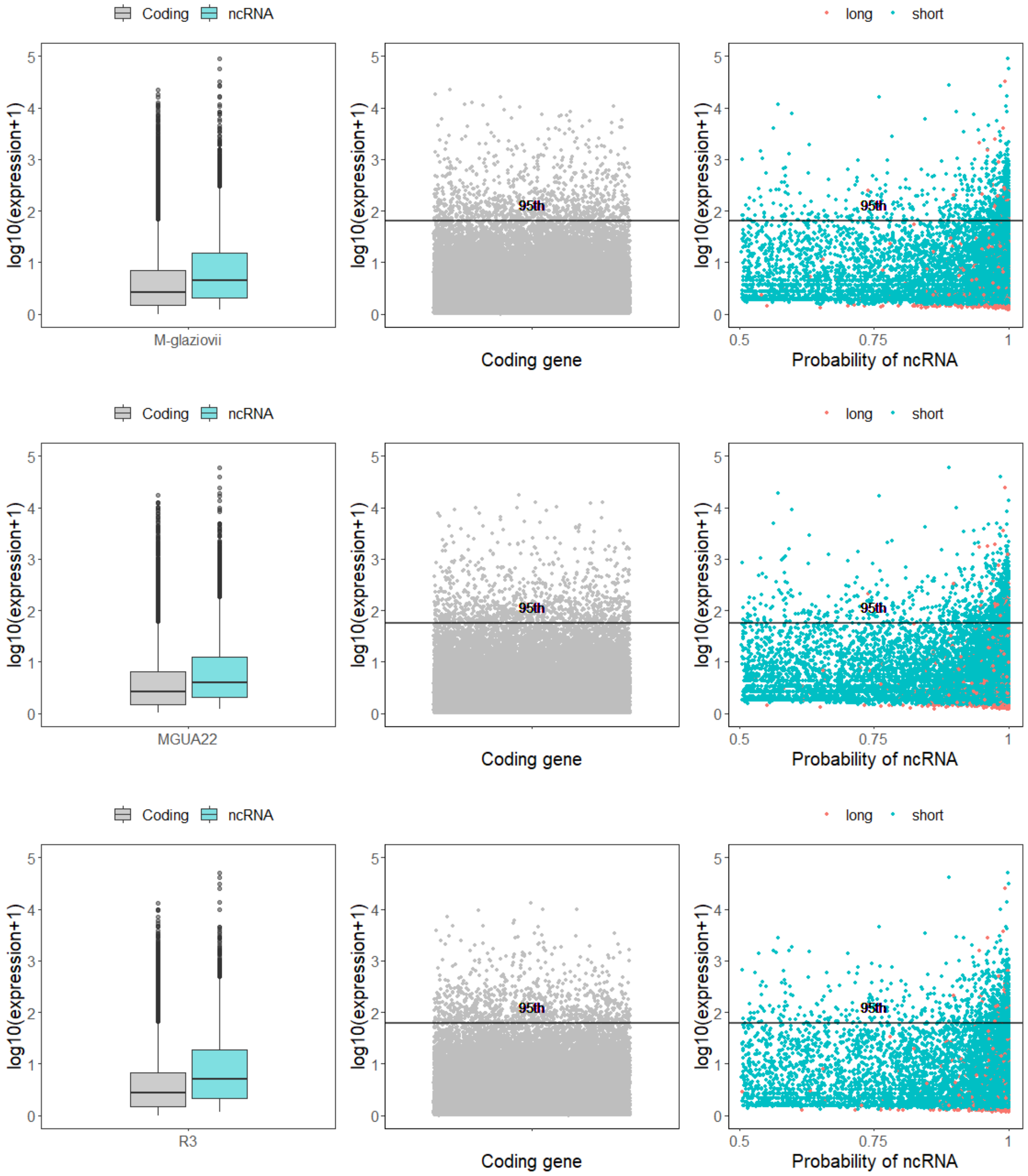

Figure S5: Comparison of expression level between unmatched ncRNAs with known ncRNAs and protein coding genes in cassava RNA-seq data from Pootakham [41]. Y-axis represents expression level with normalization by GeTMM. The left graph represents boxplot of expression level in coding genes and unmatched ncRNAs. The middle graph represents scatterplot of expression distribution in coding genes. The right graph represents scatterplot of expression distribution in short and long unmatched ncRNAs, respectively. X-axis in the right graph determined the confidence (probability to be ncRNA) of unmatched ncRNA according to RNAz tool. Black line at y-intercept denotes 95th percentile rank of expression (continue).

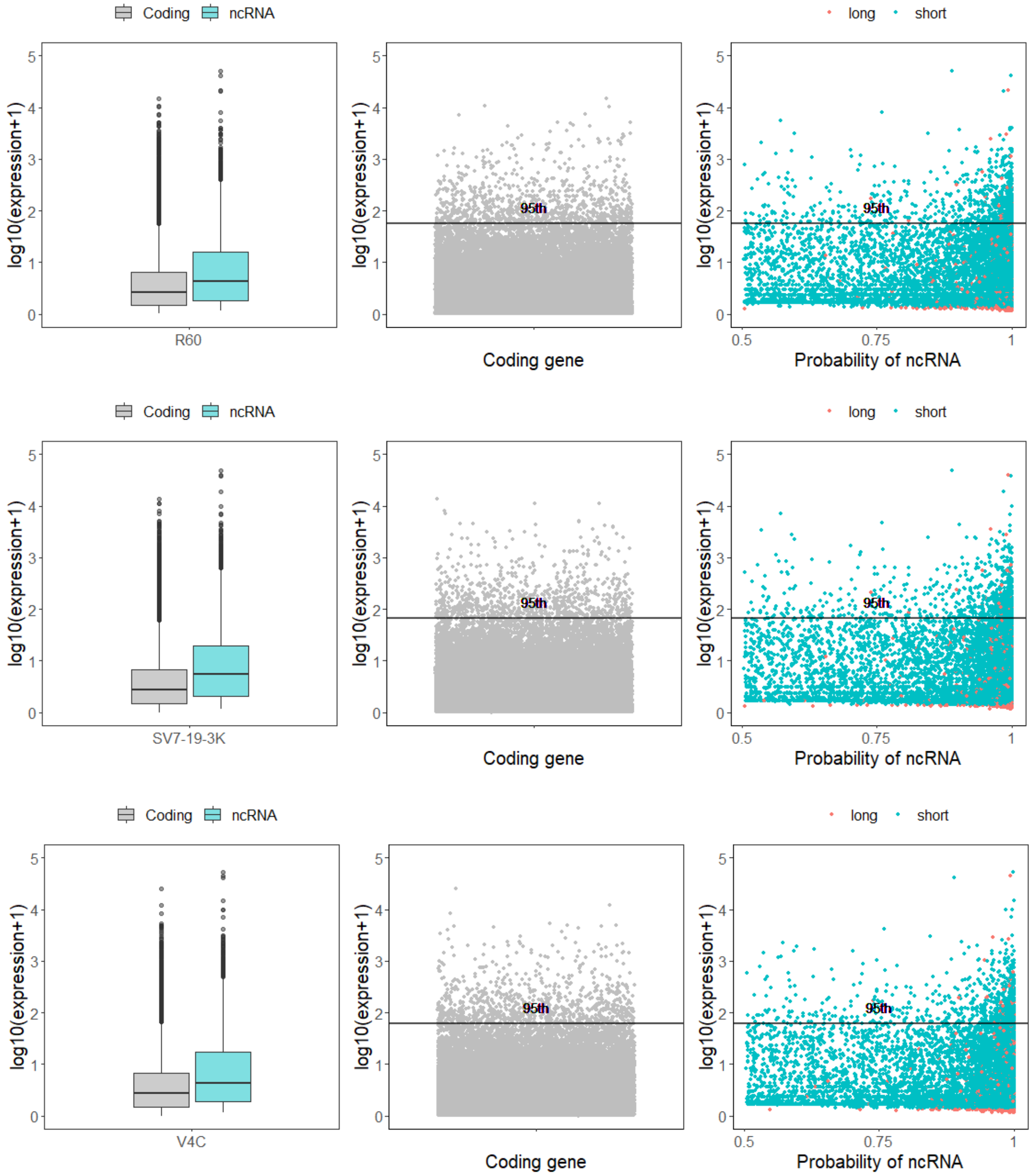

Figure S5: Comparison of expression level between unmatched ncRNAs with known ncRNAs and protein coding genes in cassava RNA-seq data from Pootakham [41]. Y-axis represents expression level with normalization by GeTMM. The left graph represents boxplot of expression level in coding genes and unmatched ncRNAs. The middle graph represents scatterplot of expression distribution in coding genes. The right graph represents scatterplot of expression distribution in short and long unmatched ncRNAs, respectively. X-axis in the right graph determined the confidence (probability to be ncRNA) of unmatched ncRNA according to RNAz tool. Black line at y-intercept denotes 95th percentile rank of expression (continue).

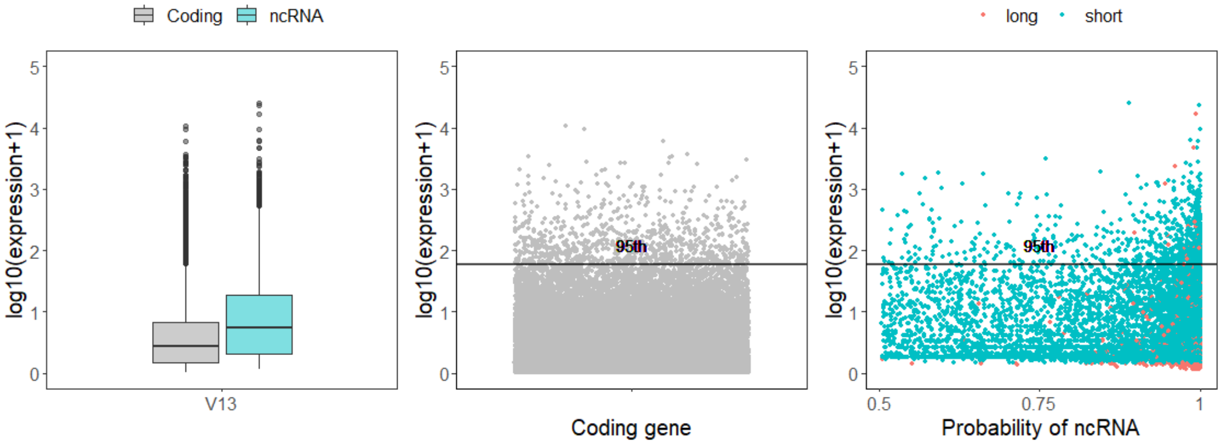

Figure S5: Comparison of expression level between unmatched ncRNAs with known ncRNAs and protein coding genes in cassava RNA-seq data from Pootakham [41]. Y-axis represents expression level with normalization by GeTMM. The left graph represents boxplot of expression level in coding genes and unmatched ncRNAs. The middle graph represents scatterplot of expression distribution in coding genes. The right graph represents scatterplot of expression distribution in short and long unmatched ncRNAs, respectively. X-axis in the right graph determined the confidence (probability to be ncRNA) of unmatched ncRNA according to RNAz tool. Black line at y-intercept denotes 95th percentile rank of expression (continue).

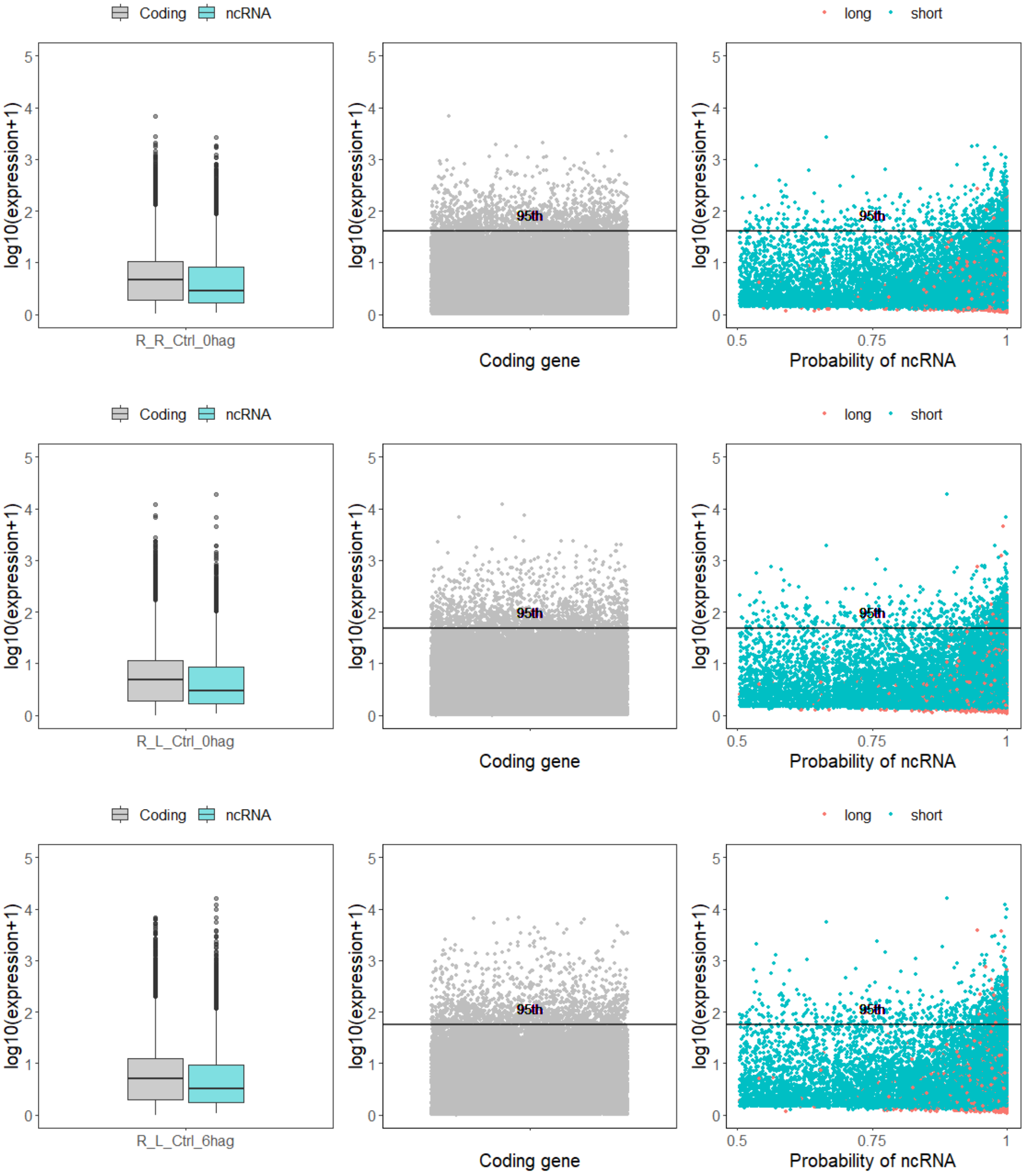

Figure S6: Comparison of expression level between unmatched ncRNAs with known ncRNAs and protein coding genes in CBSV-resistant cassava RNA-seq data from Amuge [40]. Y-axis represents expression level with normalization by GeTMM. The left graph represents boxplot of expression level in coding genes and unmatched ncRNAs. The middle graph represents scatterplot of expression distribution in coding genes. The right graph represents scatterplot of expression distribution in short and long unmatched ncRNAs, respectively. X-axis in the right graph determined the confidence (probability to be ncRNA) of unmatched ncRNA according to RNAz tool. Black line at y-intercept denotes 95th percentile rank of expression.

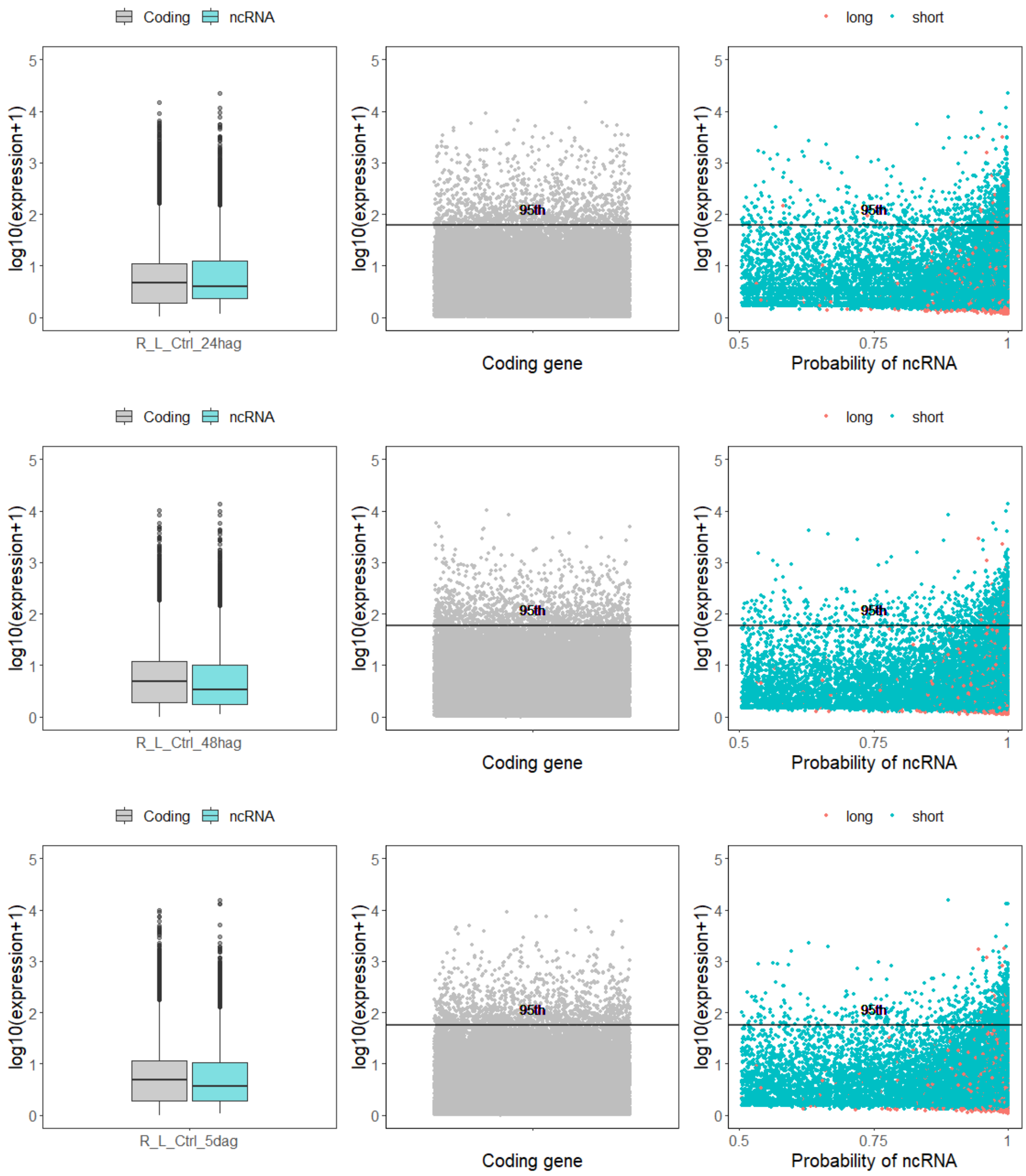

Figure S6: Comparison of expression level between unmatched ncRNAs with known ncRNAs and protein coding genes in CBSV-resistant cassava RNA-seq data from Amuge [40]. Y-axis represents expression level with normalization by GeTMM. The left graph represents boxplot of expression level in coding genes and unmatched ncRNAs. The middle graph represents scatterplot of expression distribution in coding genes. The right graph represents scatterplot of expression distribution in short and long unmatched ncRNAs, respectively. X-axis in the right graph determined the confidence (probability to be ncRNA) of unmatched ncRNA according to RNAz tool. Black line at y-intercept denotes 95th percentile rank of expression (continue).

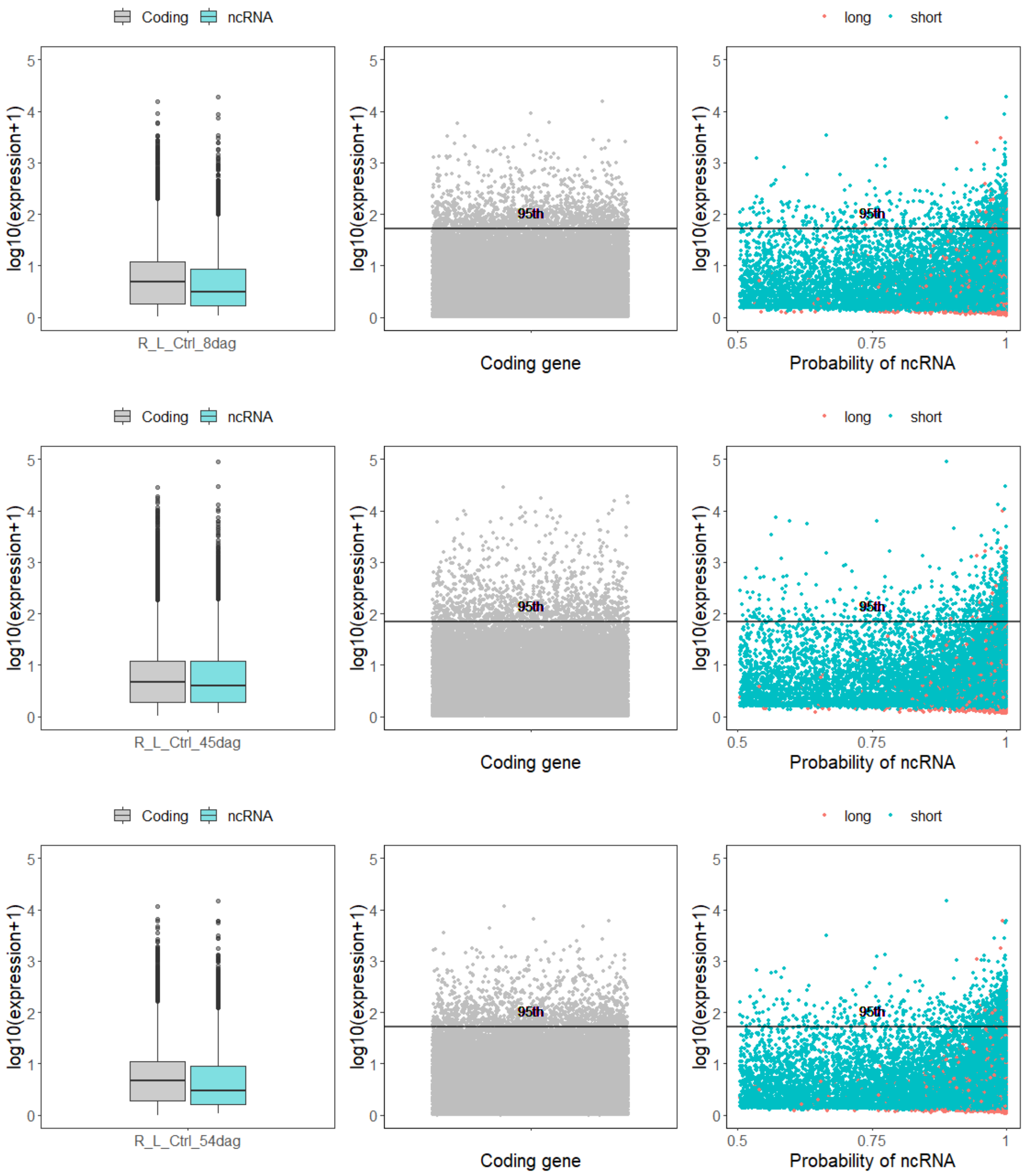

Figure S6: Comparison of expression level between unmatched ncRNAs with known ncRNAs and protein coding genes in CBSV-resistant cassava RNA-seq data from Amuge [40]. Y-axis represents expression level with normalization by GeTMM. The left graph represents boxplot of expression level in coding genes and unmatched ncRNAs. The middle graph represents scatterplot of expression distribution in coding genes. The right graph represents scatterplot of expression distribution in short and long unmatched ncRNAs, respectively. X-axis in the right graph determined the confidence (probability to be ncRNA) of unmatched ncRNA according to RNAz tool. Black line at y-intercept denotes 95th percentile rank of expression (continue).

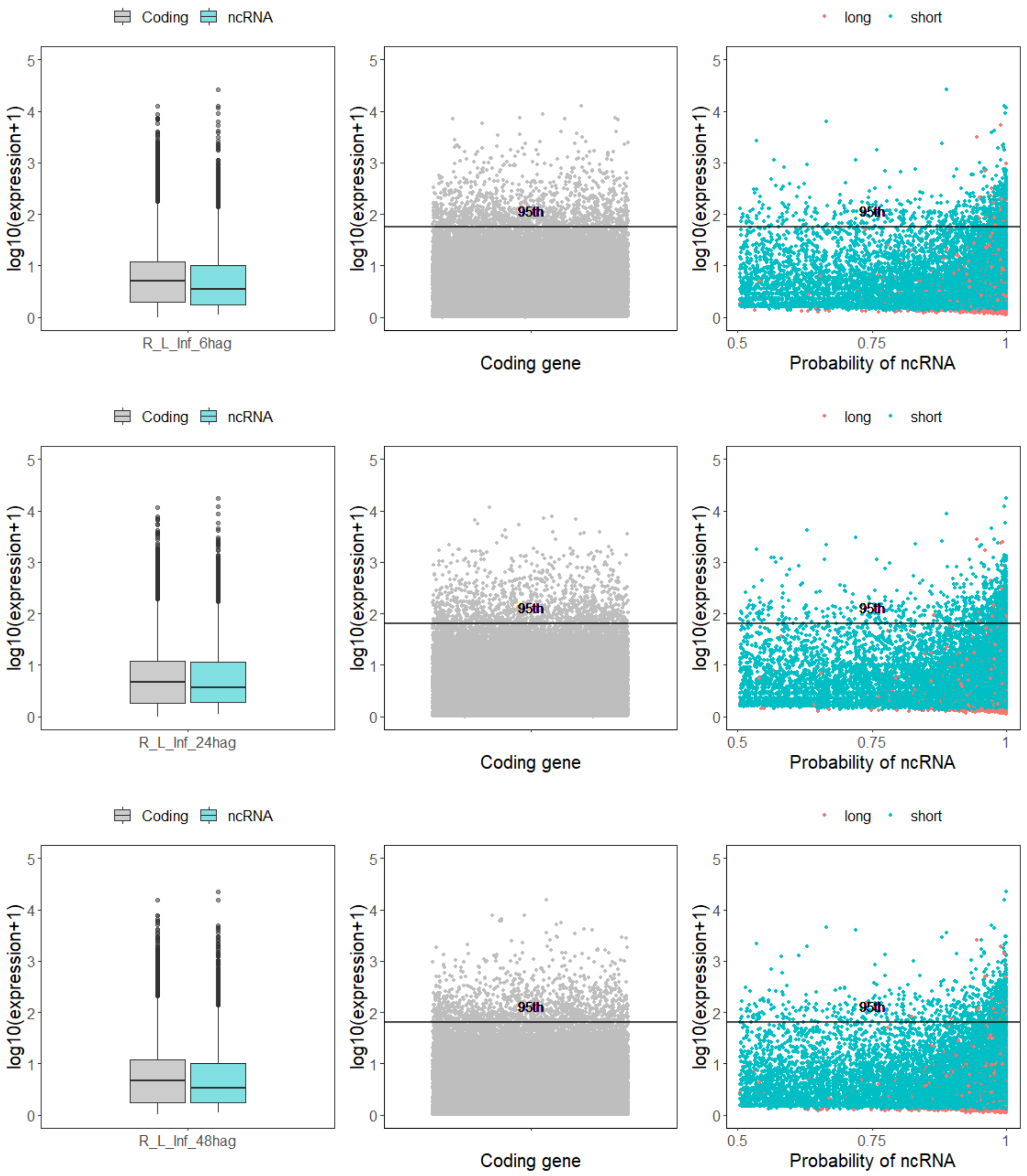

Figure S6: Comparison of expression level between unmatched ncRNAs with known ncRNAs and protein coding genes in CBSV-resistant cassava RNA-seq data from Amuge [40]. Y-axis represents expression level with normalization by GeTMM. The left graph represents boxplot of expression level in coding genes and unmatched ncRNAs. The middle graph represents scatterplot of expression distribution in coding genes. The right graph represents scatterplot of expression distribution in short and long unmatched ncRNAs, respectively. X-axis in the right graph determined the confidence (probability to be ncRNA) of unmatched ncRNA according to RNAz tool. Black line at y-intercept denotes 95th percentile rank of expression (continue).

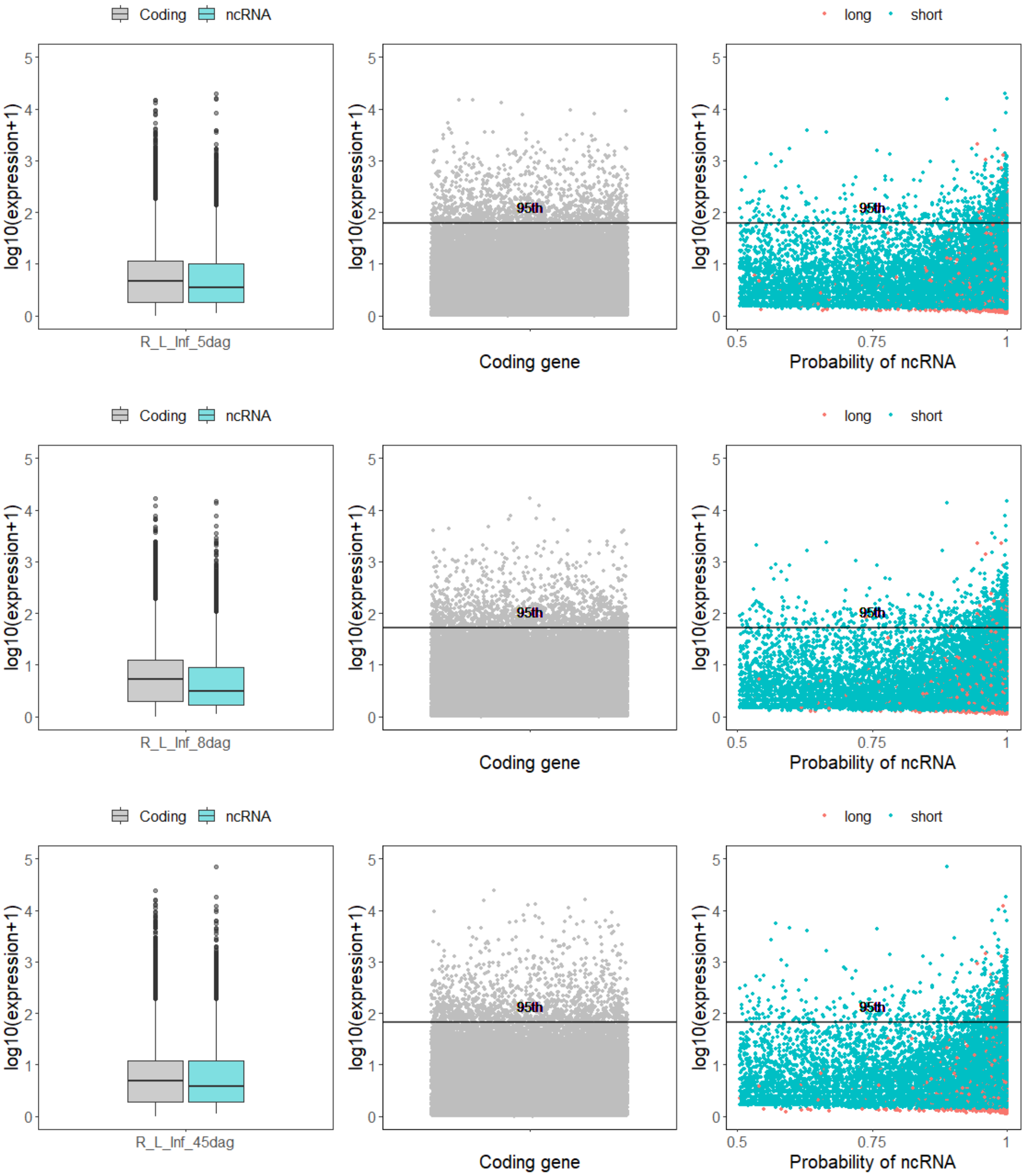

Figure S6: Comparison of expression level between unmatched ncRNAs with known ncRNAs and protein coding genes in CBSV-resistant cassava RNA-seq data from Amuge [40]. Y-axis represents expression level with normalization by GeTMM. The left graph represents boxplot of expression level in coding genes and unmatched ncRNAs. The middle graph represents scatterplot of expression distribution in coding genes. The right graph represents scatterplot of expression distribution in short and long unmatched ncRNAs, respectively. X-axis in the right graph determined the confidence (probability to be ncRNA) of unmatched ncRNA according to RNAz tool. Black line at y-intercept denotes 95th percentile rank of expression (continue).

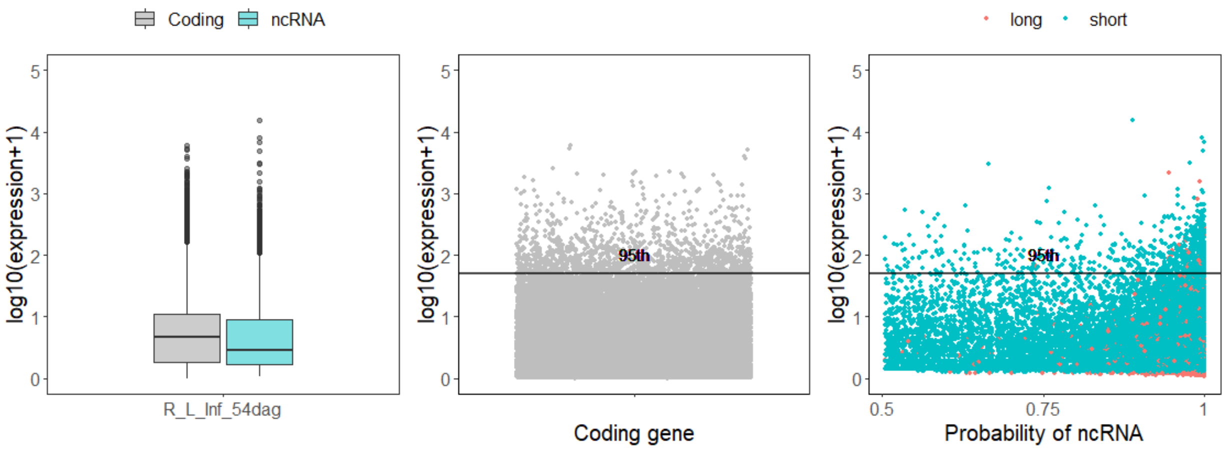

Figure S6: Comparison of expression level between unmatched ncRNAs with known ncRNAs and protein coding genes in CBSV-resistant cassava RNA-seq data from Amuge [40]. Y-axis represents expression level with normalization by GeTMM. The left graph represents boxplot of expression level in coding genes and unmatched ncRNAs. The middle graph represents scatterplot of expression distribution in coding genes. The right graph represents scatterplot of expression distribution in short and long unmatched ncRNAs, respectively. X-axis in the right graph determined the confidence (probability to be ncRNA) of unmatched ncRNA according to RNAz tool. Black line at y-intercept denotes 95th percentile rank of expression (continue).

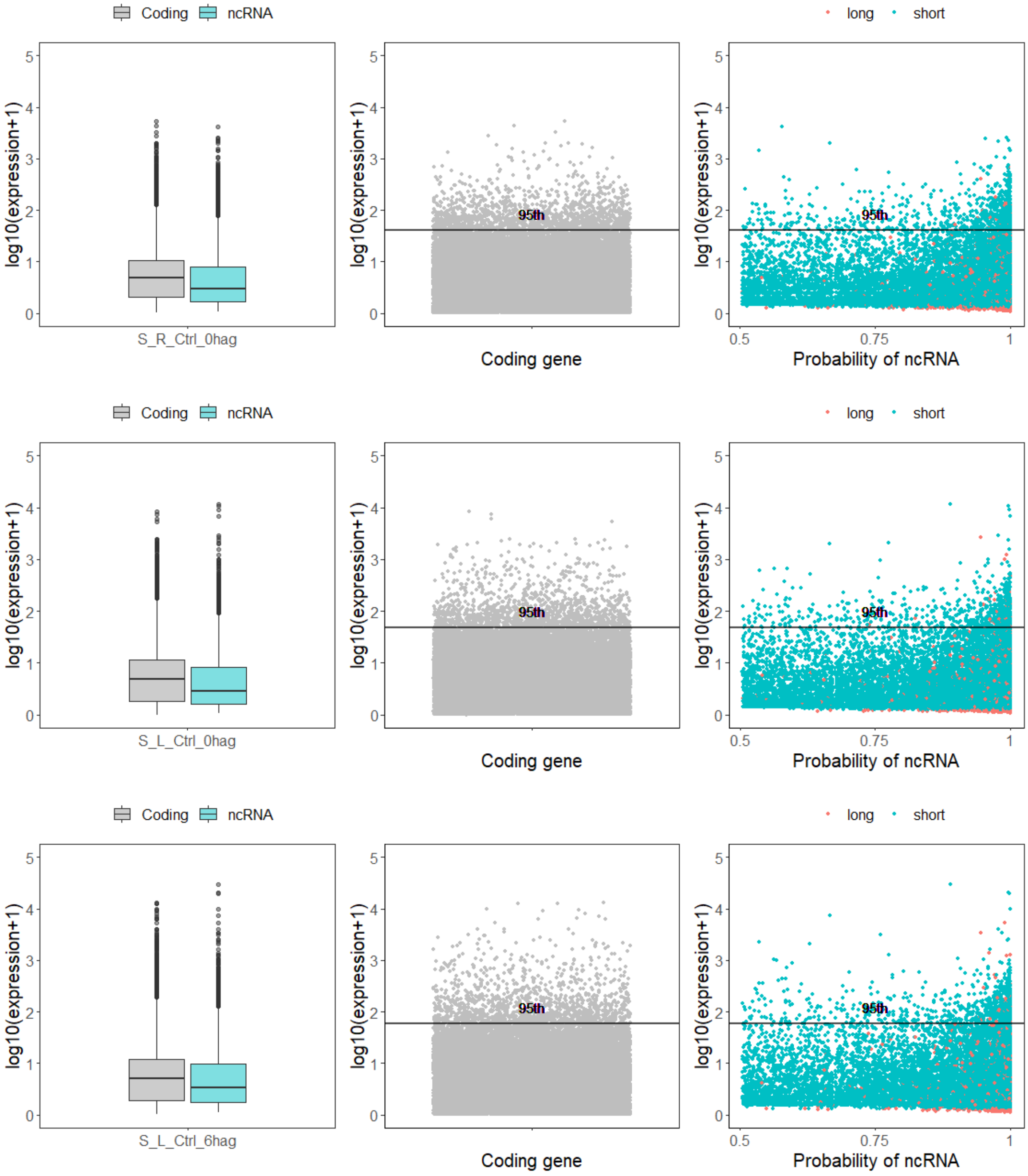

Figure S7: Comparison of expression level between unmatched ncRNAs with known ncRNAs and protein coding genes in CBSV-susceptible cassava RNA-seq data from Amuge [40]. Y-axis represents expression level with normalization by GeTMM. The left graph represents boxplot of expression level in coding genes and unmatched ncRNAs. The middle graph represents scatterplot of expression distribution in coding genes. The right graph represents scatterplot of expression distribution in short and long unmatched ncRNAs, respectively. X-axis in the right graph determined the confidence (probability to be ncRNA) of unmatched ncRNA according to RNAz tool. Black line at y-intercept denotes 95th percentile rank of expression.

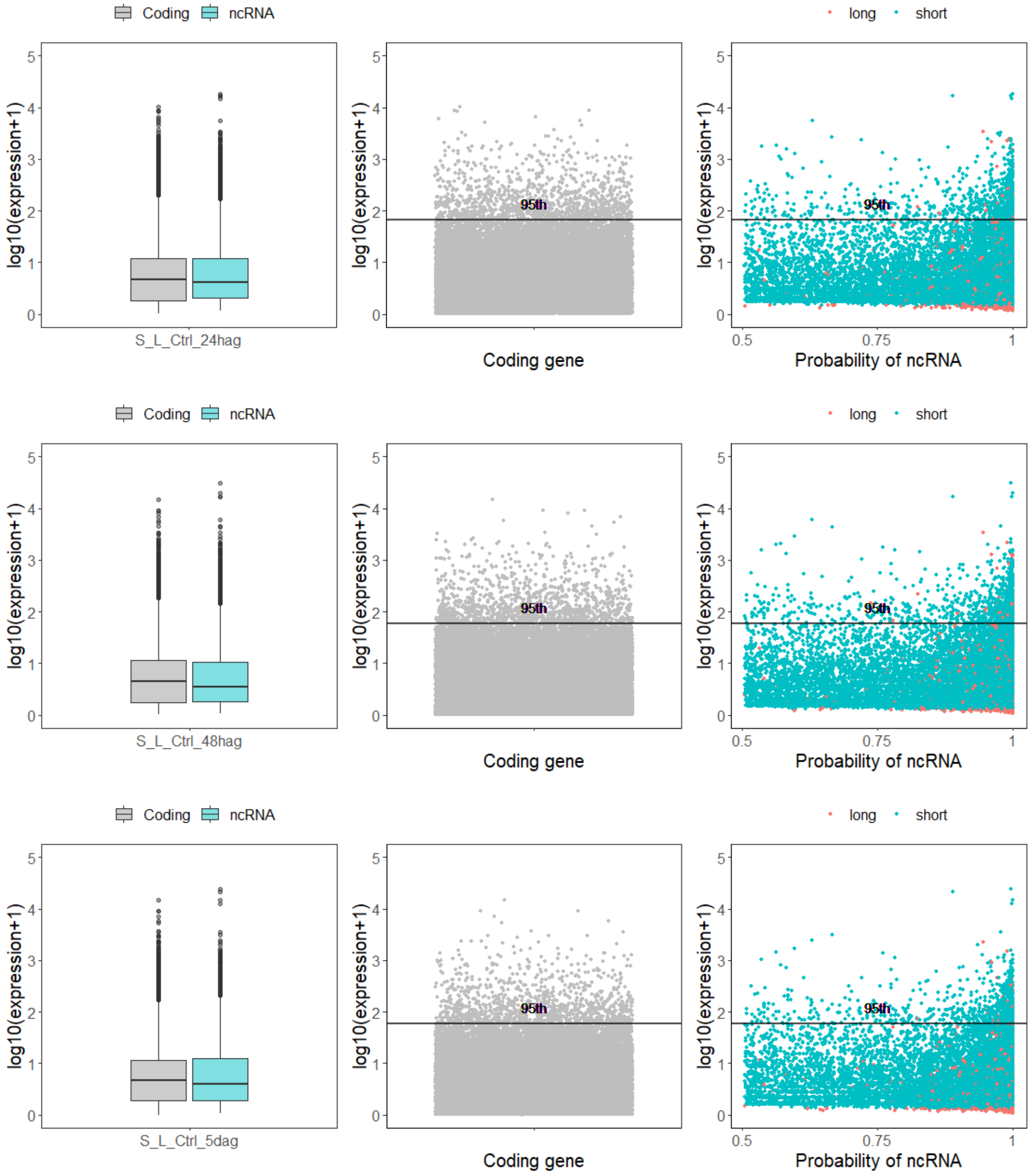

Figure S7: Comparison of expression level between unmatched ncRNAs with known ncRNAs and protein coding genes in CBSV-susceptible cassava RNA-seq data from Amuge [40]. Y-axis represents expression level with normalization by GeTMM. The left graph represents boxplot of expression level in coding genes and unmatched ncRNAs. The middle graph represents scatterplot of expression distribution in coding genes. The right graph represents scatterplot of expression distribution in short and long unmatched ncRNAs, respectively. X-axis in the right graph determined the confidence (probability to be ncRNA) of unmatched ncRNA according to RNAz tool. Black line at y-intercept denotes 95th percentile rank of expression (continue).

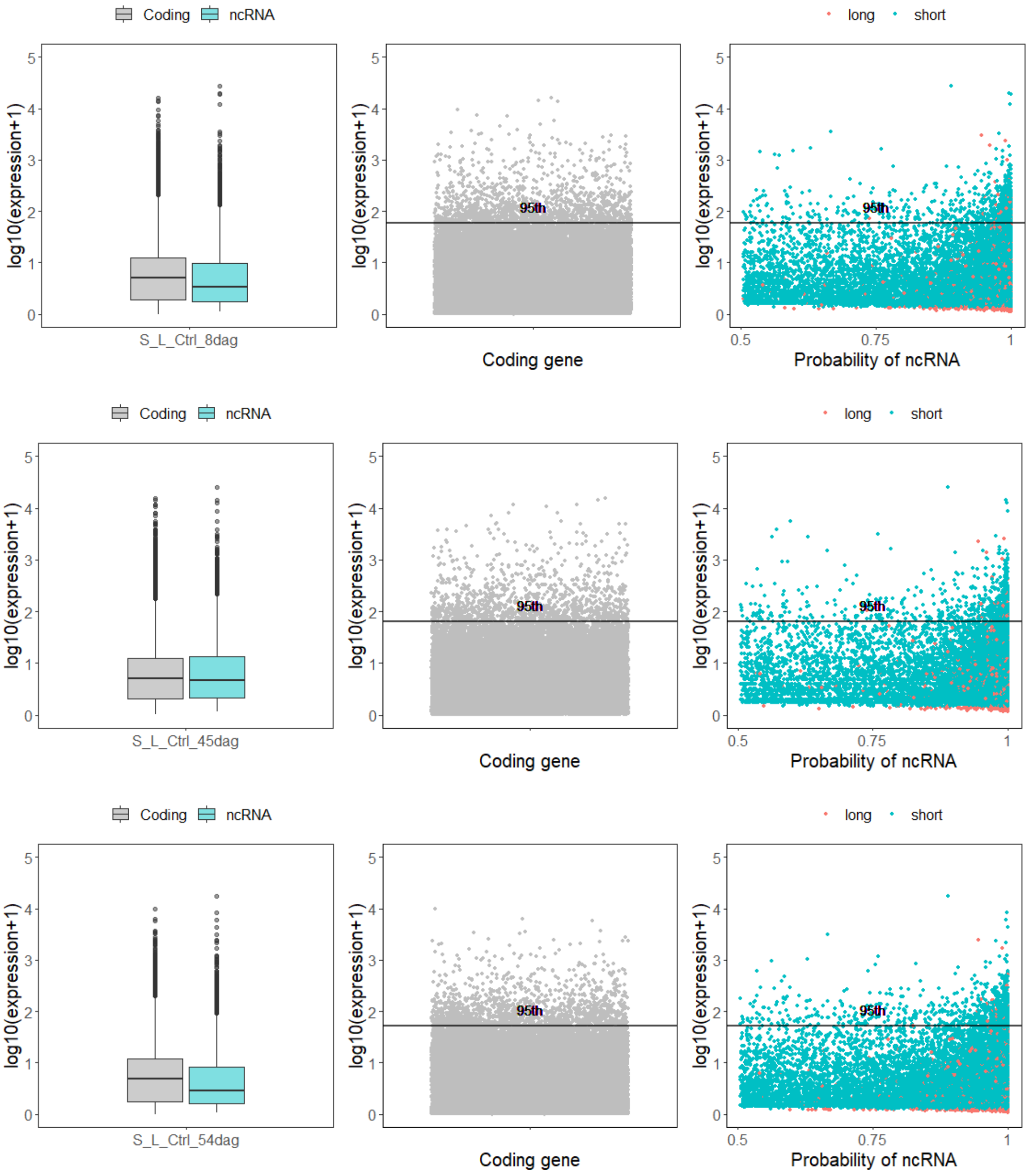

Figure S7: Comparison of expression level between unmatched ncRNAs with known ncRNAs and protein coding genes in CBSV-susceptible cassava RNA-seq data from Amuge [40]. Y-axis represents expression level with normalization by GeTMM. The left graph represents boxplot of expression level in coding genes and unmatched ncRNAs. The middle graph represents scatterplot of expression distribution in coding genes. The right graph represents scatterplot of expression distribution in short and long unmatched ncRNAs, respectively. X-axis in the right graph determined the confidence (probability to be ncRNA) of unmatched ncRNA according to RNAz tool. Black line at y-intercept denotes 95th percentile rank of expression (continue).

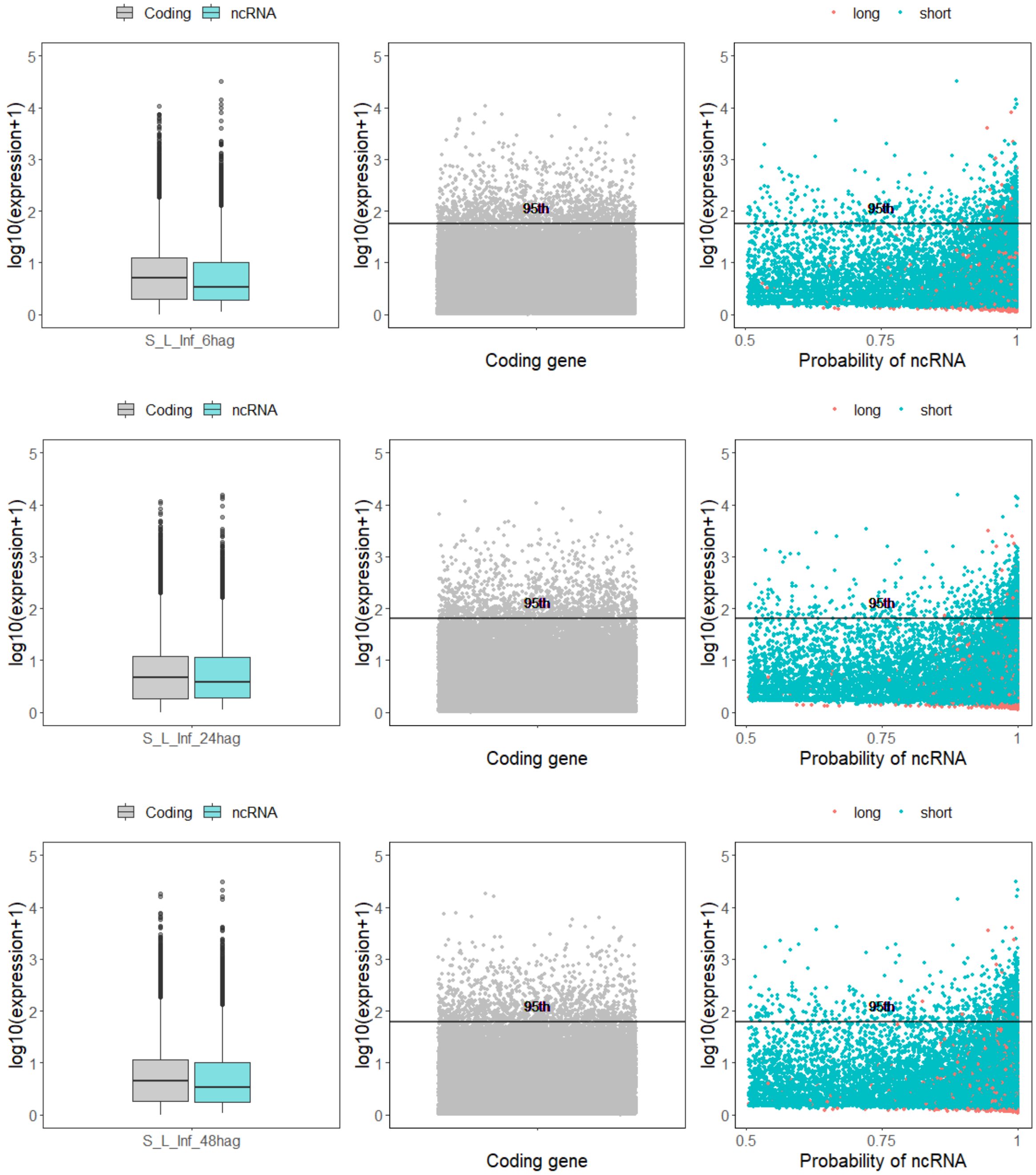

Figure S7: Comparison of expression level between unmatched ncRNAs with known ncRNAs and protein coding genes in CBSV-susceptible cassava RNA-seq data from Amuge [40]. Y-axis represents expression level with normalization by GeTMM. The left graph represents boxplot of expression level in coding genes and unmatched ncRNAs. The middle graph represents scatterplot of expression distribution in coding genes. The right graph represents scatterplot of expression distribution in short and long unmatched ncRNAs, respectively. X-axis in the right graph determined the confidence (probability to be ncRNA) of unmatched ncRNA according to RNAz tool. Black line at y-intercept denotes 95th percentile rank of expression (continue).

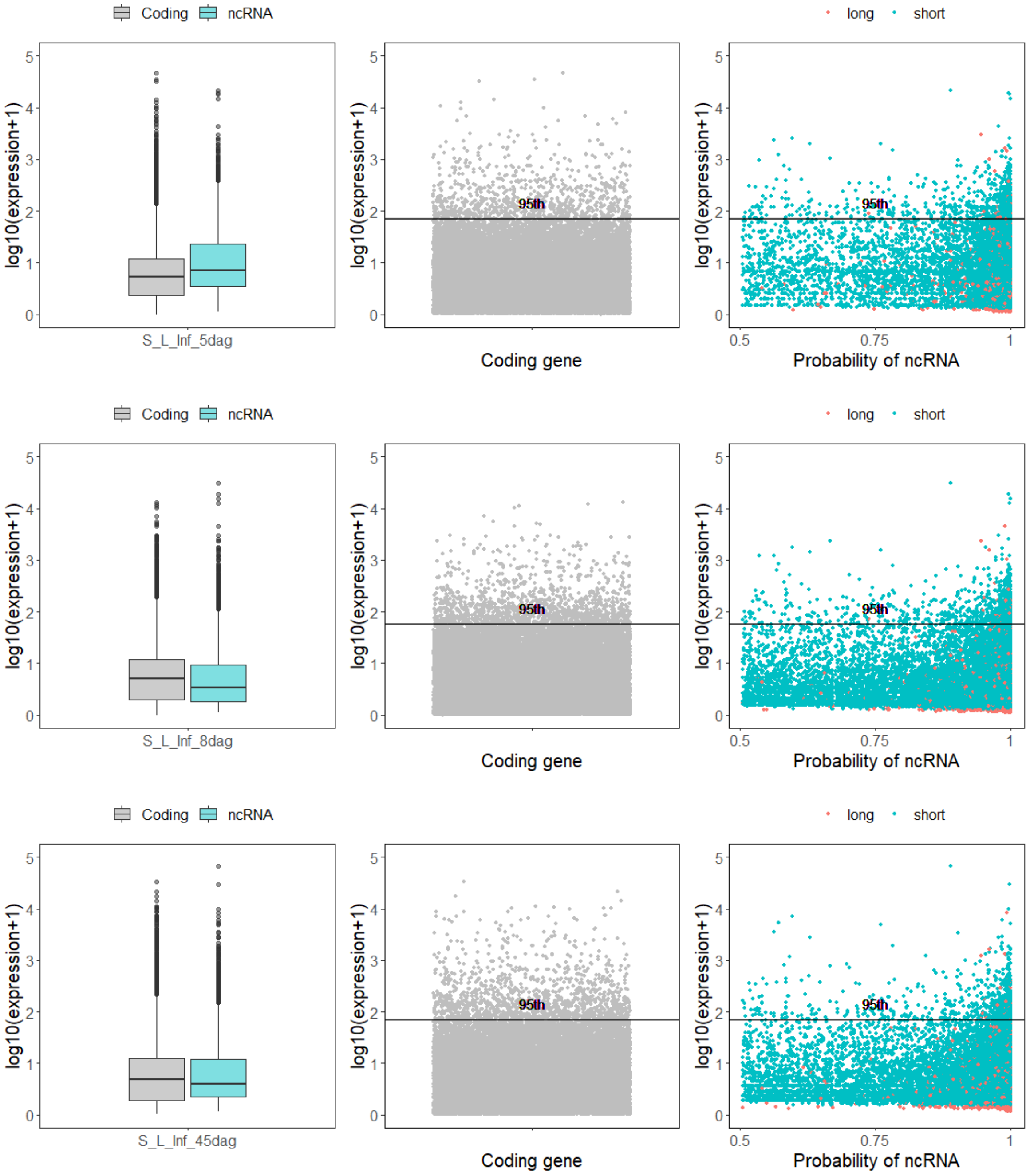

Figure S7: Comparison of expression level between unmatched ncRNAs with known ncRNAs and protein coding genes in CBSV-susceptible cassava RNA-seq data from Amuge [40]. Y-axis represents expression level with normalization by GeTMM. The left graph represents boxplot of expression level in coding genes and unmatched ncRNAs. The middle graph represents scatterplot of expression distribution in coding genes. The right graph represents scatterplot of expression distribution in short and long unmatched ncRNAs, respectively. X-axis in the right graph determined the confidence (probability to be ncRNA) of unmatched ncRNA according to RNAz tool. Black line at y-intercept denotes 95th percentile rank of expression (continue).

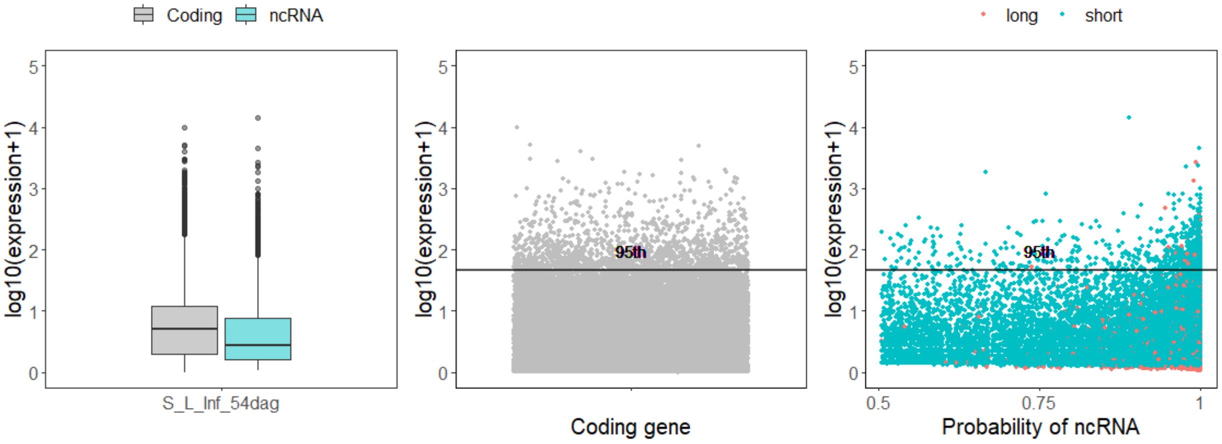

Figure S7: Comparison of expression level between unmatched ncRNAs with known ncRNAs and protein coding genes in CBSV-susceptible cassava RNA-seq data from Amuge [40]. Y-axis represents expression level with normalization by GeTMM. The left graph represents boxplot of expression level in coding genes and unmatched ncRNAs. The middle graph represents scatterplot of expression distribution in coding genes. The right graph represents scatterplot of expression distribution in short and long unmatched ncRNAs, respectively. X-axis in the right graph determined the confidence (probability to be ncRNA) of unmatched ncRNA according to RNAz tool. Black line at y-intercept denotes 95th percentile rank of expression (continue).

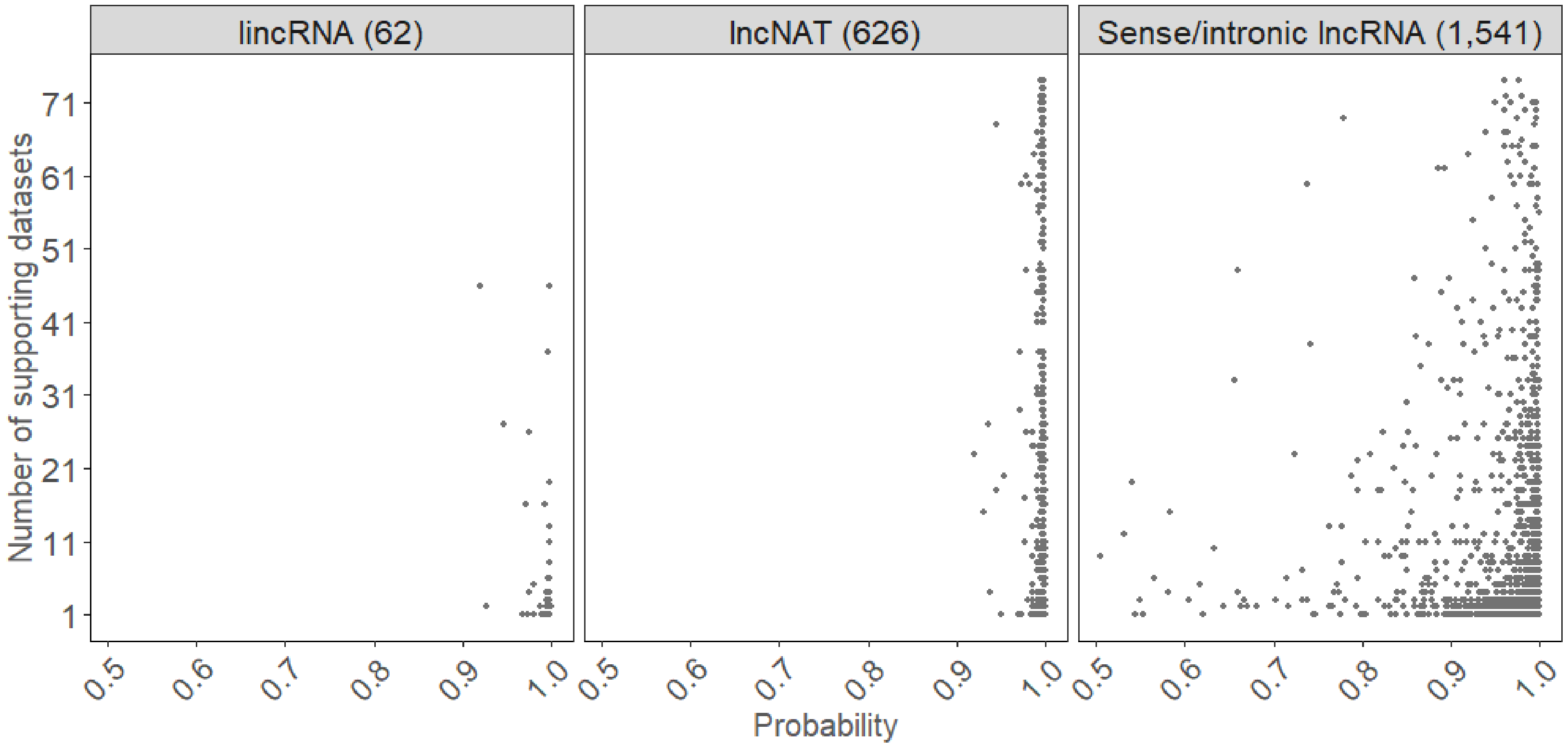

Figure S8: Expression supporting and confidence of potential novel lncRNAs. Scatter plot represents 2,229 novel lncRNAs (Me-lncRNAs) comprise of lincRNAs, lncNAT and sense/intronic-lncRNA. X-axis indicates the confidence (probability to be ncRNA) of putative lncRNA according to RNAz prediction y-axis represents number of supporting datasets.

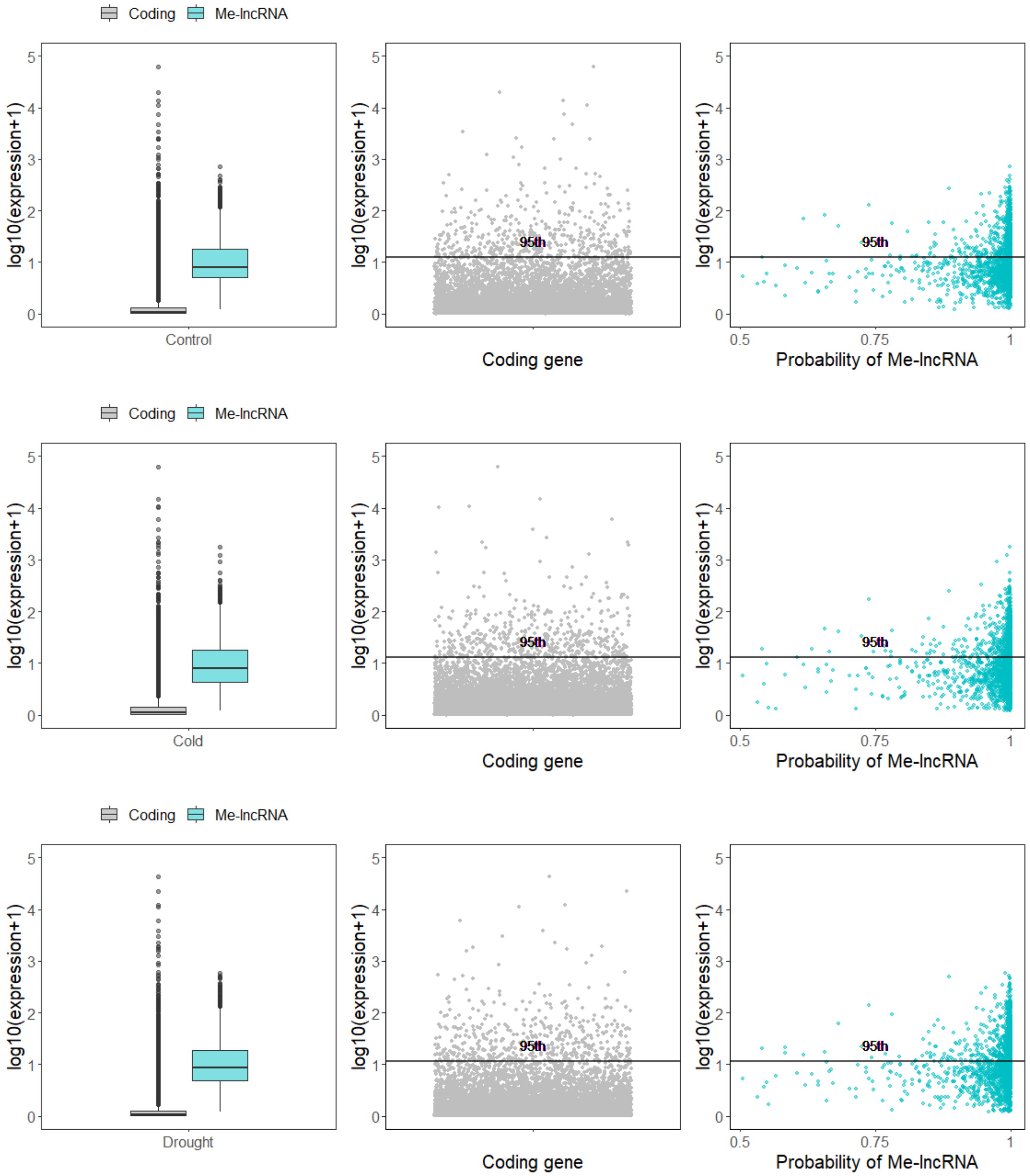

Figure S9: Comparison of expression level between Me-lncRNAs and protein coding genes in cassava RNA-seq data from Li [15]. Y-axis represents expression level with normalization by GeTMM. The left graph represents boxplot of expression level in coding genes and Me-lncRNAs. The middle graph represents scatterplot of expression distribution in coding genes. The right graph represents scatterplot of expression distribution in Me-lncRNAs. X-axis in the right graph determined the confidence (probability to be ncRNA) of Me-lncRNAs according to RNAz tool. Black line at y-intercept denotes 95th percentile rank of expression.

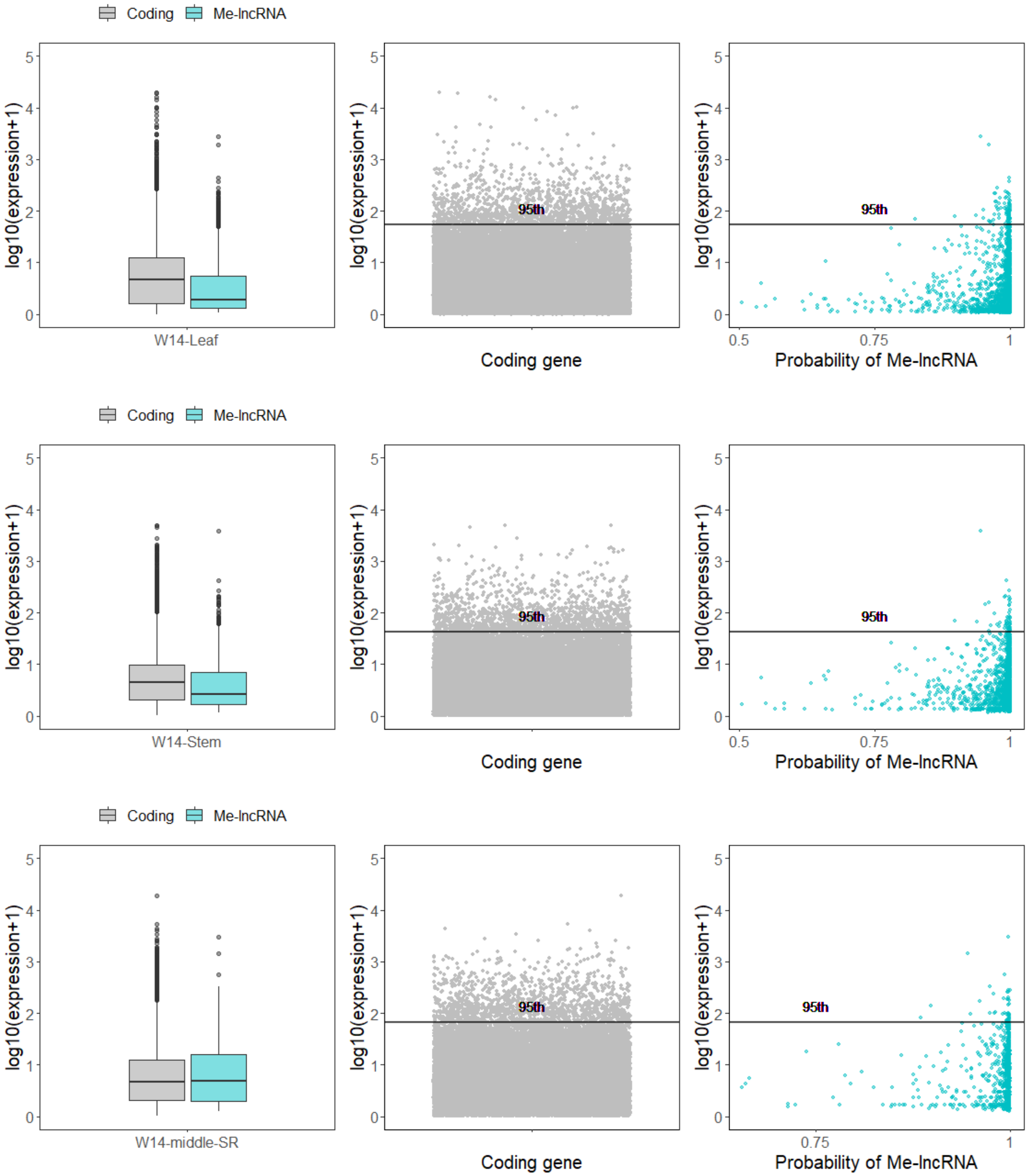

Figure S10: Comparison of expression level between Me-lncRNAs and protein coding genes in cassava RNA-seq data from Wang [32]. Y-axis represents expression level with normalization by GeTMM. The left graph represents boxplot of expression level in coding genes and Me-lncRNAs. The middle graph represents scatterplot of expression distribution in coding genes. The right graph represents scatterplot of expression distribution in Me-lncRNAs. X-axis in the right graph determined the confidence (probability to be ncRNA) of Me-lncRNAs according to RNAz tool. Black line at y-intercept denotes 95th percentile rank of expression.

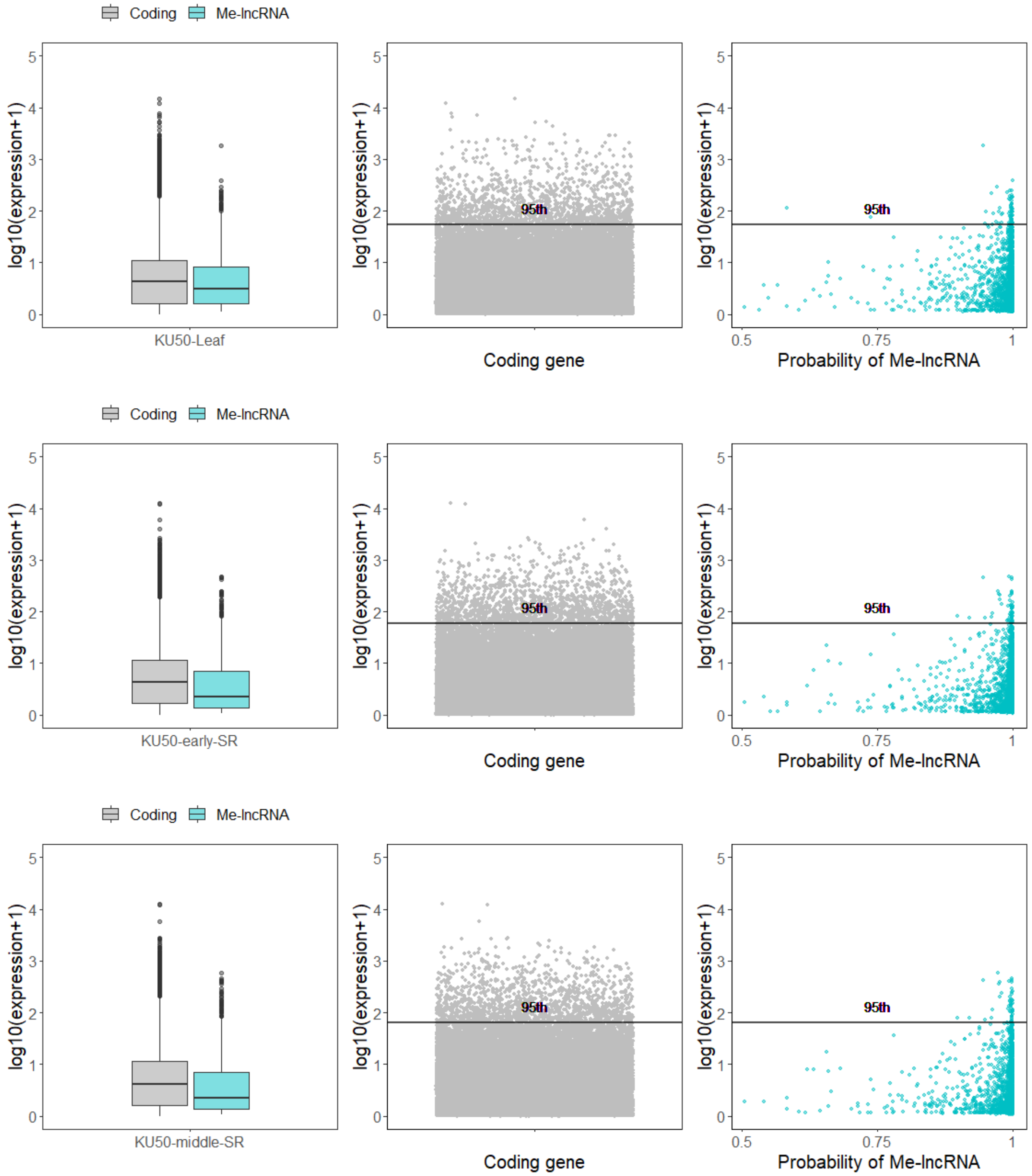

Figure S10: Comparison of expression level between Me-lncRNAs and protein coding genes in cassava RNA-seq data from Wang [32]. Y-axis represents expression level with normalization by GeTMM. The left graph represents boxplot of expression level in coding genes and Me-lncRNAs. The middle graph represents scatterplot of expression distribution in coding genes. The right graph represents scatterplot of expression distribution in Me-lncRNAs. X-axis in the right graph determined the confidence (probability to be ncRNA) of Me-lncRNAs according to RNAz tool. Black line at y-intercept denotes 95th percentile rank of expression (continue).

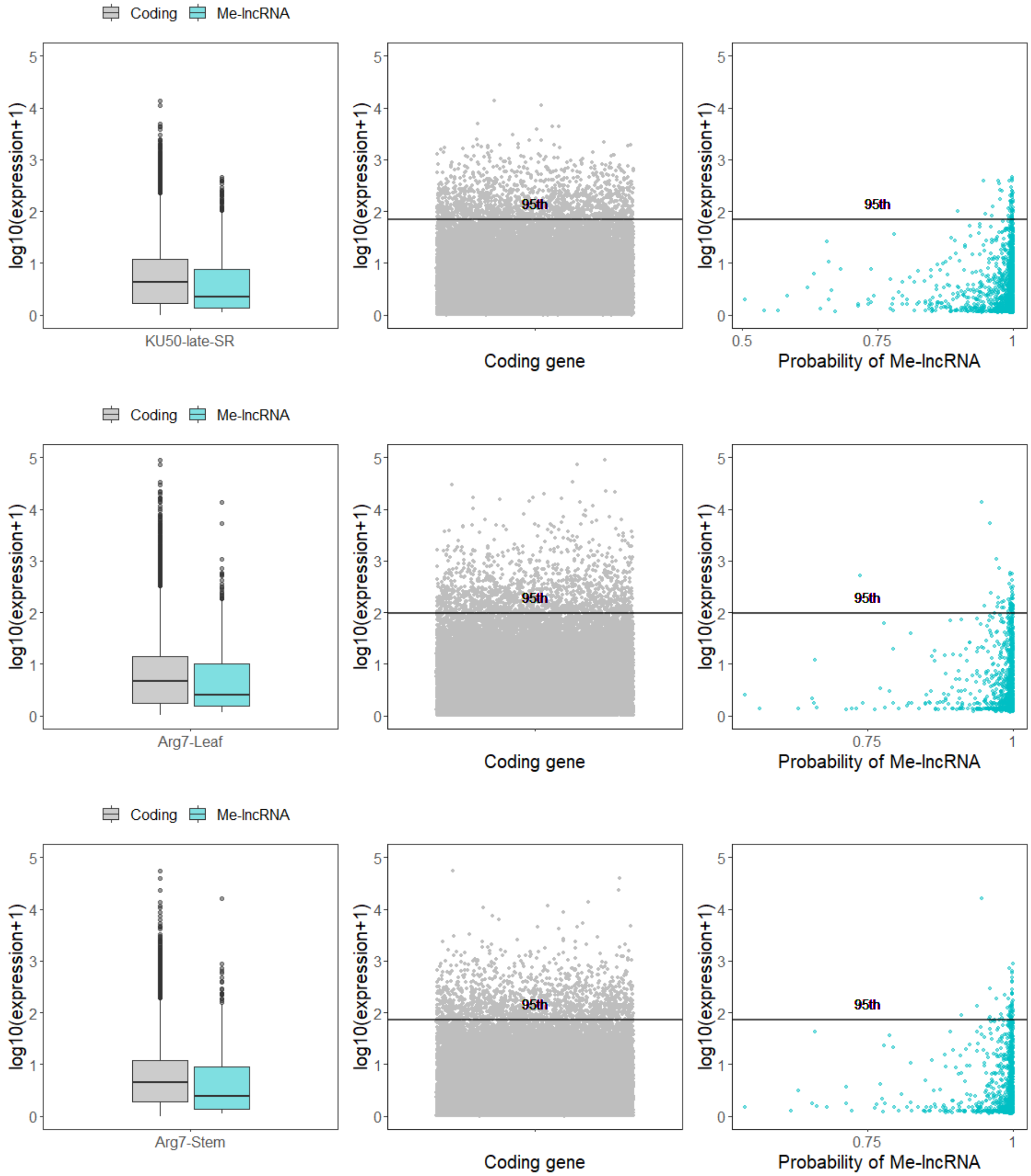

Figure S10: Comparison of expression level between Me-lncRNAs and protein coding genes in cassava RNA-seq data from Wang [32]. Y-axis represents expression level with normalization by GeTMM. The left graph represents boxplot of expression level in coding genes and Me-lncRNAs. The middle graph represents scatterplot of expression distribution in coding genes. The right graph represents scatterplot of expression distribution in Me-lncRNAs. X-axis in the right graph determined the confidence (probability to be ncRNA) of Me-lncRNAs according to RNAz tool. Black line at y-intercept denotes 95th percentile rank of expression (continue).

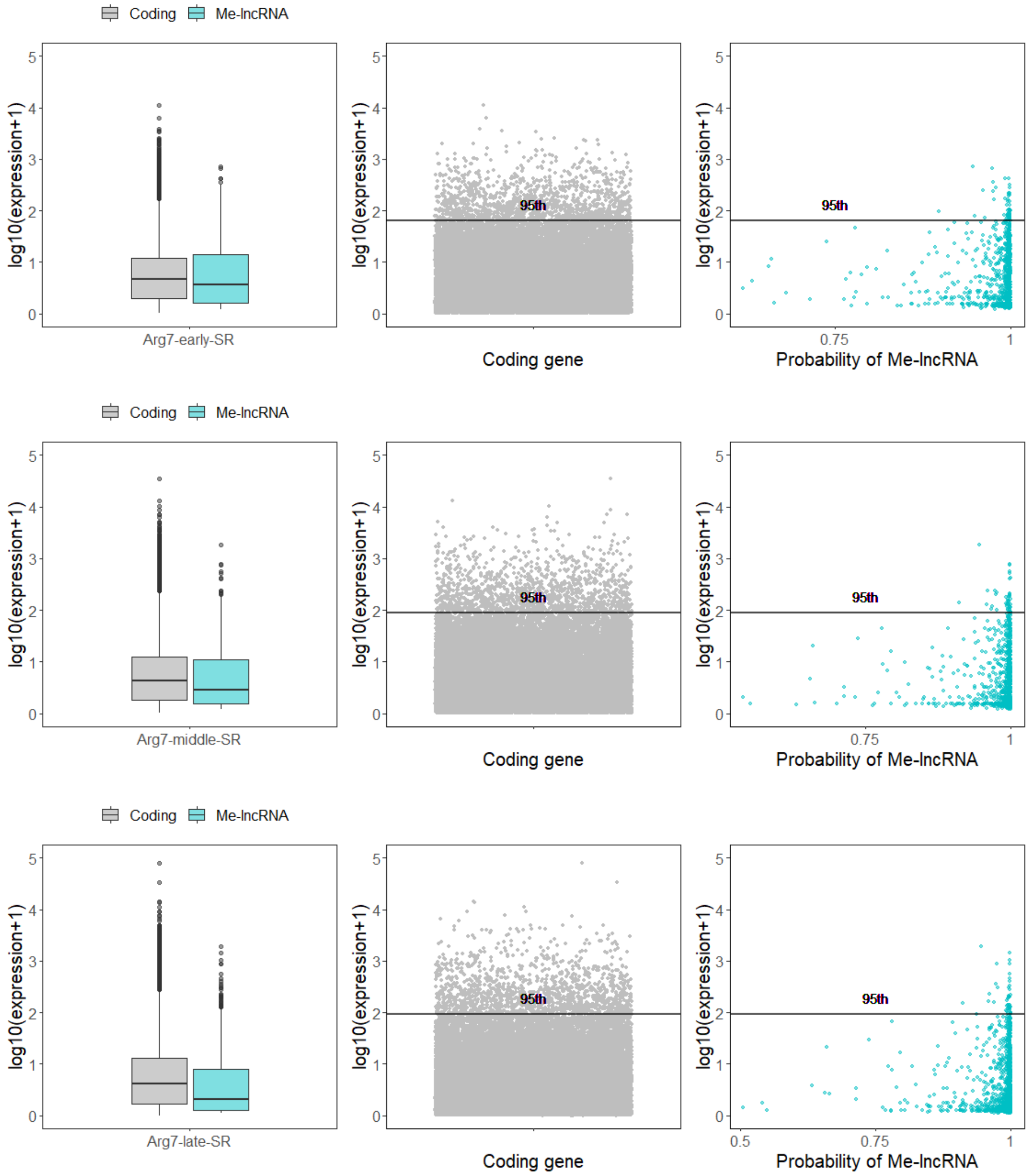

Figure S10: Comparison of expression level between Me-lncRNAs and protein coding genes in cassava RNA-seq data from Wang [32]. Y-axis represents expression level with normalization by GeTMM. The left graph represents boxplot of expression level in coding genes and Me-lncRNAs. The middle graph represents scatterplot of expression distribution in coding genes. The right graph represents scatterplot of expression distribution in Me-lncRNAs. X-axis in the right graph determined the confidence (probability to be ncRNA) of Me-lncRNAs according to RNAz tool. Black line at y-intercept denotes 95th percentile rank of expression (continue).

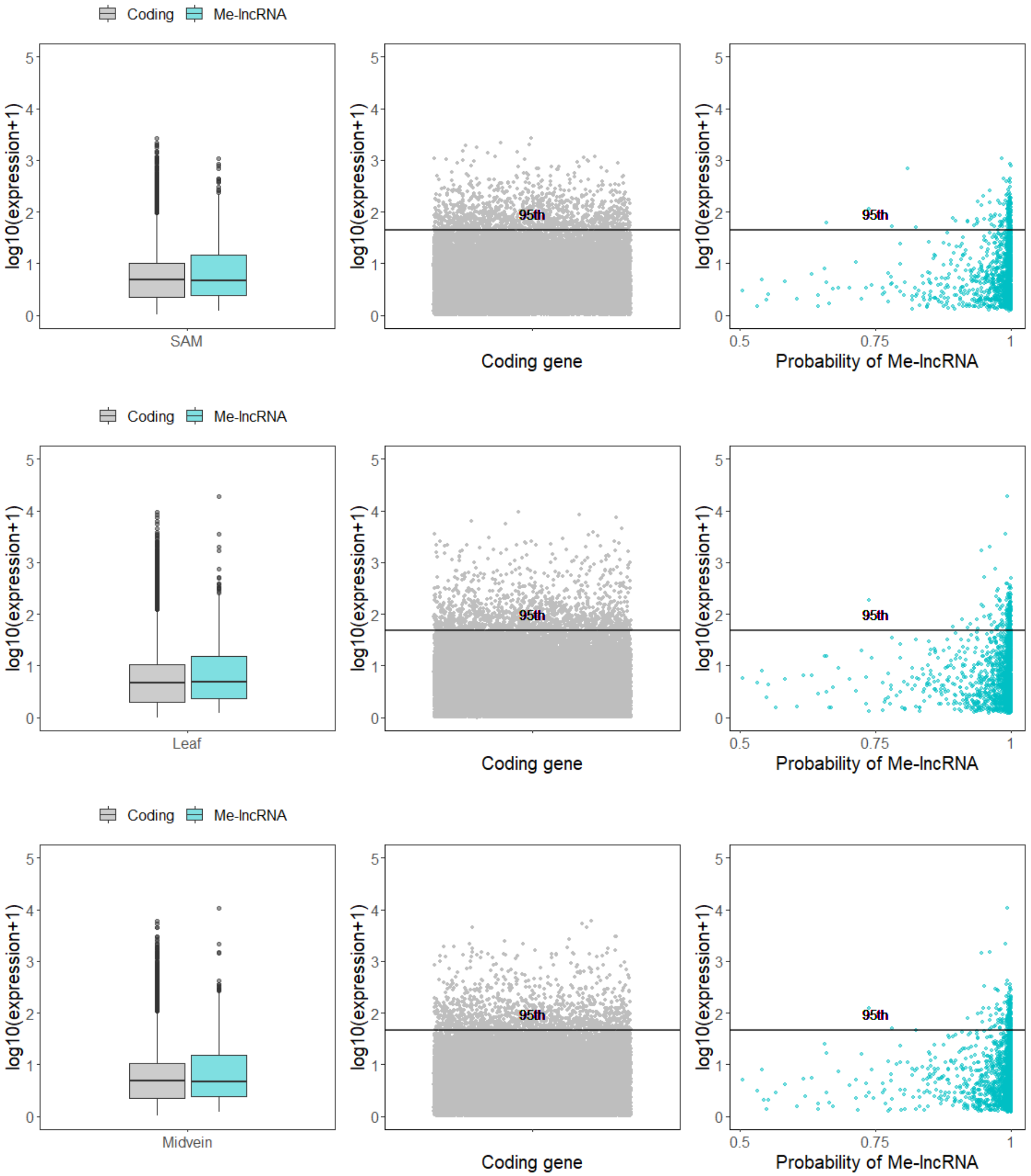

Figure S11: Comparison of expression level between Me-lncRNAs and protein coding genes in cassava RNA-seq data from Wilson [39]. Y-axis represents expression level with normalization by GeTMM. The left graph represents boxplot of expression level in coding genes and Me-lncRNAs. The middle graph represents scatterplot of expression distribution in coding genes. The right graph represents scatterplot of expression distribution in Me-lncRNAs. X-axis in the right graph determined the confidence (probability to be ncRNA) of Me-lncRNAs according to RNAz tool. Black line at y-intercept denotes 95th percentile rank of expression.

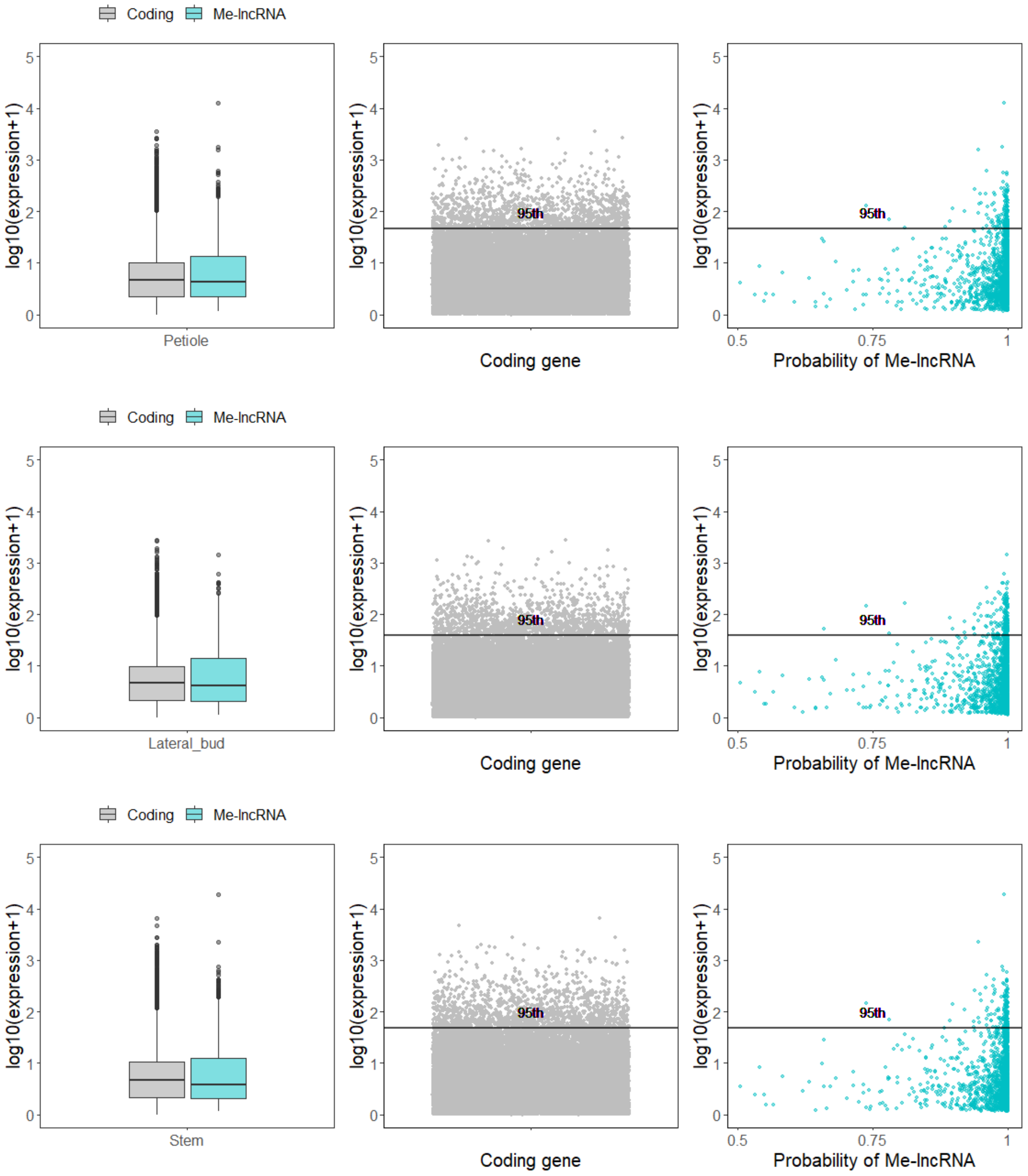

Figure S11: Comparison of expression level between Me-lncRNAs and protein coding genes in cassava RNA-seq data from Wilson [39]. Y-axis represents expression level with normalization by GeTMM. The left graph represents boxplot of expression level in coding genes and Me-lncRNAs. The middle graph represents scatterplot of expression distribution in coding genes. The right graph represents scatterplot of expression distribution in Me-lncRNAs. X-axis in the right graph determined the confidence (probability to be ncRNA) of Me-lncRNAs according to RNAz tool. Black line at y-intercept denotes 95th percentile rank of expression (continue).

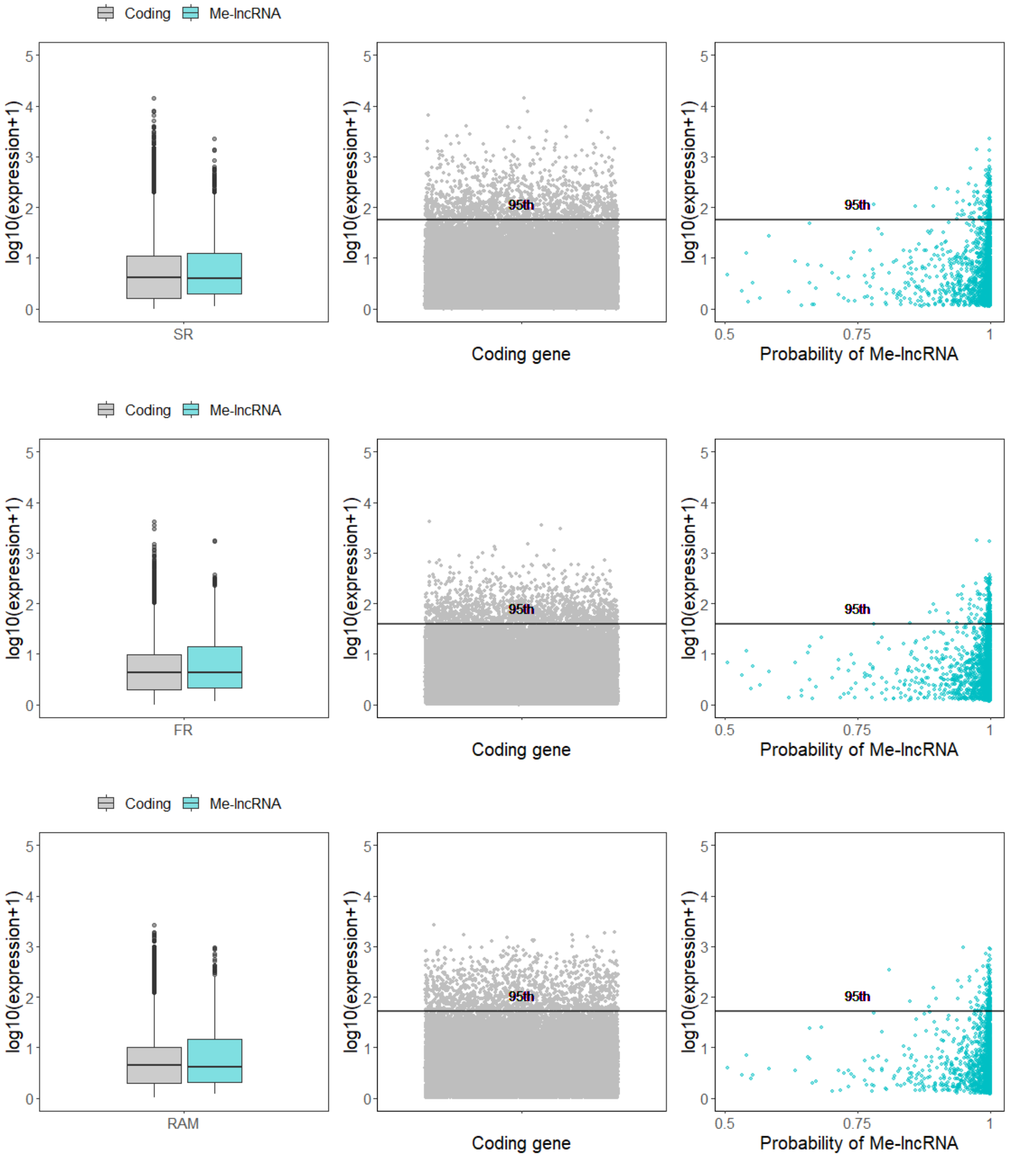

Figure S11: Comparison of expression level between Me-lncRNAs and protein coding genes in cassava RNA-seq data from Wilson [39]. Y-axis represents expression level with normalization by GeTMM. The left graph represents boxplot of expression level in coding genes and Me-lncRNAs. The middle graph represents scatterplot of expression distribution in coding genes. The right graph represents scatterplot of expression distribution in Me-lncRNAs. X-axis in the right graph determined the confidence (probability to be ncRNA) of Me-lncRNAs according to RNAz tool. Black line at y-intercept denotes 95th percentile rank of expression (continue).

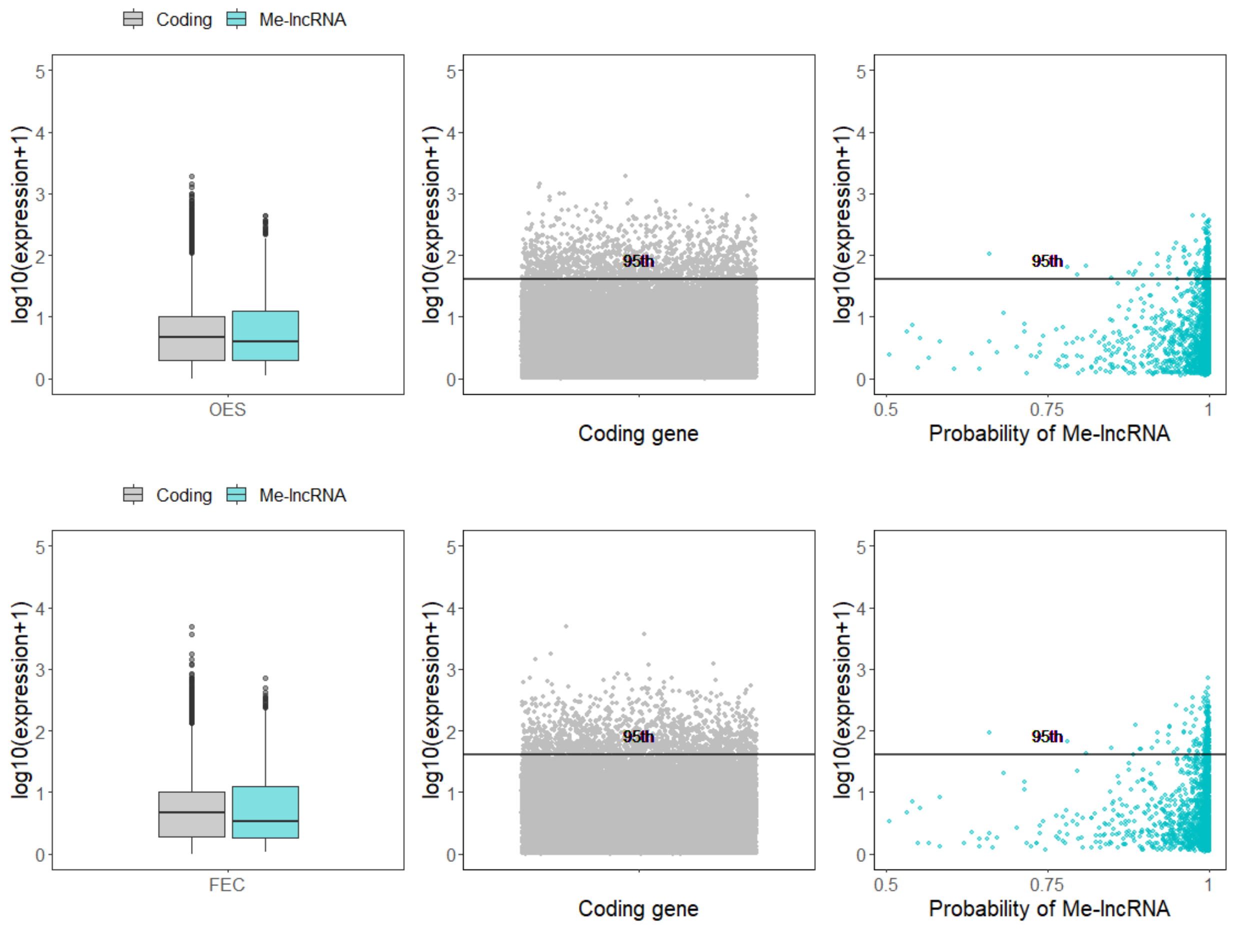

Figure S11: Comparison of expression level between Me-lncRNAs and protein coding genes in cassava RNA-seq data from Wilson [39]. Y-axis represents expression level with normalization by GeTMM. The left graph represents boxplot of expression level in coding genes and Me-lncRNAs. The middle graph represents scatterplot of expression distribution in coding genes. The right graph represents scatterplot of expression distribution in Me-lncRNAs. X-axis in the right graph determined the confidence (probability to be ncRNA) of Me-lncRNAs according to RNAz tool. Black line at y-intercept denotes 95th percentile rank of expression (continue).

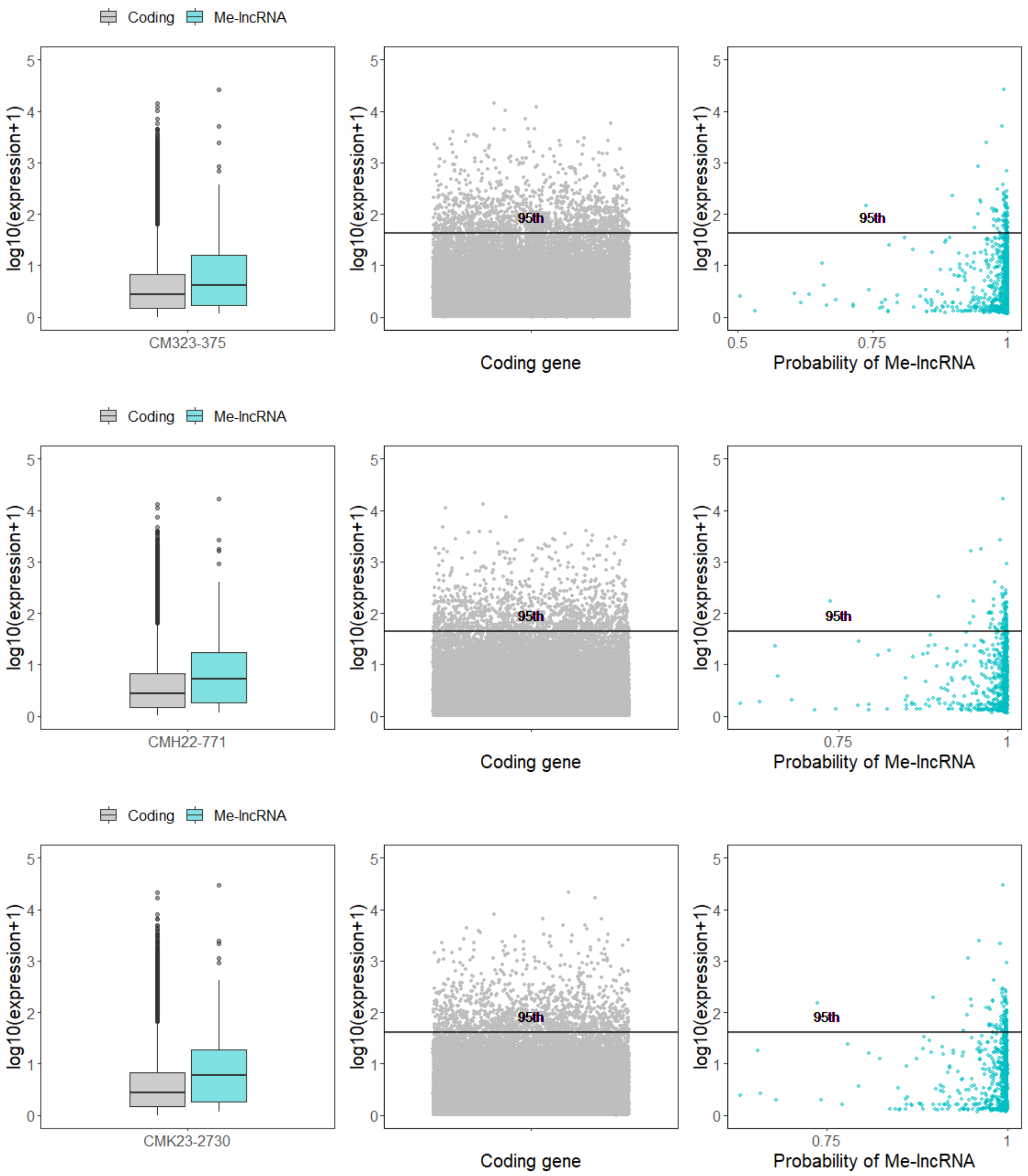

Figure S12: Comparison of expression level between Me-lncRNAs and protein coding genes in cassava RNA-seq data from Pootakham [41]. Y-axis represents expression level with normalization by GeTMM. The left graph represents boxplot of expression level in coding genes and Me-lncRNAs. The middle graph represents scatterplot of expression distribution in coding genes. The right graph represents scatterplot of expression distribution in Me-lncRNAs. X-axis in the right graph determined the confidence (probability to be ncRNA) of Me-lncRNAs according to RNAz tool. Black line at y-intercept denotes 95th percentile rank of expression.

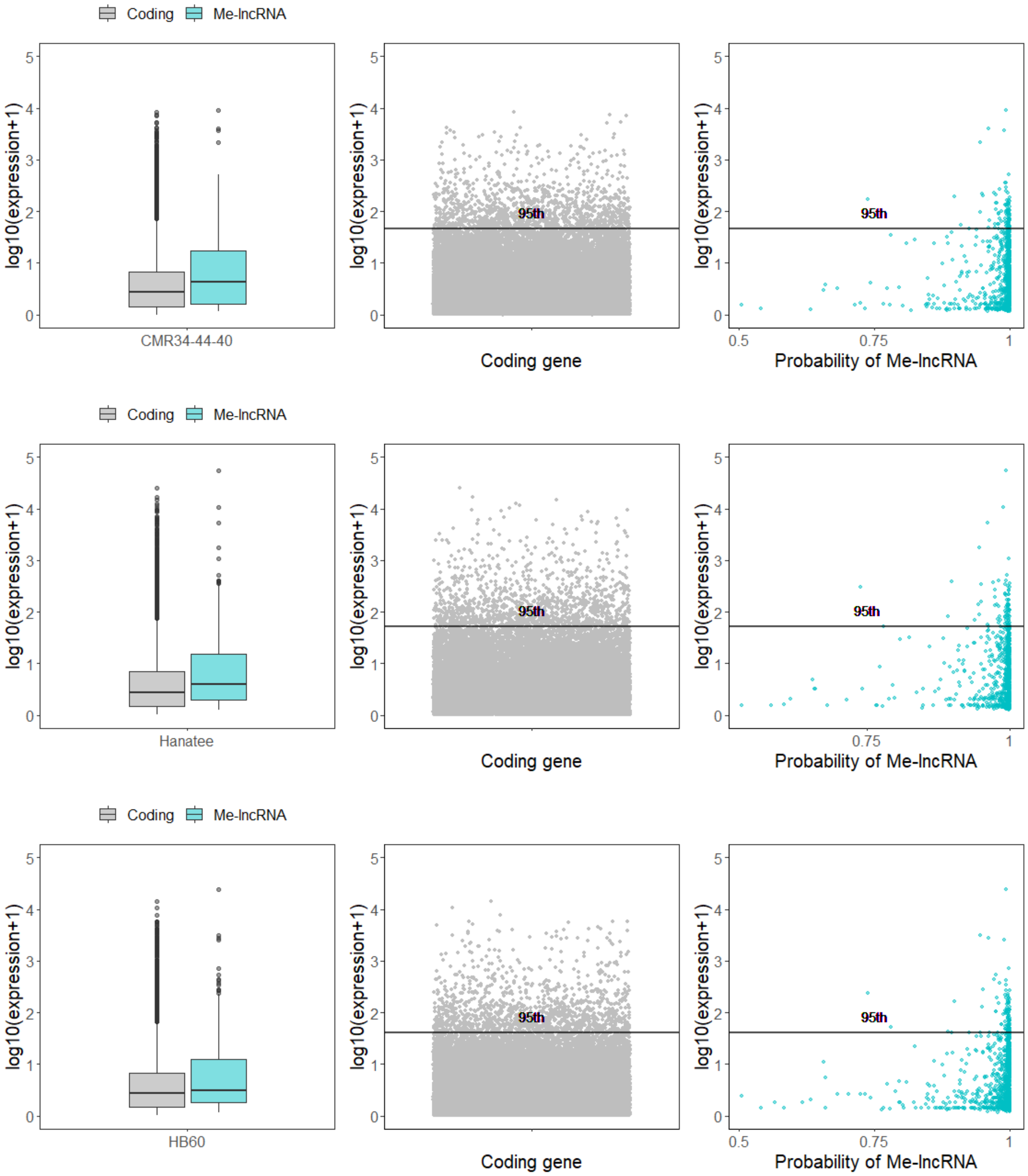

Figure S12: Comparison of expression level between Me-lncRNAs and protein coding genes in cassava RNA-seq data from Pootakham [41]. Y-axis represents expression level with normalization by GeTMM. The left graph represents boxplot of expression level in coding genes and Me-lncRNAs. The middle graph represents scatterplot of expression distribution in coding genes. The right graph represents scatterplot of expression distribution in Me-lncRNAs. X-axis in the right graph determined the confidence (probability to be ncRNA) of Me-lncRNAs according to RNAz tool. Black line at y-intercept denotes 95th percentile rank of expression (continue).

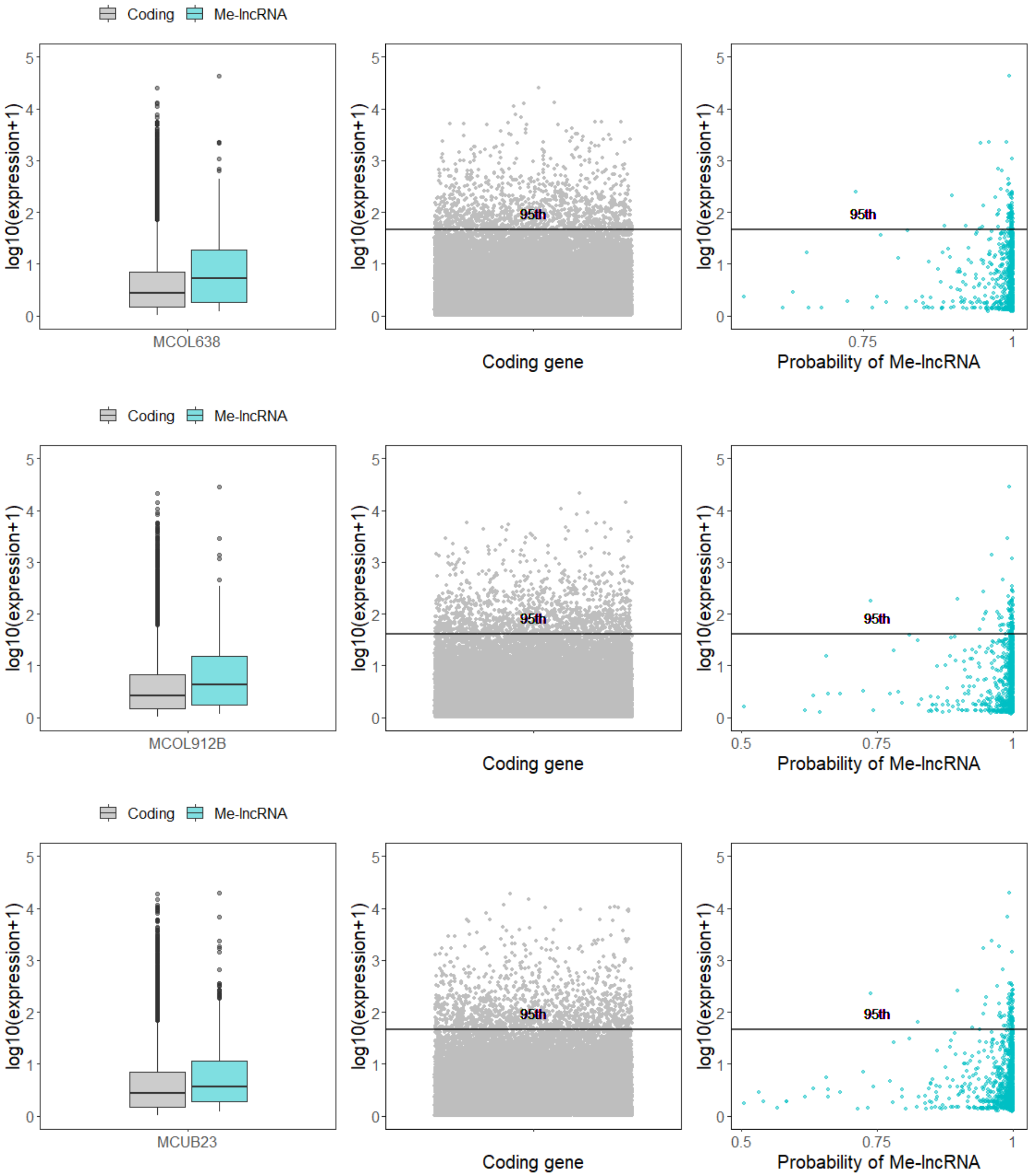

Figure S12: Comparison of expression level between Me-lncRNAs and protein coding genes in cassava RNA-seq data from Pootakham [41]. Y-axis represents expression level with normalization by GeTMM. The left graph represents boxplot of expression level in coding genes and Me-lncRNAs. The middle graph represents scatterplot of expression distribution in coding genes. The right graph represents scatterplot of expression distribution in Me-lncRNAs. X-axis in the right graph determined the confidence (probability to be ncRNA) of Me-lncRNAs according to RNAz tool. Black line at y-intercept denotes 95th percentile rank of expression (continue).

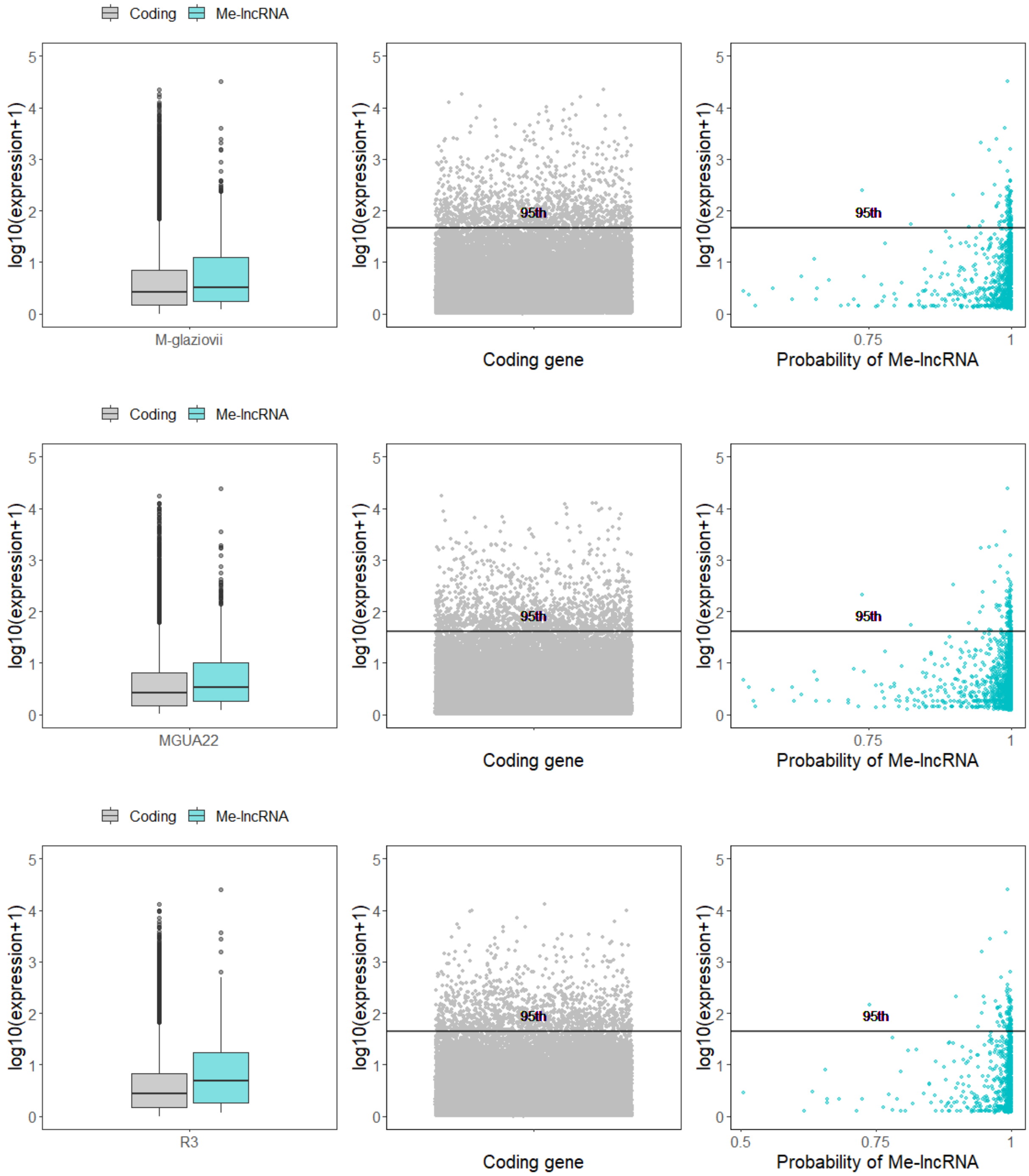

Figure S12: Comparison of expression level between Me-lncRNAs and protein coding genes in cassava RNA-seq data from Pootakham [41]. Y-axis represents expression level with normalization by GeTMM. The left graph represents boxplot of expression level in coding genes and Me-lncRNAs. The middle graph represents scatterplot of expression distribution in coding genes. The right graph represents scatterplot of expression distribution in Me-lncRNAs. X-axis in the right graph determined the confidence (probability to be ncRNA) of Me-lncRNAs according to RNAz tool. Black line at y-intercept denotes 95th percentile rank of expression (continue).

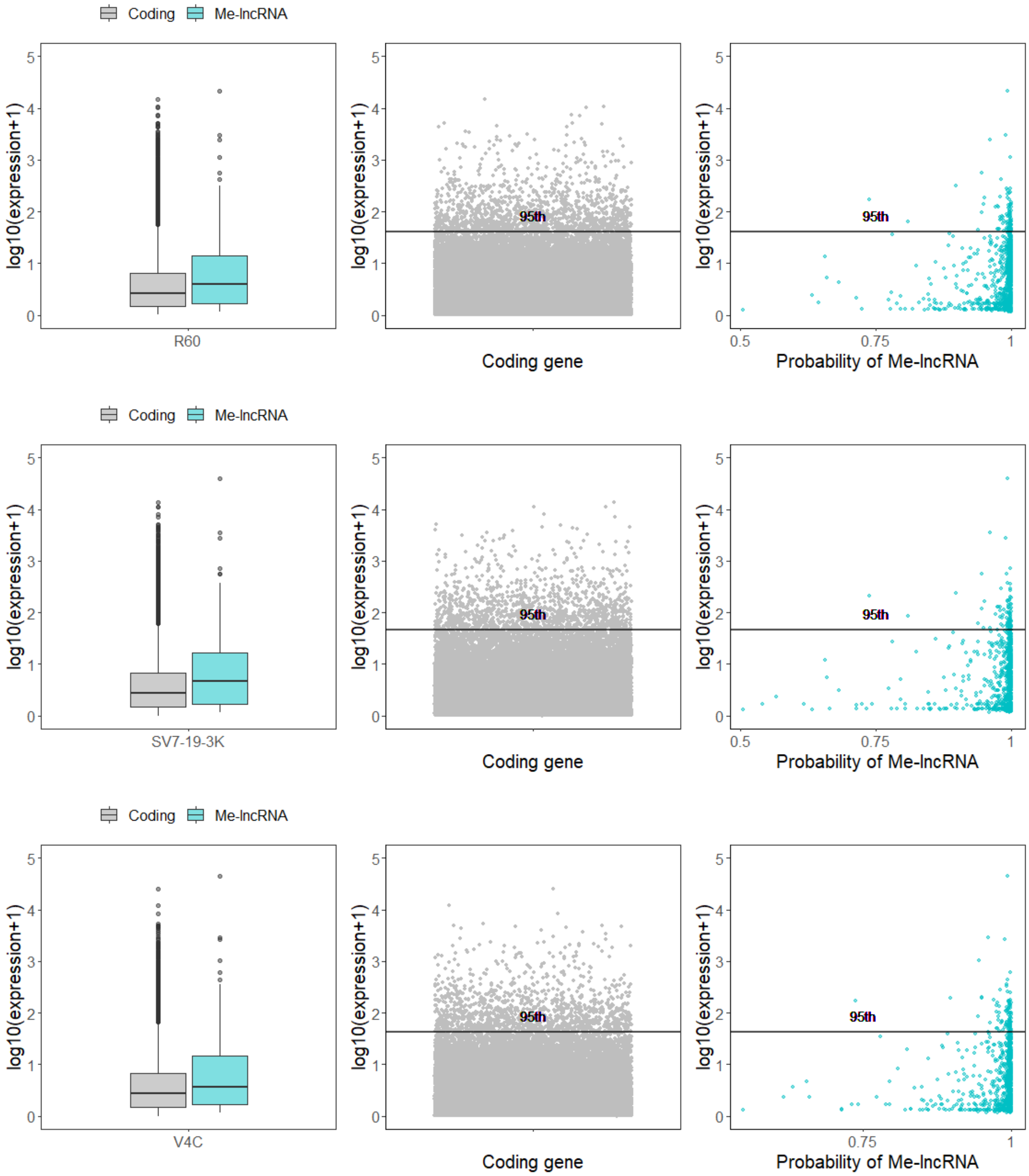

Figure S12: Comparison of expression level between Me-lncRNAs and protein coding genes in cassava RNA-seq data from Pootakham [41]. Y-axis represents expression level with normalization by GeTMM. The left graph represents boxplot of expression level in coding genes and Me-lncRNAs. The middle graph represents scatterplot of expression distribution in coding genes. The right graph represents scatterplot of expression distribution in Me-lncRNAs. X-axis in the right graph determined the confidence (probability to be ncRNA) of Me-lncRNAs according to RNAz tool. Black line at y-intercept denotes 95th percentile rank of expression (continue).

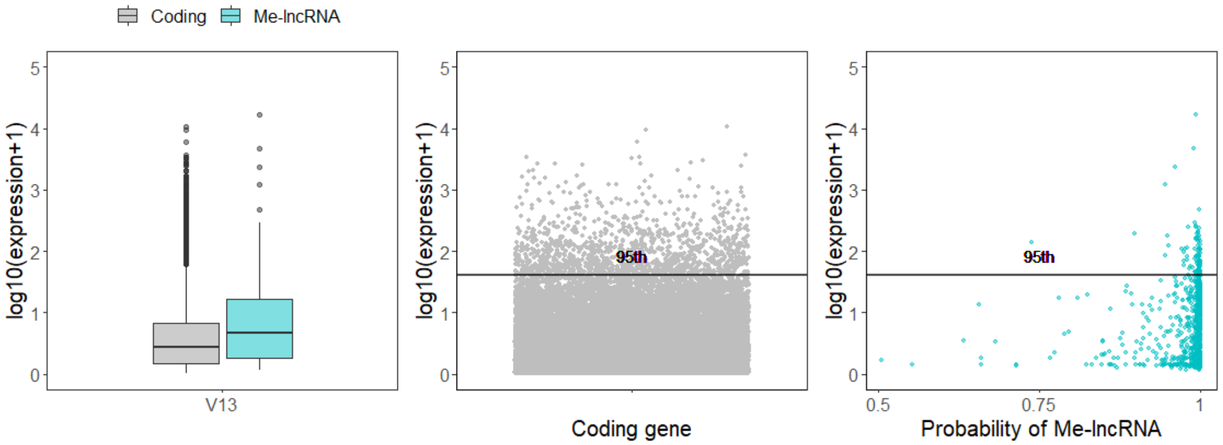

Figure S12: Comparison of expression level between Me-lncRNAs and protein coding genes in cassava RNA-seq data from Pootakham [41]. Y-axis represents expression level with normalization by GeTMM. The left graph represents boxplot of expression level in coding genes and Me-lncRNAs. The middle graph represents scatterplot of expression distribution in coding genes. The right graph represents scatterplot of expression distribution in Me-lncRNAs. X-axis in the right graph determined the confidence (probability to be ncRNA) of Me-lncRNAs according to RNAz tool. Black line at y-intercept denotes 95th percentile rank of expression (continue).

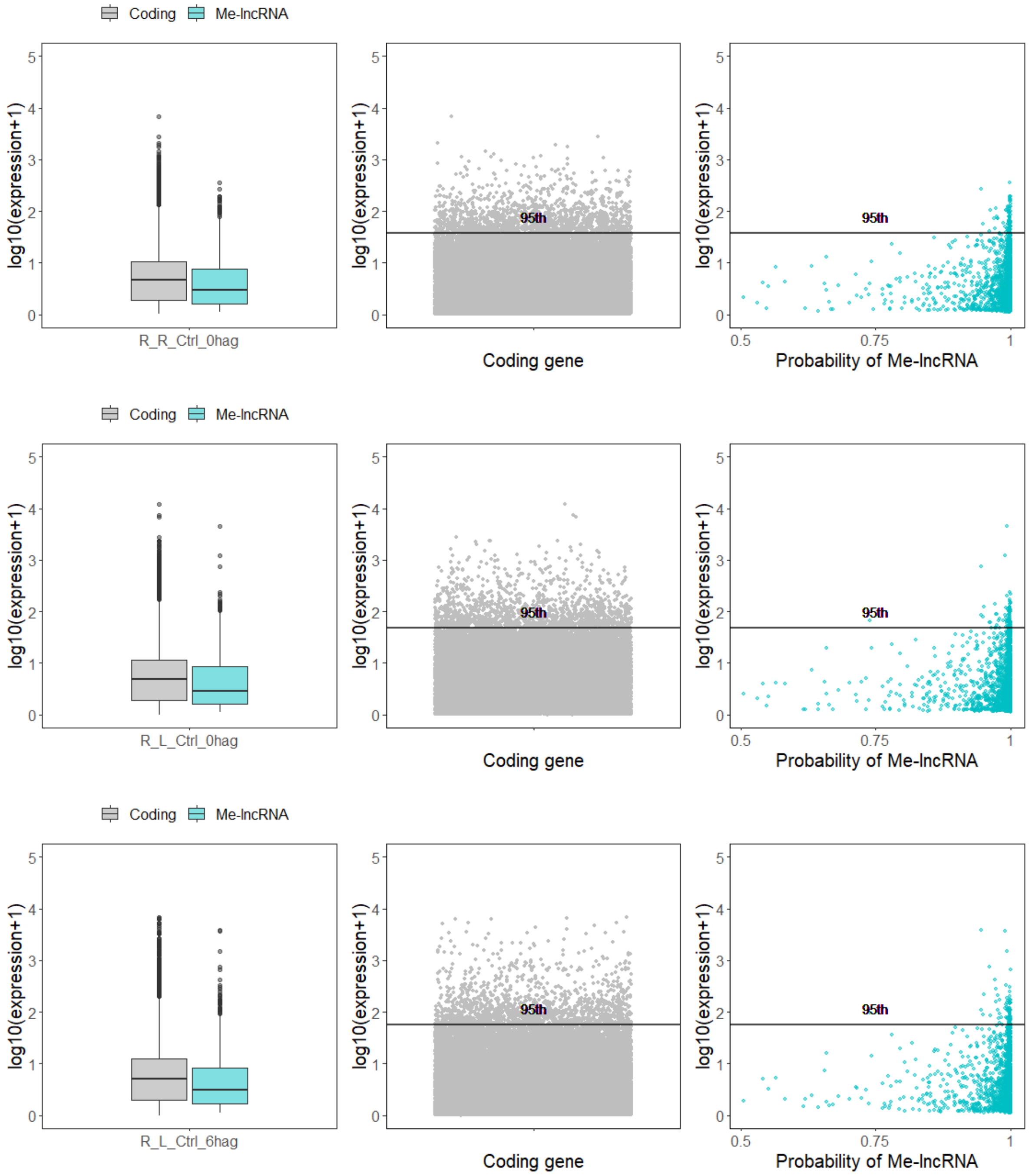

Figure S13: Comparison of expression level between Me-lncRNAs and protein coding genes in CBSV-resistant cassava RNA-seq data from Amuge [40]. Y-axis represents expression level with normalization by GeTMM. The left graph represents boxplot of expression level in coding genes and Me-lncRNAs. The middle graph represents scatterplot of expression distribution in coding genes. The right graph represents scatterplot of expression distribution in Me-lncRNAs. X-axis in the right graph determined the confidence (probability to be ncRNA) of Me-lncRNAs according to RNAz tool. Black line at y-intercept denotes 95th percentile rank of expression.

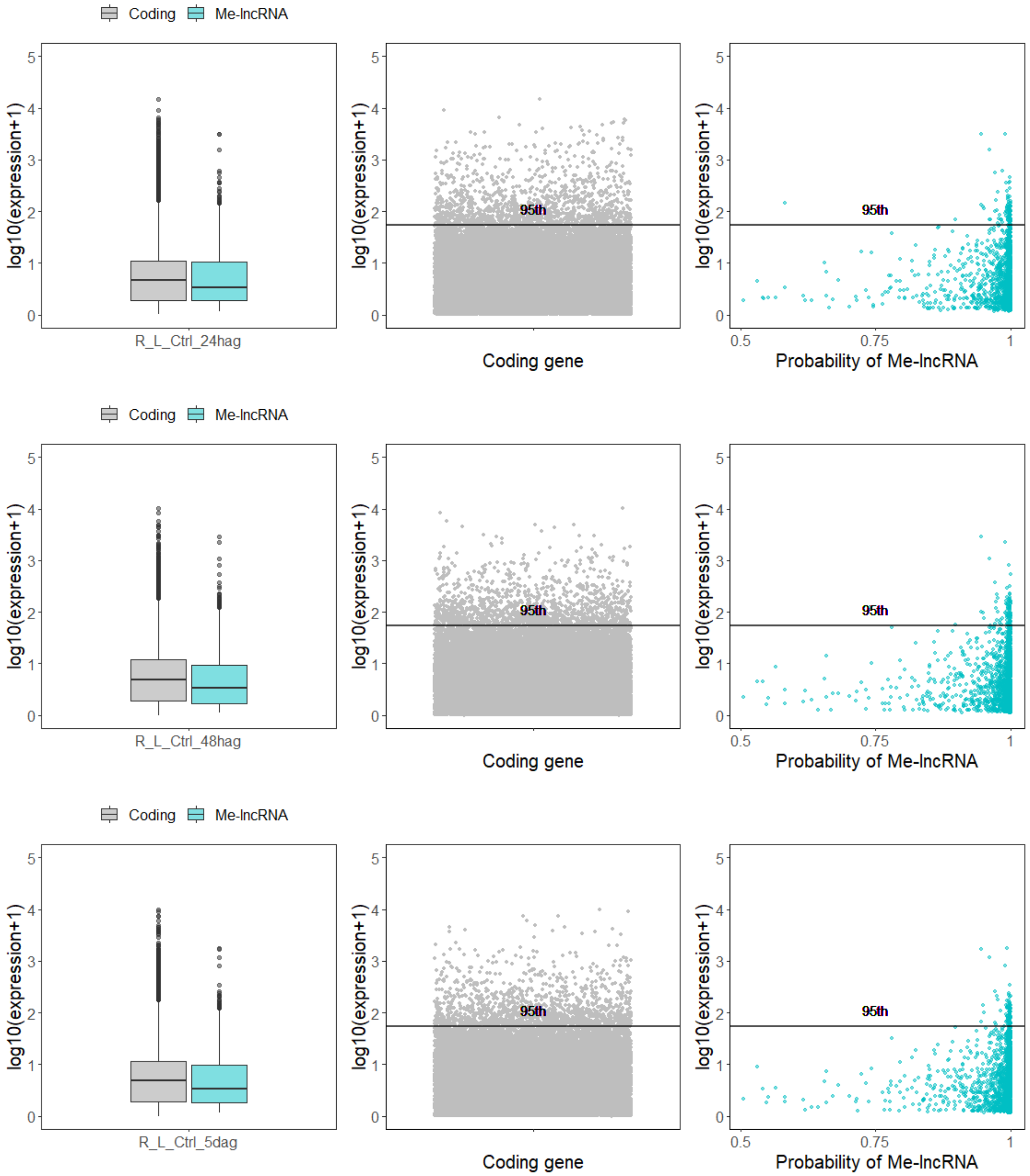

Figure S13: Comparison of expression level between Me-lncRNAs and protein coding genes in CBSV-resistant cassava RNA-seq data from Amuge [40]. Y-axis represents expression level with normalization by GeTMM. The left graph represents boxplot of expression level in coding genes and Me-lncRNAs. The middle graph represents scatterplot of expression distribution in coding genes. The right graph represents scatterplot of expression distribution in Me-lncRNAs. X-axis in the right graph determined the confidence (probability to be ncRNA) of Me-lncRNAs according to RNAz tool. Black line at y-intercept denotes 95th percentile rank of expression (continue).

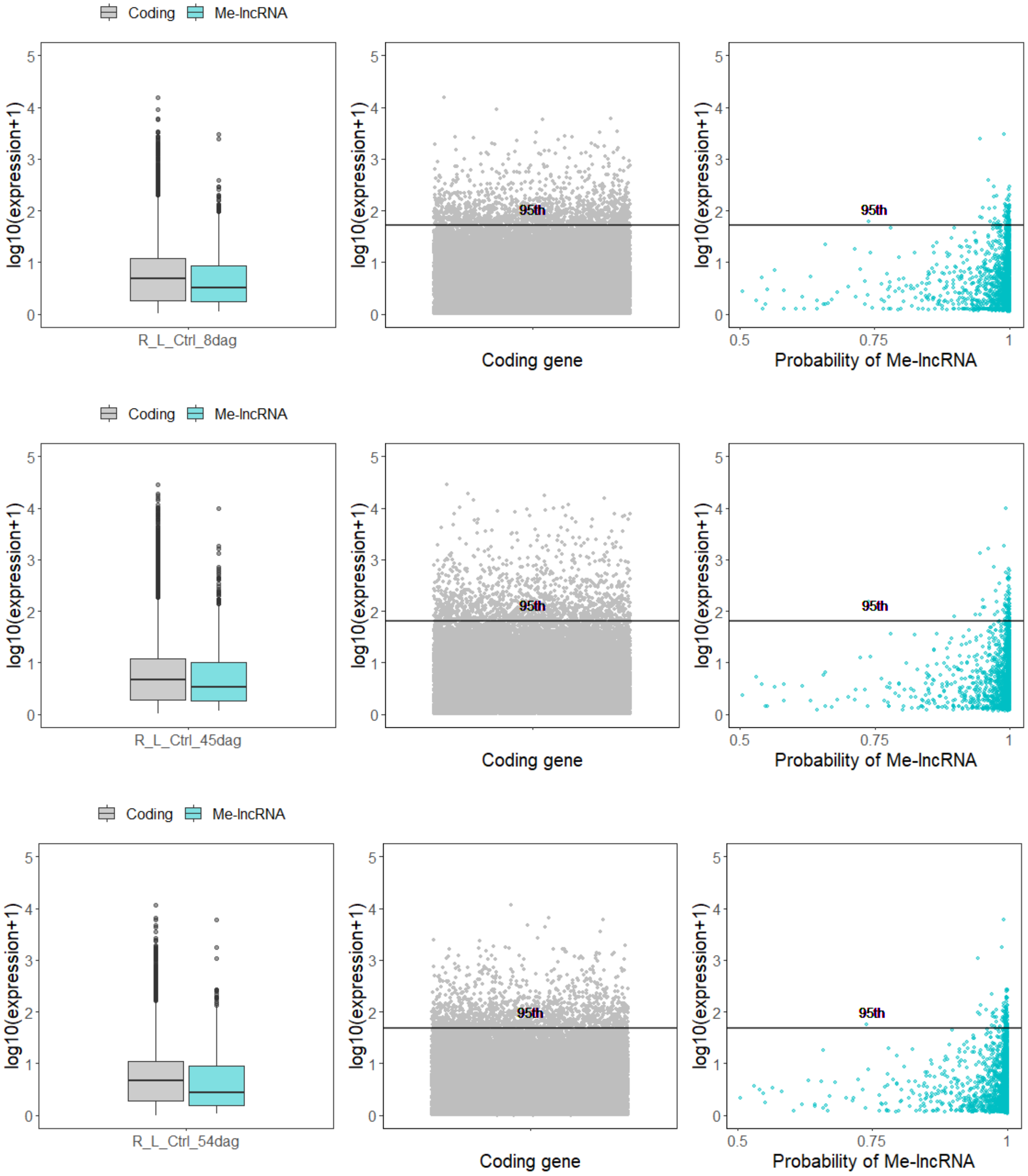

Figure S13: Comparison of expression level between Me-lncRNAs and protein coding genes in CBSV-resistant cassava RNA-seq data from Amuge [40]. Y-axis represents expression level with normalization by GeTMM. The left graph represents boxplot of expression level in coding genes and Me-lncRNAs. The middle graph represents scatterplot of expression distribution in coding genes. The right graph represents scatterplot of expression distribution in Me-lncRNAs. X-axis in the right graph determined the confidence (probability to be ncRNA) of Me-lncRNAs according to RNAz tool. Black line at y-intercept denotes 95th percentile rank of expression (continue).

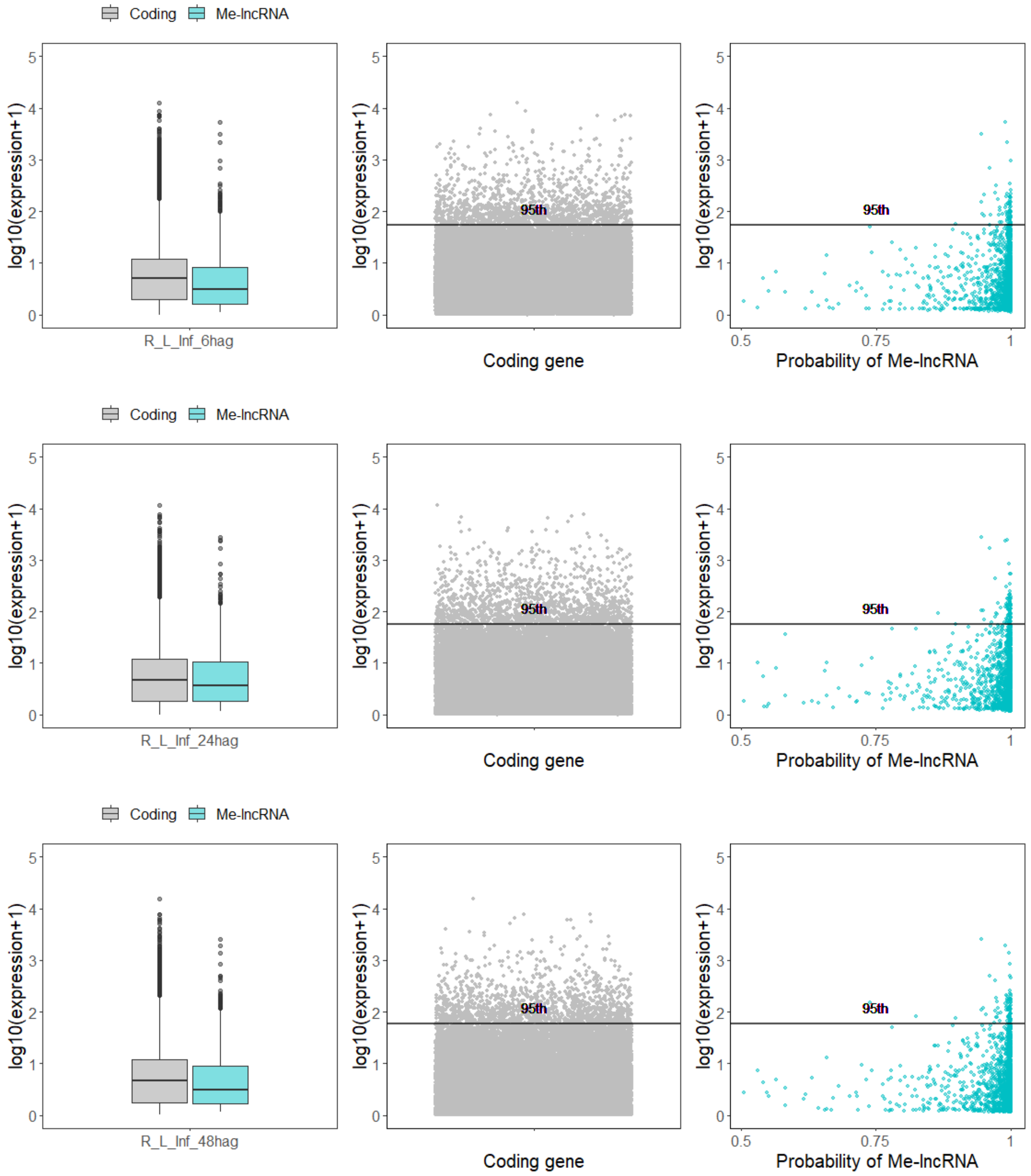

Figure S13: Comparison of expression level between Me-lncRNAs and protein coding genes in CBSV-resistant cassava RNA-seq data from Amuge [40]. Y-axis represents expression level with normalization by GeTMM. The left graph represents boxplot of expression level in coding genes and Me-lncRNAs. The middle graph represents scatterplot of expression distribution in coding genes. The right graph represents scatterplot of expression distribution in Me-lncRNAs. X-axis in the right graph determined the confidence (probability to be ncRNA) of Me-lncRNAs according to RNAz tool. Black line at y-intercept denotes 95th percentile rank of expression (continue).

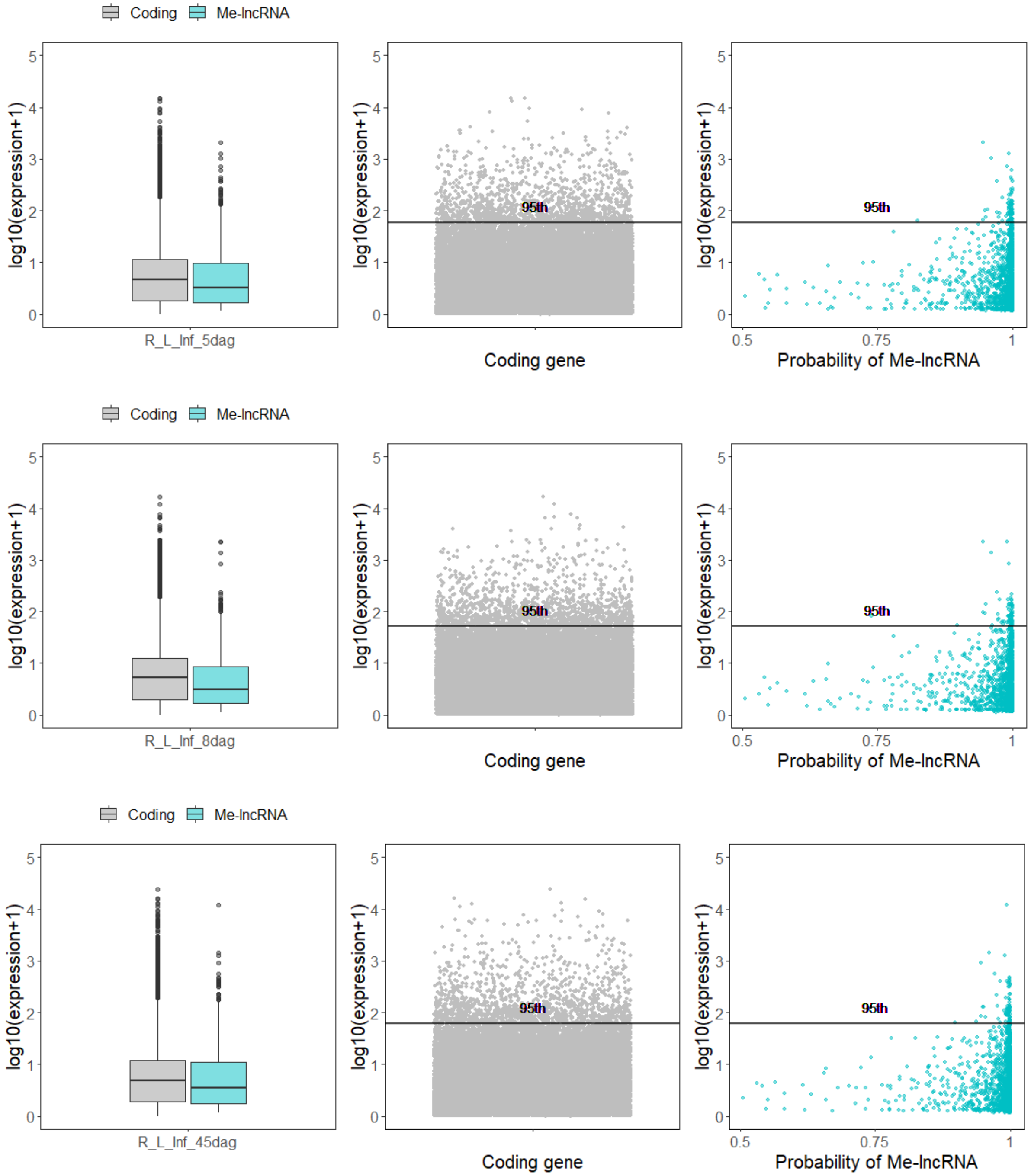

Figure S13: Comparison of expression level between Me-lncRNAs and protein coding genes in CBSV-resistant cassava RNA-seq data from Amuge [40]. Y-axis represents expression level with normalization by GeTMM. The left graph represents boxplot of expression level in coding genes and Me-lncRNAs. The middle graph represents scatterplot of expression distribution in coding genes. The right graph represents scatterplot of expression distribution in Me-lncRNAs. X-axis in the right graph determined the confidence (probability to be ncRNA) of Me-lncRNAs according to RNAz tool. Black line at y-intercept denotes 95th percentile rank of expression (continue).

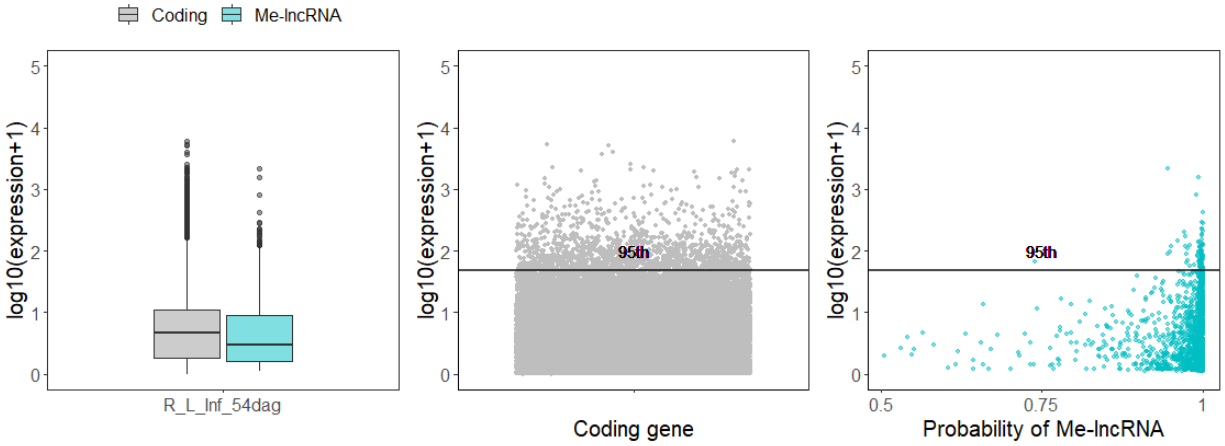

Figure S13: Comparison of expression level between Me-lncRNAs and protein coding genes in CBSV-resistant cassava RNA-seq data from Amuge [40]. Y-axis represents expression level with normalization by GeTMM. The left graph represents boxplot of expression level in coding genes and Me-lncRNAs. The middle graph represents scatterplot of expression distribution in coding genes. The right graph represents scatterplot of expression distribution in Me-lncRNAs. X-axis in the right graph determined the confidence (probability to be ncRNA) of Me-lncRNAs according to RNAz tool. Black line at y-intercept denotes 95th percentile rank of expression (continue).

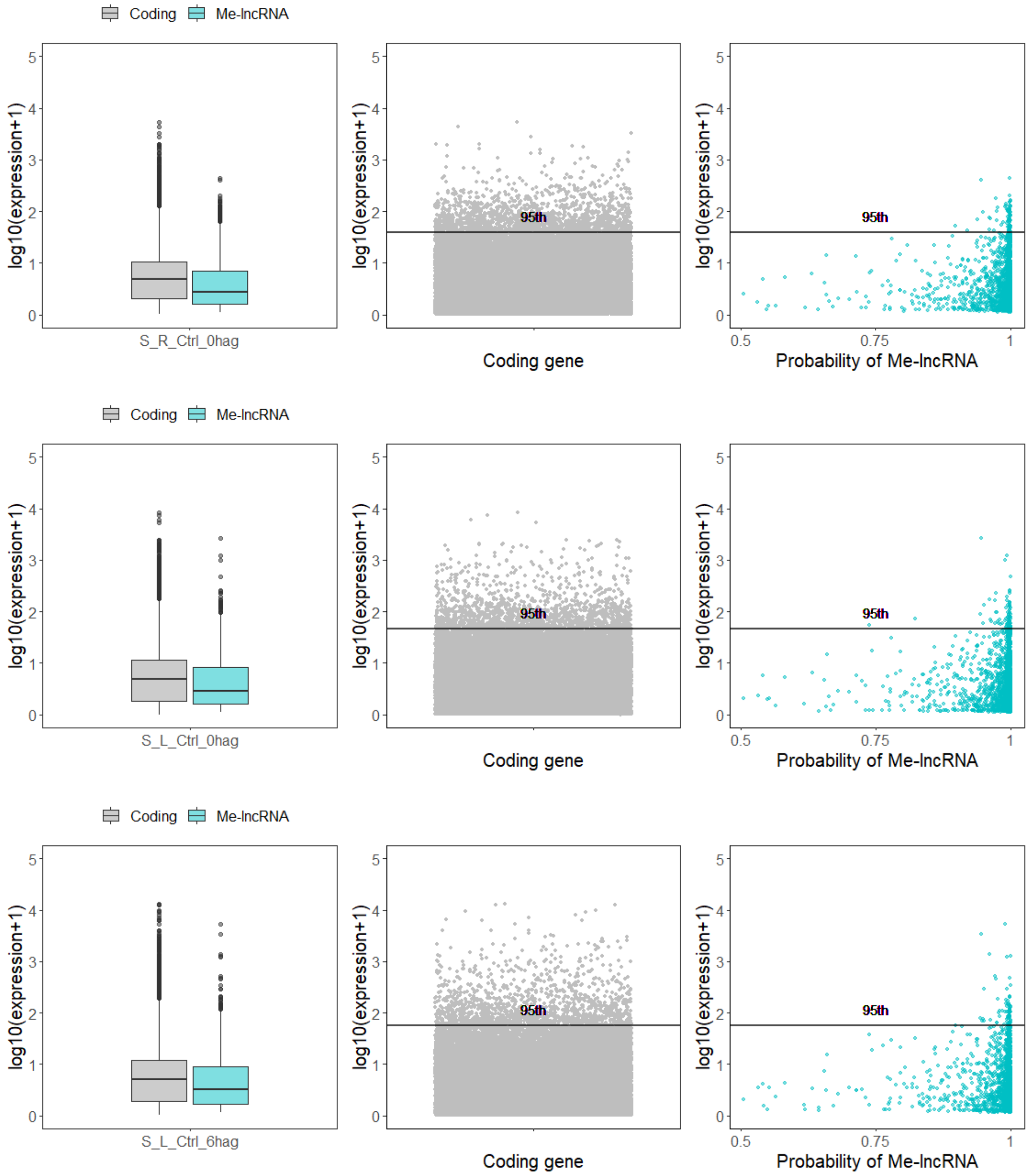

Figure S14: Comparison of expression level between Me-lncRNAs and protein coding genes in CBSV-susceptible cassava RNA-seq data from Amuge [40]. Y-axis represents expression level with normalization by GeTMM. The left graph represents boxplot of expression level in coding genes and Me-lncRNAs. The middle graph represents scatterplot of expression distribution in coding genes. The right graph represents scatterplot of expression distribution in Me-lncRNAs. X-axis in the right graph determined the confidence (probability to be ncRNA) of Me-lncRNAs according to RNAz tool. Black line at y-intercept denotes 95th percentile rank of expression.

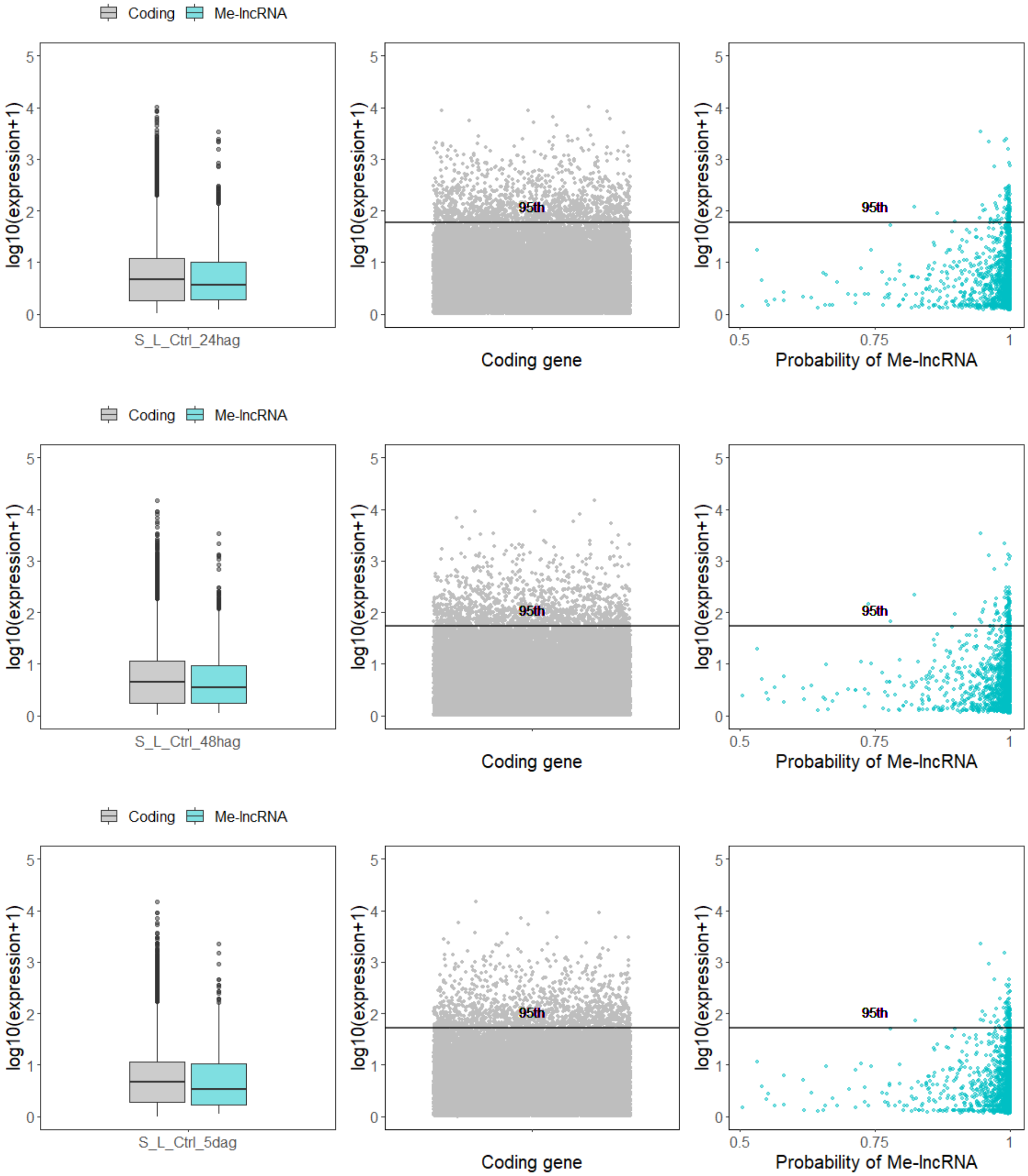

Figure S14: Comparison of expression level between Me-lncRNAs and protein coding genes in CBSV-susceptible cassava RNA-seq data from Amuge [40]. Y-axis represents expression level with normalization by GeTMM. The left graph represents boxplot of expression level in coding genes and Me-lncRNAs. The middle graph represents scatterplot of expression distribution in coding genes. The right graph represents scatterplot of expression distribution in Me-lncRNAs. X-axis in the right graph determined the confidence (probability to be ncRNA) of Me-lncRNAs according to RNAz tool. Black line at y-intercept denotes 95th percentile rank of expression (continue).

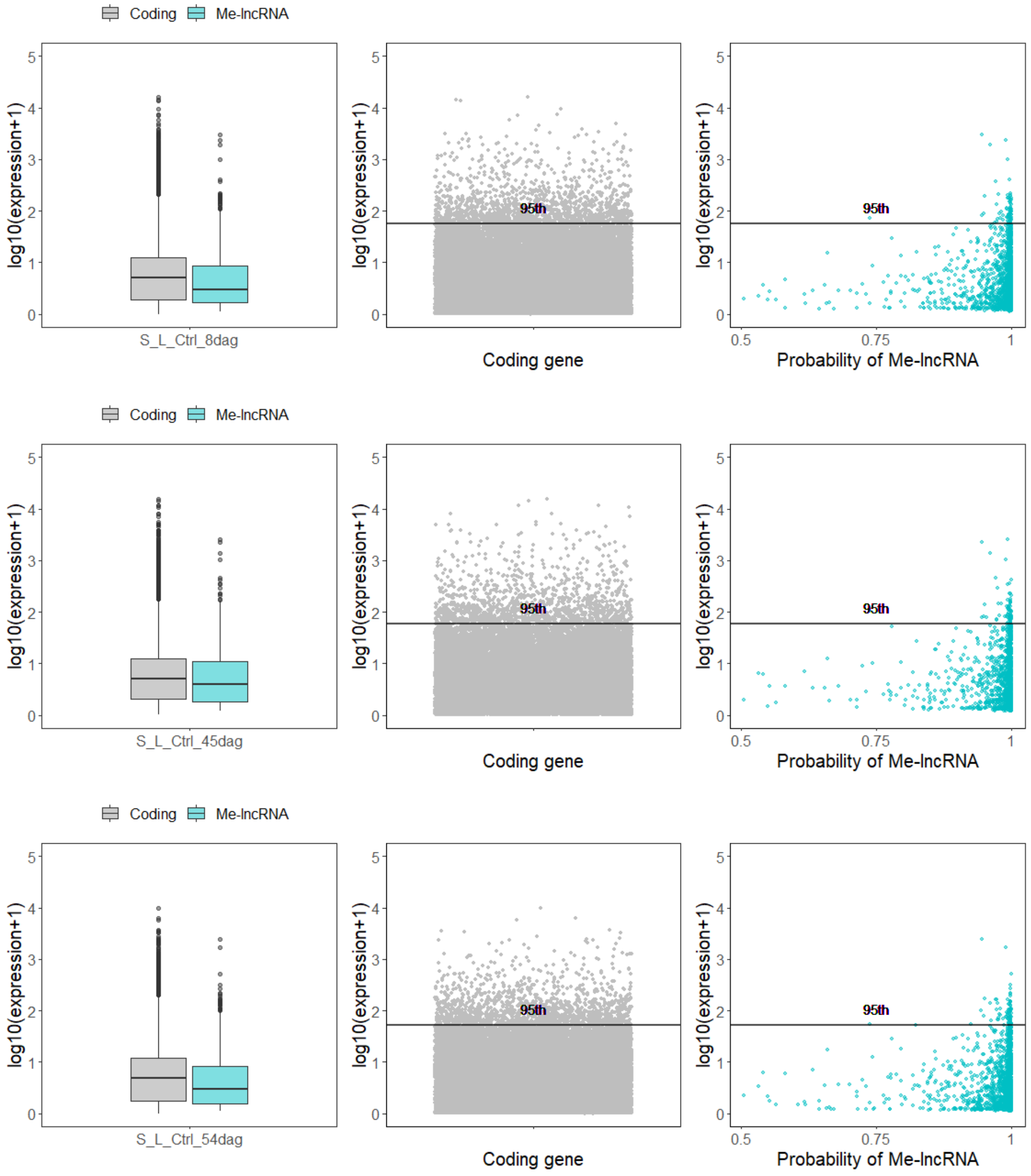

Figure S14: Comparison of expression level between Me-lncRNAs and protein coding genes in CBSV-susceptible cassava RNA-seq data from Amuge [40]. Y-axis represents expression level with normalization by GeTMM. The left graph represents boxplot of expression level in coding genes and Me-lncRNAs. The middle graph represents scatterplot of expression distribution in coding genes. The right graph represents scatterplot of expression distribution in Me-lncRNAs. X-axis in the right graph determined the confidence (probability to be ncRNA) of Me-lncRNAs according to RNAz tool. Black line at y-intercept denotes 95th percentile rank of expression (continue).

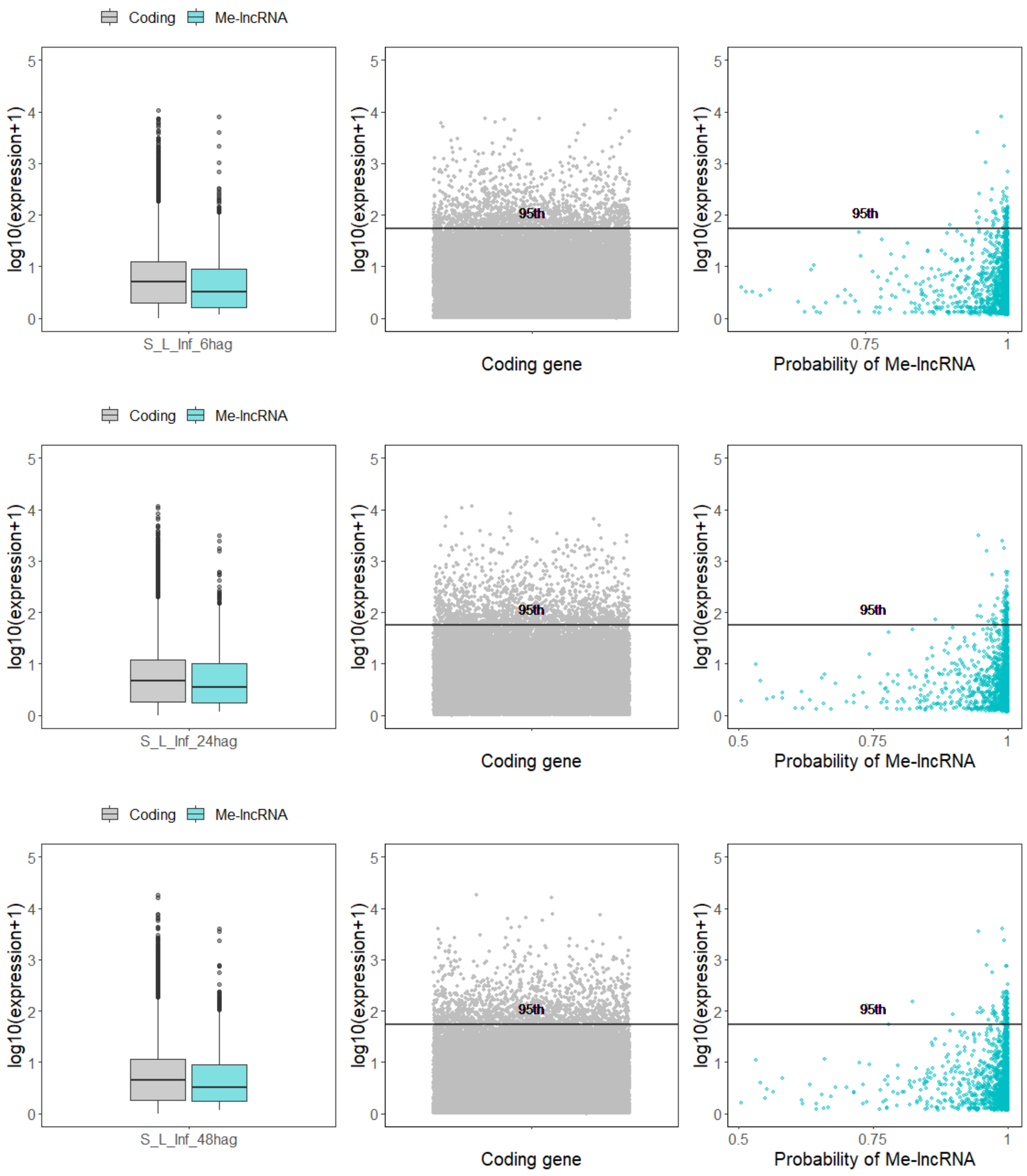

Figure S14: Comparison of expression level between Me-lncRNAs and protein coding genes in CBSV-susceptible cassava RNA-seq data from Amuge [40]. Y-axis represents expression level with normalization by GeTMM. The left graph represents boxplot of expression level in coding genes and Me-lncRNAs. The middle graph represents scatterplot of expression distribution in coding genes. The right graph represents scatterplot of expression distribution in Me-lncRNAs. X-axis in the right graph determined the confidence (probability to be ncRNA) of Me-lncRNAs according to RNAz tool. Black line at y-intercept denotes 95th percentile rank of expression (continue).

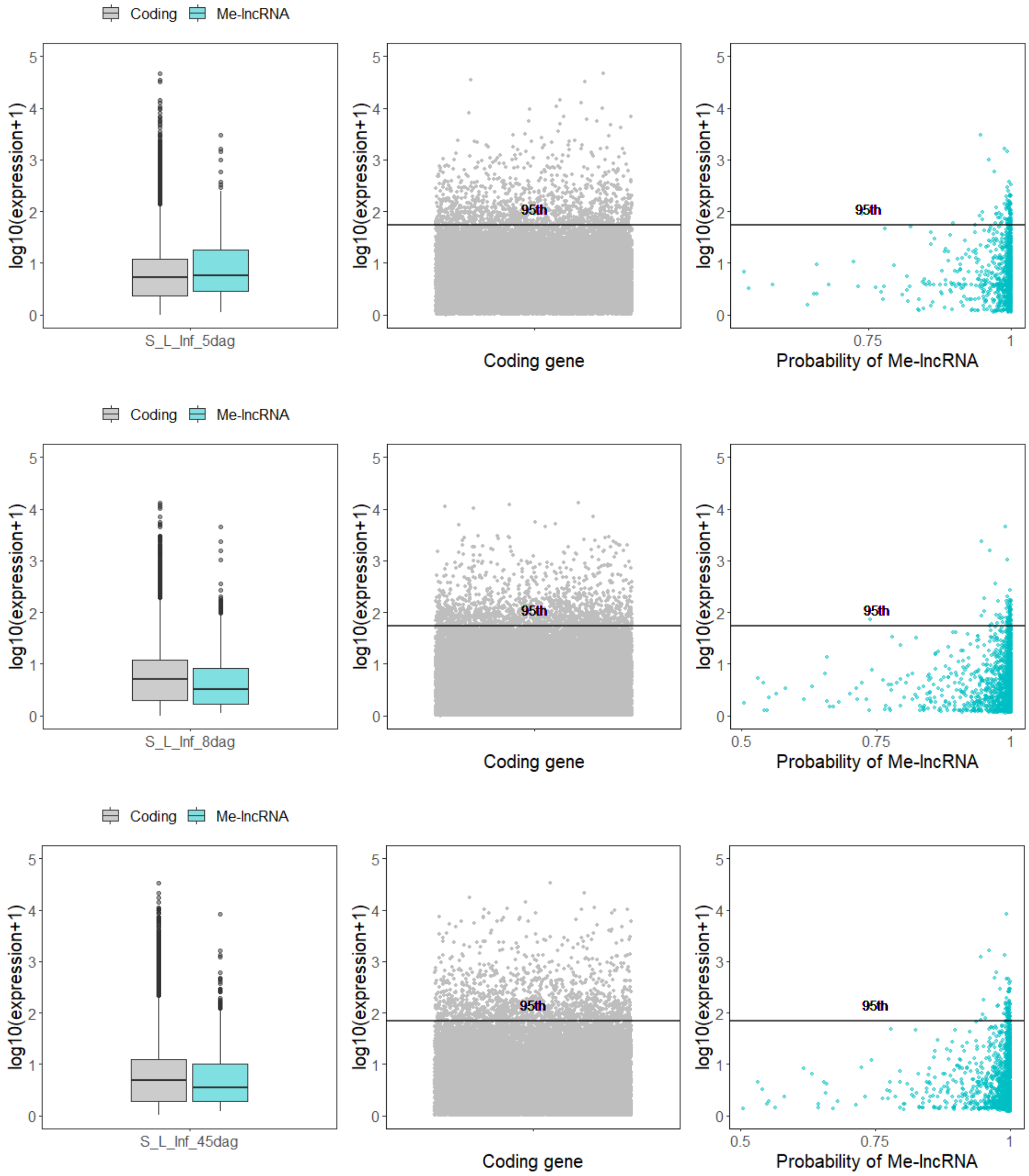

Figure S14: Comparison of expression level between Me-lncRNAs and protein coding genes in CBSV-susceptible cassava RNA-seq data from Amuge [40]. Y-axis represents expression level with normalization by GeTMM. The left graph represents boxplot of expression level in coding genes and Me-lncRNAs. The middle graph represents scatterplot of expression distribution in coding genes. The right graph represents scatterplot of expression distribution in Me-lncRNAs. X-axis in the right graph determined the confidence (probability to be ncRNA) of Me-lncRNAs according to RNAz tool. Black line at y-intercept denotes 95th percentile rank of expression (continue).

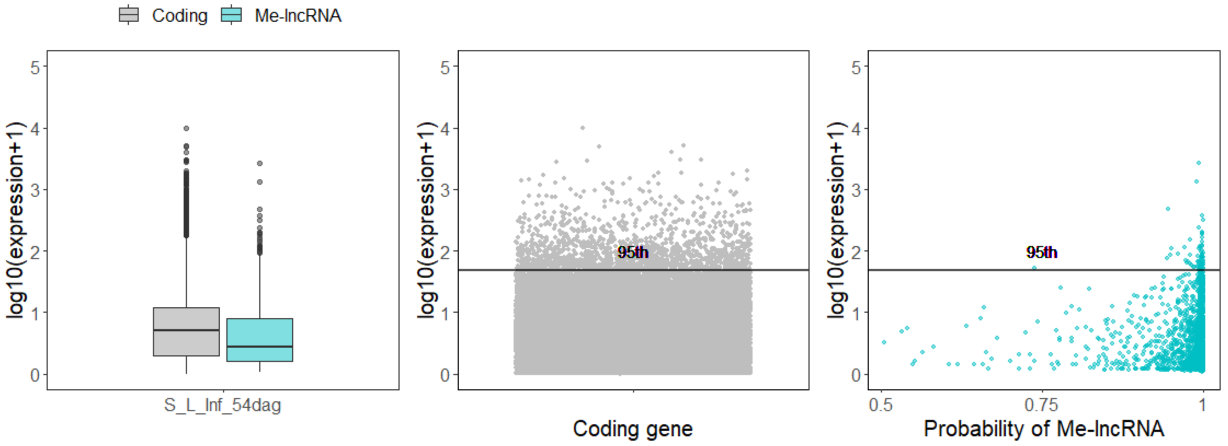

Figure S14: Comparison of expression level between Me-lncRNAs and protein coding genes in CBSV-susceptible cassava RNA-seq data from Amuge [40]. Y-axis represents expression level with normalization by GeTMM. The left graph represents boxplot of expression level in coding genes and Me-lncRNAs. The middle graph represents scatterplot of expression distribution in coding genes. The right graph represents scatterplot of expression distribution in Me-lncRNAs. X-axis in the right graph determined the confidence (probability to be ncRNA) of Me-lncRNAs according to RNAz tool. Black line at y-intercept denotes 95th percentile rank of expression (continue).

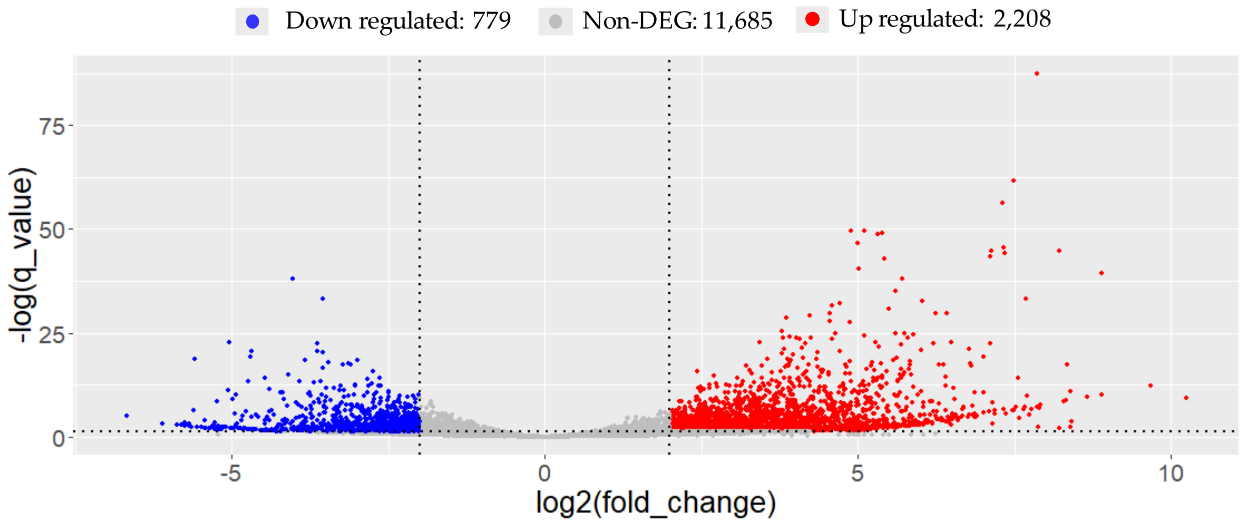

Figure S15: Volcano plot represents differentially expressed coding genes under cold stress of cassava.

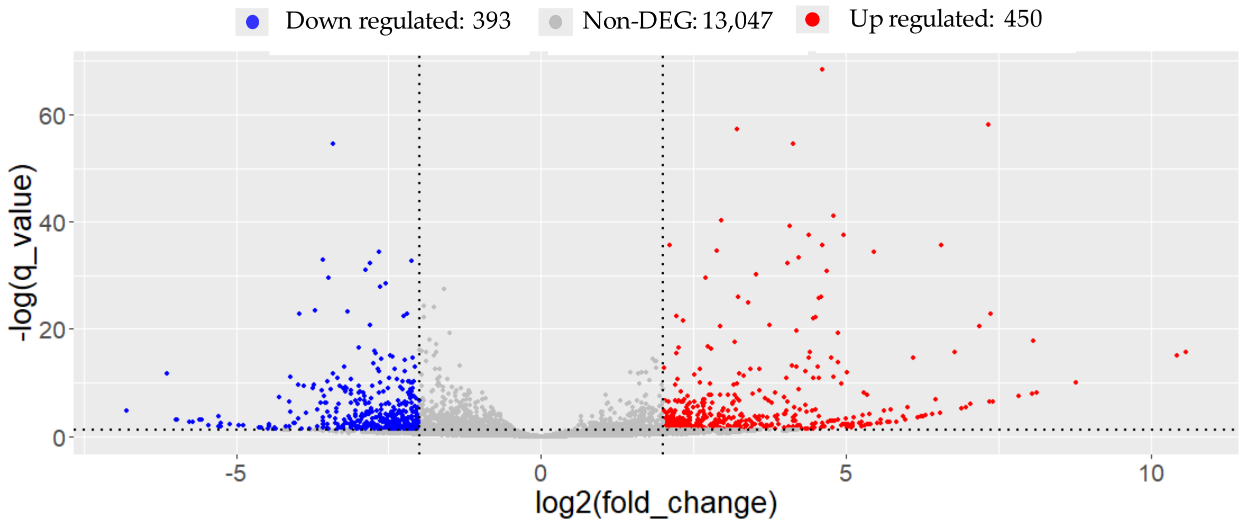

Figure S16: Volcano plot represents differentially expressed coding genes under drought stress of cassava.

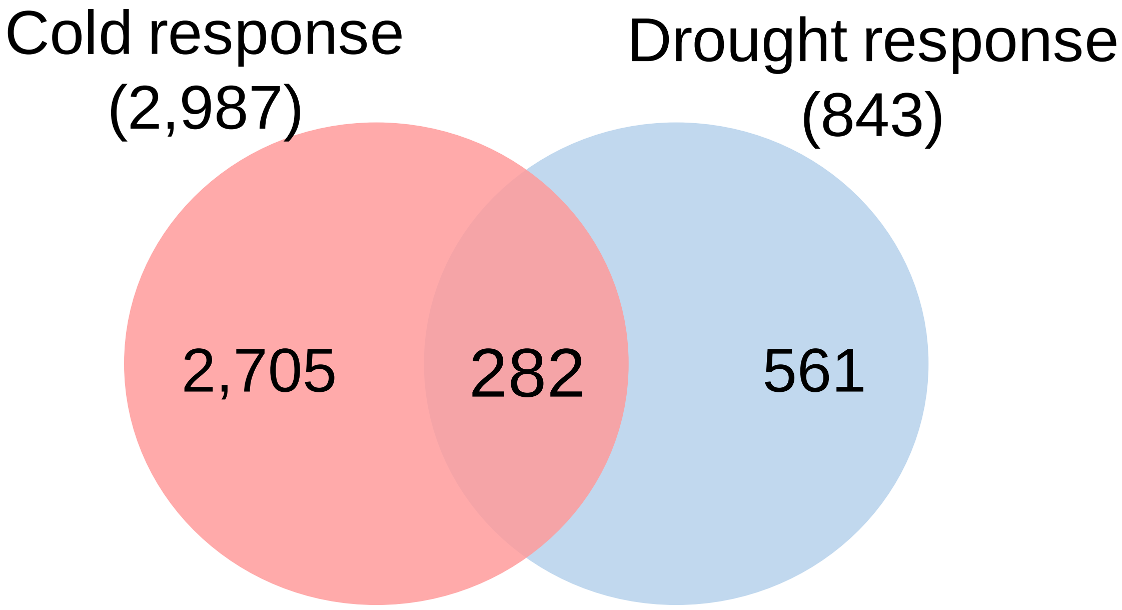

Figure S17: Venn-diagram represents differentially expressed coding genes under cold and/or drought stress of cassava.

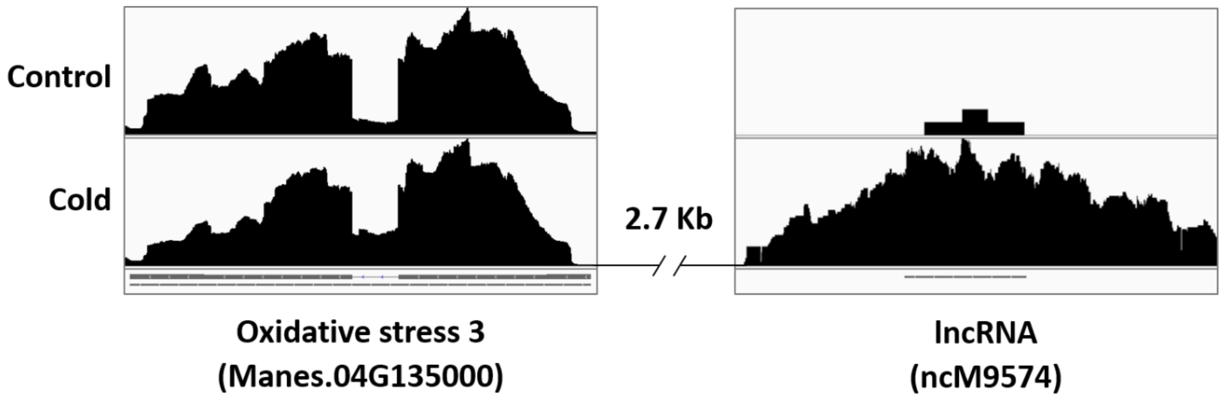

Figure S18: RNA-seq read alignment on ncM9574 and its target. Read alignment of lncRNA ncM9574 and its predicted *cis*-regulatory target gene, Manes.04G135000 in control and cold condition from RNA-seq dataset of Li et al [15]. The black peak represents read coverage and abundance.

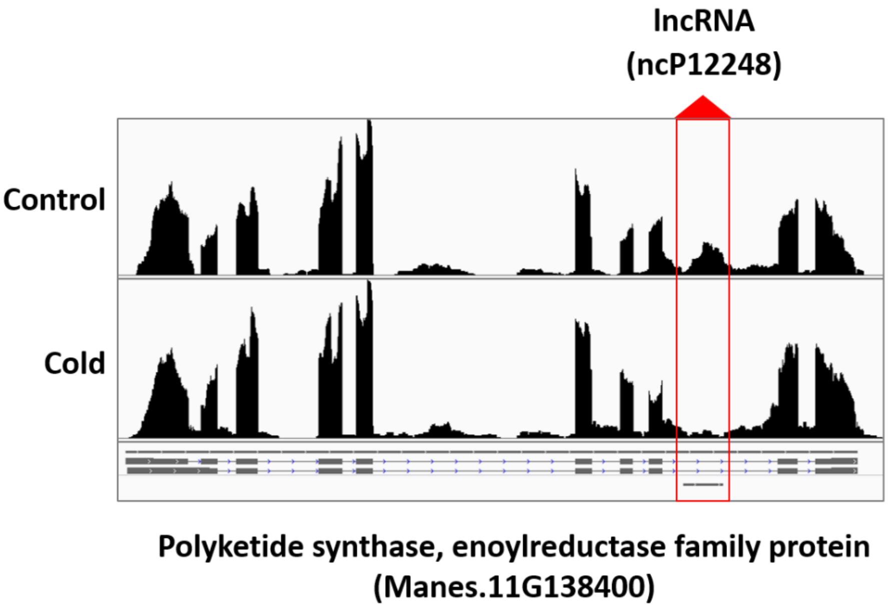

Figure S19: RNA-seq read alignment on ncP12248 and its target. Read alignment of lncRNA ncP12248 and its predicted *cis*-regulatory target gene, Manes.11G138400 in control and cold condition from RNA-seq dataset of Li et al [15]. The black peak represents read coverage and abundance.

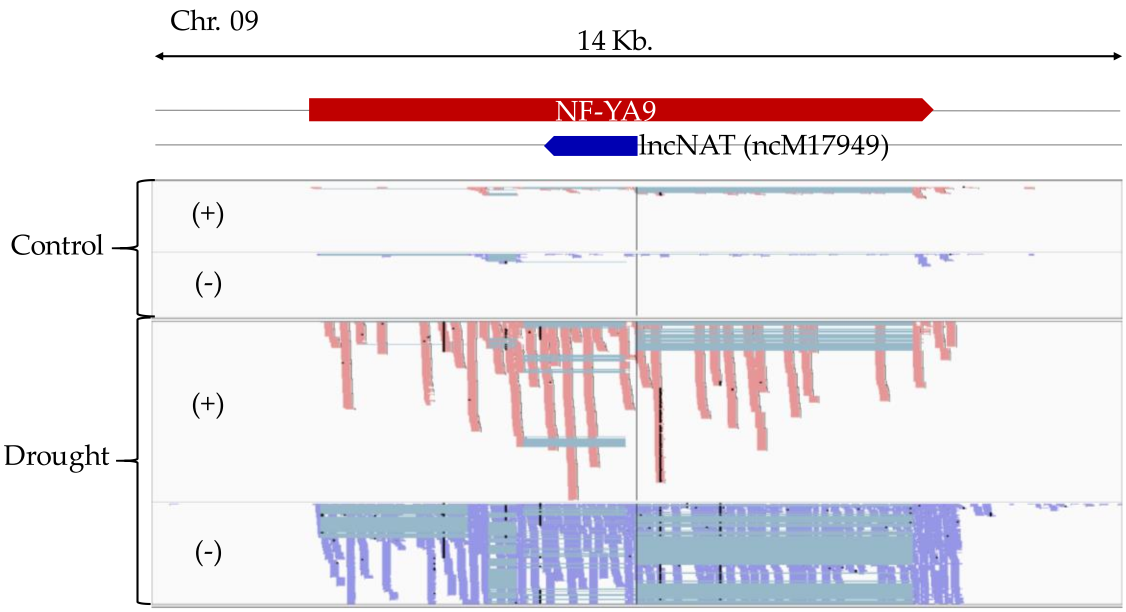

Figure S20: RNA-seq read alignment on ncM17949 and its target. Read alignment with strand specific of lncNAT ncM17949 and its predicted *cis*-regulatory target gene, Manes.09G025200 in control and drought condition from RNA-seq dataset of Li et al [15]. The pink aligned reads represent read coverage and abundance in plus strand and the blue aligned reads present read coverage and abundance in minus strand.

```
5'
GCTGTAAAGCAATGTCATATAACTTGACAAGTGGCAACAAATTCCATTCCCGACGTGCAAGGTCCATGGGTAAGTATGGCTGTCCATATGATGATTTATGGATCAGAAGATGGGAACGATAC
ACCTTGCAAGGGAAAATTTTCCACGTACAACTCTGGATCTCTCCCCATAAATAAGCATTAAAAACTAACAACAACTAATGGTTCATGCCAAAGATCATTGTACATGTGTACGAAAAAGTTAA
GGGAGAAATATGAACGAGTTTACACGCAACTCCAAAATAGAGTGAGAAAGAGAGTTTACAAGTGAAGGGCCAACATGTTCCAGTTGAGTAGGTGGTACAAGATACTCCACCATCGGTGGAGG
CATGAAGGATACAGAATGTTGTAGATGTTGTTGTTGATGTTCTTCACCATATTTTCCATCTGTCAACAGAAACAGTATAATTGTTTTGTATTCACATTTTTTTGGGCTAAAATGCATTAGGT
TACAAGCATAATTGTATTGCATAACATTTT 3'
..|||.||..||...............|||.............||..........||..................|||.........................................
||......|||.||...||||.......||...........|||............................||.......................................||..
.........................................||.||..|||....||||||||||||||||||||||||||||||||||||||||||||||||||||||||||||||||||||||||
|||||||||||||||||||||||||||||||||||||||||||||||||||||||||||||........||..................||..............................
....|||........|||........|||.
3'
GATGGTATTAAGAAAGTACAACGATAGGGAAAGAAGATGGTAGGATAGACTAACACTCACTGTACTACTGTTAACTTTAGGTTCTTTTATAACCCTCGTGCGTCATGGTAGCGGTGGTAACA
TCCAGGCAACACTCTAAACAAATACATTTACCAGTCCAAGATAATGTTGTAATGGACGTCTACTTAGCCTTGCGAATCTTCTTACACGACTACTACTCAAACATCTTCCAAAATCTTGTCAC
AGTTATTTCAACAAGCGGAATAGGTTGCACCAAAATCGTCGTGACAATAGTTCGAAGAATCACAAATATTTCGCCGGAGGTGTTGGAGAACGGGAAGAGTACCGTACATTTACAGCTCTGAG
TACGTCCATTCCGAAAGATTGAAATTAGTCAAAGAAAAGGTCGAGTCGAAATCGTGCGCTAACAGAAGCGGAATCTTATGGTACTATGACGAACCGTAAATGTATGTGTCCAAGAAGACAGT
AAAGTTCCCCGTTGTGGTAAGATCACCCCATCCGAGGTTCTATTGTTCTTACCTGTTCACTTCCCGGTTGTACAAGGTCAACTCATCCACCATGTTCTATGAGGTGGTAGCCACCTCCGTAC
TTCCTATGTCTTACAACATCTACAACAACAACTACAAGAAGTGGTATAAAAGGTAGACTAACACCGTTACGTCACTTGACGTAAAGAAATATTCGTAGTCAAGGGAGCGGTTTAACTACTAA
TCGACGAGCACTAAACGGTCTAAAAGGGGGTGTTGGTAGTAGGTACATTAAACTACAGTTGTTTAAAAAGCATGAAAGGAGATTGTACAGACTATTTTGTTGTAACGAGGATTGTGGTACGG
TGGTTCGAAATCTGCTTCGGTATGTTAATCACCCTAGATTAACACCTACCGATCACAATAGGTTAAAGTAAACGTA 5'
```

Figure S21: Direct binding of ncM17949 and its target. Sequence and position of direct binding prediction between lncNAT (ncM17949) and mRNA of target gene Manes.09G025200 encoding for nuclear factor Y, subunit A9 using LncTar with normalized free energy (ndG) < -0.1, which reflects the relative stability of internal base pairs. The top is lncNAT (ncM17949) sequence in 5' to 3'. The middle indicates the complementary of binding region on position 1-518 of lncNAT (ncM17949) and on position 241-758 of mRNA target. The bottom is Manes.09G025200.1 mRNA sequence in 3' to 5'.

```
5'
GGGAACATTAATCAGTTCATAGGCCCCACACGGACTGGAACTACCGATAGGGAGAGTCGGTCTTGGTCCTTCCAACAAACCACGAACTGGACCAGAACTGATAATTCGGTCCAATCTAACCT
ACCAATTTAACAAAGAAAAAAGAATTTACATGCGCCACCTGGCATCTAGTAGGCGTGCAATGATCACAACTATTTCAATTTCCTTTTGAAATATTTACACTTCATAACTTTGCCATTT 3'

|..........|||||||.....................||.||......||....|||...........|||||.......||.......................||...........
.........................................................
3'
ATGGCTTCTTCTTCTTCTTCTTCCTCTTCTTCTTCTGGCTTAAATACTTATATAAATTCTCAACCCACCTTCTCTTTCTCGACCCATCTTCTCATGTCGTCGTCTTCTTCTTCTTCTTTCAC
CAATCTTCTTTCGACTAATATCAAAGACATGGACAGTATTAGCTGGGGACTTTATGACCATGGAACTGATGCAATTGGTATTGAAATCCCAAAGTTCAAATCATTTACACCTTCTTCTTTAC
CTCTTTCTCCTCCTCCTGTTTCCCCTTCTACTTATTTGGCCATTCCTCCTGATTTAAGCCCAGCTGAGCTCTTGGACTCGCCTGTTCTTTTTTCCACCTCTAATGTTTTTCCTTCTCCAATC
ACTGGGGCTTTTGCTGGTCAAAGTTTCAACTGGAGGAATAATTTTAGTGAGAATCAGAGAGGTGTTAAAGGGGAAGAGAGAAGTTTCTCTGATTTCTCTTTTCAAACCCAAACAGCTCCCAC
ATCTTCATCCTTCTTTCAATCTTCTTCAAGCTTTATCCCAGTGGAGCAGTCAATGAAAAAGCAACAGGAATCATGGAATTTTAACAAACCCACAAAGCAAACAGATTTTTCATCAGACAAGG
GAGGAGTTGTAAAATCAGAATGTAAACCAATGCAGTATTTCTCCTCAGAAATGGCTCCAATCCAATCAACCATACAAAATAACACTGCTTCTCAACTCAGTTACAATCATTACAACAATCAA
CCAGCTCCATACATGAGAGAGCAAAGAAAGTCAGATGATGGATACAACTGGAGGAAGTACGGACAGAAACAGGTTAAAGGAAGTGAAAATCCACGTAGTTATTACAAGTGCACTTATCCAAA
TTGCCCAACCAAGAAAAAGGTAGAGAGATCTTTGGATGGACAGATTACTGAAATAGTCTATAAAGGAAGCCACAATCATCCCAAGCCTCAGTCTACAAGATCATCCTCTCAGTCTATGCAAC
CTTCTGCAGGAGTCGGCTTAGAAATCTCAGATCAATCTGTTGCTCCATTGGAGTCTGTCAACATGCAGGAGGATTCTTCAATTTCGATGGGAGATGACGAATTCGAACAAAGCTCACCAATT
AGTAATTCAGGTGGAGATGACGATGAAAATGAACCTGAAGCCAAAAGATACAAGGGACAGAATGAAAATGAGGGCATTTTGGGTGCTGGGAGCAGGACTGTTAGAGAACCTAGGATTGTGGT
TCAGACAACAAGTGATATTGACATACTTGATGATGGGTATAGATGGAGGAAATATGGACAGAAAGTAGTCAAGGGAAATCCCAATCCAAGGAGCTACTACAAGTGTACGTCAGTTGGTTGTC
CCGTTCGAAAACATGTCGAACGAGCGTCGCACGACACAAGAGCAGTGATCACCACTTACGAAGGGAAGCACAACCATGATGTTCCAGCGGCTCGTGGAAGCGGCTATGCATCCAACAGACCT
CCATCCAATGGTAACCCCAGCAGCATGCCCATCCCAATAAGACCCTCAGTCACAACCAACACAAATTATCCAAGCTCGCTAAACAGCACAAGGTTACCAACATCAGCAAGTCAAGCTCCATT
CACCCTGCAGGAAATGTTGCAGGGTCCAGGGAGCATTGGGTTCTCAGGTTATGGGAAGCCAAGCGGCTCATACATGAACCAAACACAGAGTGCATACTCCGGAGCCAAGGAAGAACCAAAGG
ATGACTCATTTTTCGACTCTTTCCTCTGTTGA 5'
```

Figure S22: Direct binding of ncP456 and its target. Sequence and position of direct binding prediction between lncRNA (ncP456) and mRNA of target gene Manes.09G112700 encoding for WRKY DNA-binding protein 33 using LncTar with normalized free energy (ndG) < -0.1, which reflects the relative stability of internal base pairs. The top is lncRNA (ncP456) sequence in 5' to 3'. The middle indicates the complementary of binding region on position 1-179 of lncRNA (ncP456) and on position 1562-1740 of mRNA target. The bottom is Manes.09G112700.1 mRNA sequence in 3' to 5'.

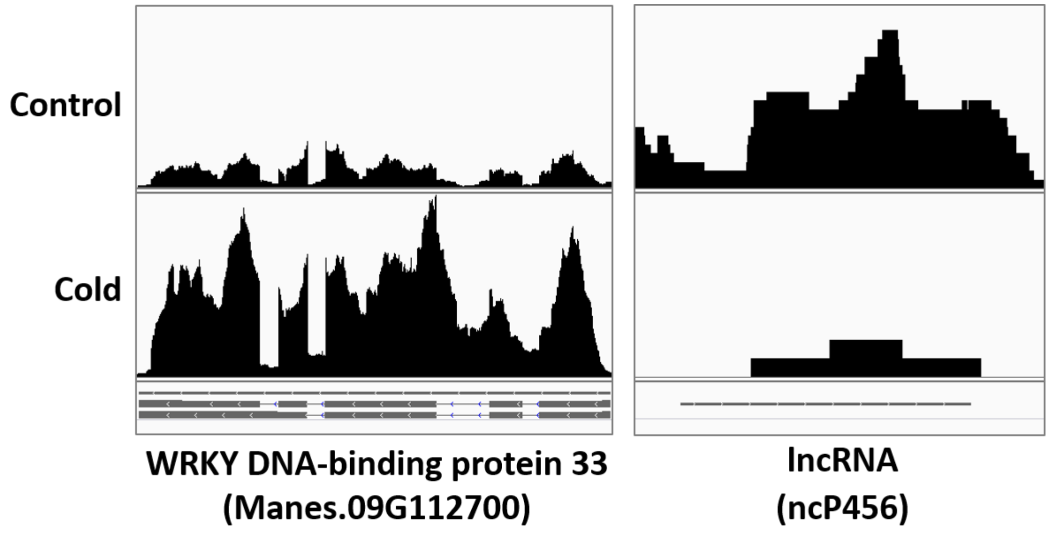

Figure S23: RNA-seq read alignment on ncP456 and its target. Read alignment of lncRNA ncP456 and its predicted *trans*-regulatory target gene, Manes.09G112700 in control and cold condition from RNA-seq dataset of Li et al [15]. The black peak represents read coverage and abundance.

```
5'
GAAACACTAAGTGTTATAATCAGTTCAGTGTAACTTGCTGAAGGGCTAGACCCAAAAAAGTTTAGGAACATGGTTCAGCCATGATTATTGTGCACTGATGGGTCAAACATTCATTATGGGTT
TCTTGTTTCCTTTATACTCTGTAACGGCTGACCTATTGGGCGTTTGCTACATATTTTGCATTAACAAGATCTATCCATTGTGACTTTTTATTGTTTACATTTTTCGTT 3'

...............................................||......|||..||.|||...............|||...||..........|||||............|||||....
...||......................||.........|||....||||.....||.......................................................
3'
AATAAATGATGGTAGACTCGATCCTCCCAAATATCTTCGAAGATACAGACTCCAACGTATGAAGTTCATTAAAAGATAGAGAAATAAGAGGTTCGAAGGTTCAGAAGCTACCTTGGTGAAGA
ACAATACTACTACTACTACTACTACTACAAAACAAGAGGAGAGCGATTACCGTTACCGATACAGTAACCCCTTCTCCAGAGCCTCGTGTTTCCGTACGTCATAACACCAATGTTCGTCTCGA
CACTGATACTTACACCTTTACTGTGTCTCATATTGTCGTTCTCAAACTCAATGATACAACCACTGAAGTAAACTCATGAATTATCAACGTTACCGACGAGGTCAGTAACCCTTTCACATTCG
GGTGGTTCGGTTACTCTTGAATTTAGGAGCCTTTTTTAATTATGCTTGACGGTCACTCTTCATTTCTTTGTGCCGTTACTTCATTCGTTAGGCGCTCGGTATTAGTTTCCTCGGAACTTAAA
AACGGGTTCGGTACCTTCGTTGTGAGCCTCGACGTTGTTTGTCTTTCTTATGACCTACCTCGAGGAAACCTTCTCAAAGTAACCAACCTTCAGAGACCATCTCCCAATGTTTATCCTGACAC
ATCACTCGGTTGGGTTGTCGTTTCTTCTTTTATCCAGGAAGAAAATCGGGGTAACTACGTGGGTTGTTACGCGGCTGTTTTAAGGGATGTTGACTGTCTACTAAGCTTCCTAAACGATGAGA
ACTTTCGGAGGAAACAGGAGGTCTGTAGGTAACAGGTATCTAGAATTCGAGTTCTGTCTCCTAACCTCAGTACTCTTACATGGTTTCCTGTGACAAGTCCAACAACCGTTCACTTCCTCCTT
GAGGTTCACGATTCTTGTCTTCCCGATAGGTACCTCGTTTCTTCTTTAACTGTGCCTAACCTACCATCATAAGTGCTGCTTGACGAAGTTTCATCTCTTAATTAAACTCTTCTATCTGCCTT
CGATGTTACCGATGTTCTCGTTCTCTCTACGTGGTATCATGTTCTAAATGTAAGACCGATTACACCTCTTTAAAATACCTACGTCTCCACCGGTCTCAAAAGGTGTACTAACGAACCGACGA
TTACGGTTCTGTTTATGGTTTACTACTCTATCCTATTGCTTCTTCTTTGCCGGTAAAGAACAGTAGACCCTCGTGATAAAGAAGACTCCGAGATTCCAAGAGGTTGAGTCCGGGAAGGTTAC
GTAGTATCATAGCTAGGTGTCCATTTCGTAGGAATAGTTGAGATACGAGGTGTCTTAAGAGACCACTTCCAAGAACACCGAAGGGAGCATTCACGCTATAGACGGAGAGTTCAAAGGGTCTG
GTACAAAACTTCCTTAGTATAGAAAATAGGAAGTTAAAAACTAACACTACTCTCAAACATTGTAACTGCGGGTTGAGTTGACGTGTAGGGAAACCGTAACCGACTCTTACCTTGAAATAAAC
AAACAAAACGCTGGACCGTCGGTATCTAAGTCGTCTTCTTTGGCTTTAGGGTAACGGCTTTGAACAAAGAAGTAATAGAAGTTTTGCTCGAAAAAGTAACTAGAGACTCGACGAAGGGGAAC
CTCTTCAACCGTACGACTACCTTTGTCTTGTTCAGGAATCTAGCAACTACCCAACTTTAAACTGTCTCGACAAGAGTCTTCGTTATCAAAGTTCAGGGTGTCGACTACTTCACTATCCTACT
CTTAGTGACGGTGAACCAACCGACACTACTCATAATTTCGATGACGACGATAGTTACGATAGCTTCGGCGAGTGAAGAAAACTACTATACATAAACTACTCACAACCTTACCTAAGAAGCAC
GAAACGATTAAGATCCTAAAGGTA 5'
```

Figure S24: Direct binding of ncP12197 and its target. Sequence and position of direct binding prediction between lncRNA (ncP12197) and mRNA of target gene Manes.06G154600 encoding for SLAC1 using LncTar with normalized free energy (ndG) < -0.1, which reflects the relative stability of internal base pairs. The top is lncRNA (ncP12197) sequence in 5' to 3'. The middle indicates the complementary of binding region on position 1-230 of lncRNA (ncP12197) and on position 12-241 of mRNA target. The bottom is Manes.06G154600.1 mRNA sequence in 3' to 5'.

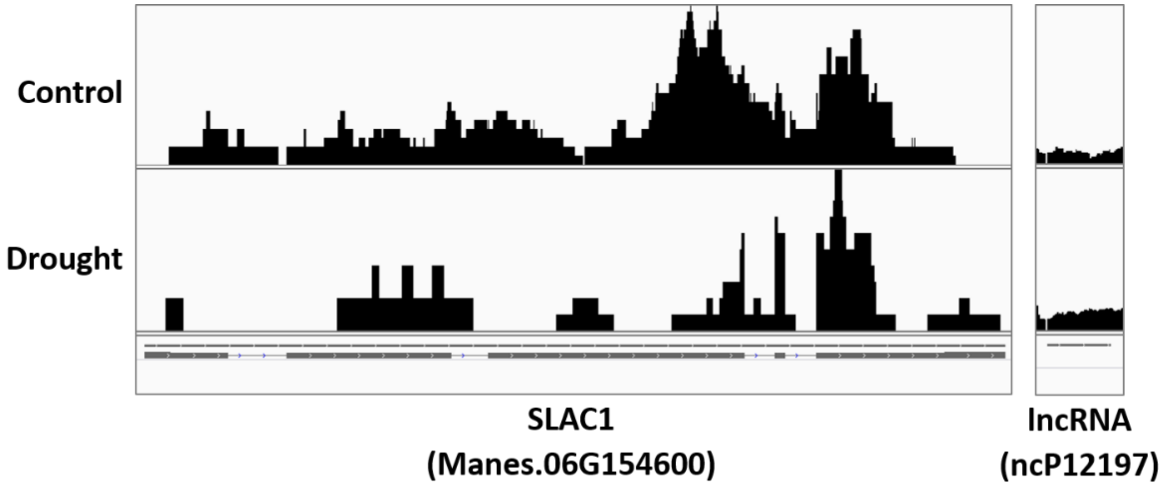

Figure S25: RNA-seq read alignment on ncP12197 and its target. Read alignment of lncRNA ncP12197 and its predicted *trans*-regulatory target gene, Manes.06G154600 in control and drought condition from RNA-seq dataset of Li et al [15]. The black peak represents read coverage and abundance.

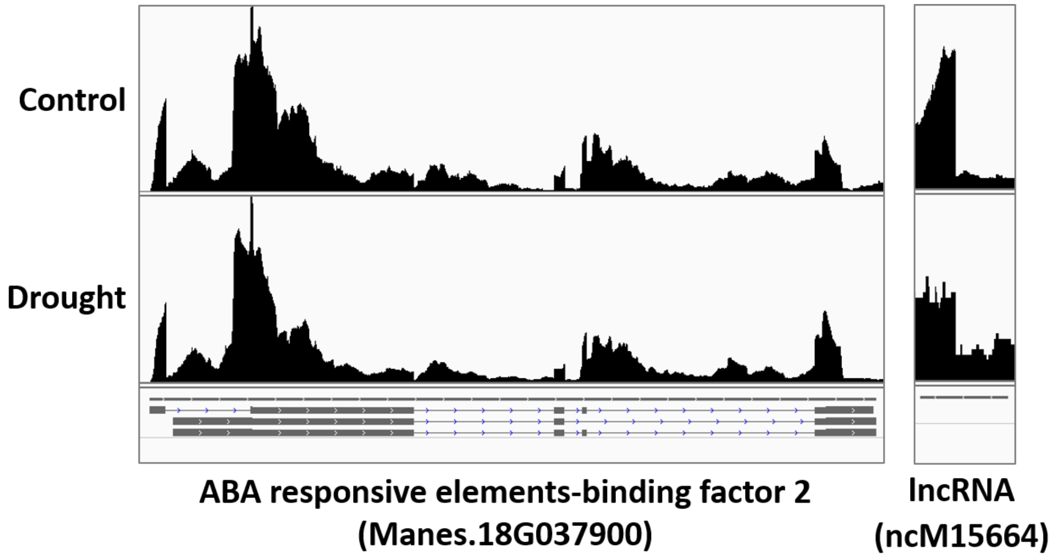

Figure S26: RNA-seq read alignment on ncM15664 and its target. Read alignment of lncRNA ncM15664 and its predicted *trans*-regulatory target gene, Manes.18G037900 in control and drought condition from RNA-seq dataset of Li et al [15]. The black peak represents read coverage and abundance.

```
5'
AAATTTTCTAATAAGTTGTCCAAACAAGGAACGGCAAGAGCGACATTATTTTTACCCTAGTGAAGTTTTAAGGATGGATGTAATGCTGCTCGCTCAGGGGATAAAAATTCAATTAAATGTTG
GAAAAAGGATTTGCTTAACAAACTAAAATAATCCAACTCCTTACCTTCTGCATTTCAGACTCAATAGAATGTGGCAACAATCTGAAAGCAGCCAAGTACCACCTCCAAGCCTCCACGGCTTC
TGTAGTCACAACTCCTGAGGTACCAACTGAAGAAGG 3'

..............................................................................................................................
.................................||..|||...|||..||......|||...||.|||.....................||...|||..................|||......
||||....||............|||.||||||||..|
3'
AATGGTACCTGGCCAGACTCAGGAGGAGTTCGTTGCAAAGAAATGAGGGACGACGACTAAGTAGTAGAGGTAGTGGACTAAAAAGACGTAAAGGTAGTAACGGCGGACAAAGAAATCGTTAA
GAACCAAGAGGAGAAAATTGAAACGTTGAAGACGAAGGTTGAGGTATCATATTCGGACGAACGCTCGAGCACTGGAACGTCGTCTGAGAGACAAGAAATAGTAGGAAGAGACGGAGGAGAGT
TGGTGGAAGAGGTGTCGTGGTGATCGAGAGGAAGGGGAATCTGGAGGTAATTTCTGTATTCCTTGACCGCTGTGACTTCTACATAGGGGTGAAAATCATTAGGGTAGACTTCTGTCGACCAA
ACGTCCACTAGGACAATGTCGTGGAGGATCGGGTTGGTAAGGGTTTCGAGGTGGGACGTAGTTTGGTGGCGACTAAGGGACTAGGGGTTAGGGGTACTAAGGTGGGGAGTAAGGTCCTGATG
AAACGTTTCCGTAGACACAACGCATAGGGTTTAATCCGACGAATCCTTTATAAACGACAACGACGACGTGGACCCGGACTAAACTAGACTGGGGTAATTGTAAGTTTCCGTTTAAGCTCCTT
ACGTATCTTTAGACTAATCATAATAATAACAAGTCAGGGTCCGAGGATAAGACGACTTTGGGGTAAGGATTTCGTCTTATTCAGGGGCAGAAGCTATTTAGCCGTTCTTTTGGTGGTAATCG
TAATTGGAAACGTCGATTAACTTGTAGAAGAGAGTGGTGGGGTCGAGATTGGTTTTTGAGGAGATTTCAGTAGAGGGGATTTCAGACGACGGAGACCCCGTTTAATCGACGACGTCATGGTG
GAGGTAAGGGTATTAGGAAGCTATACAGAAAGGTGTGGAGTAGGTGTCAGAAAACCGACTCCCAAGCGCCGTCCCAGTTTCTCGGAACCGCGACGTCTAAGAGAACCCGTCGTGGTCTTCTC
CGATGGTACGAGACACAAAGGAGGCGTGAGGTATACAAGAACTCTTCGAGTAGGTACAAGTAACTGGGTTTCAGGAATGGATAAGGGGGGTACCACAAGACTTTAAGTAGCTTCCAGTTGCT
CATCTATCTACAGACAGACCGGTTCCCTTTCAAGGGGCCGCCCGCCCACCGCGACGGACGAGGAGGTGGCCGCAGTCGGCTTCCCAGCAACCACTTCAAGAACTTCAACTATAATCAGGGGT
A 5'
```

Figure S27: Direct binding of ncM15664 and its target. Sequence and position of direct binding prediction between lncRNA (ncM15664) and mRNA of target gene Manes.18G037900 encoding for ABA responsive elements-binding factor 2 using LncTar with normalized free energy (ndG) < -0.1, which reflects the relative stability of internal base pairs. The top is lncRNA (ncM15664) sequence in 5' to 3'. The middle indicates the complementary of binding region on position 1-280 of lncRNA (ncM15664) and on position 81-360 of mRNA target. The bottom is Manes.18G037900.2 mRNA sequence in 3' to 5'.

```
5'
CCACTAGGCCAAGTCACAAGCGAGTTTCGCTAGATCATCTGAGTGTTGGACCCACTATACAAGGCCACAAAGGTGGGTTCCGCTAGGCCAGATCACAAAGCGGATTTCATCAGACCATCCTG
GCATTGGTCCCACTATATAAGGCCACACGGCAGGTTATGTCAGATCAGGTCACAAGGTGGGTTCCACCAAACCATCCGGGTGTTGGACCCACTATACAAGGTGGGTTCCGCCAAGCCAGGTC
ACAAAGCAGGTTTTGCCAGACCATTCGGGCGTTGGTTCCACTATACAATGCCACAAGGCGGGTTACGCCAGGTCAG 3'

................................................................||.....|||..............................||...................
.||............................|||..............||........................||||||............|||............................||
|..||............|||......|||.|||||.........|||.............|||.......||||||.
3'
ATGTGCAGTGGATCAAAGAAGAAACCAACTCATACAGGTGTCTTCATGGGGAATGAATTTCAAAAAGATGGGTATTGCAGTGATCTCTCTGTTTTGCTTGAATTGTCTGCCTCGAATGATCT
GATTGGATTTAAGAGAGAAATCCAAGGAGGCCGTGAGGTTGATGAGCCTGGCTTATGGTATGGGGGAAGAATTGGCTCAAAAAAGATGGGGTTTGAGGAGAGGACTCCCCTCATGATTGCTG
CCTTATTTGGAAGCAAGGATGTCTTGAGTTATATCTTGGAAATGGGCCATGTTGATGTTAATCGGTGTTGTGGGTCTGATGGCGCCACAGCCCTTCACTGCGCTGTTGCAGGTGGTTCTGCA
TCTTCTGTTGAGGTTGTCAAGCTCTTGCTTGCTGCCTCTGCTGATCCTAACGCTGTTGATGCCAATGGAAATTGTGCTAGTGATTTGCTTGTTCCAGTTGCTTGTTTCAGTTTTAATTTGAG
GAGACAGGCACTAGAGCTTGTGCTAAAGGGTGGCTGTACTAGTGATGAATCTTGTGTTTTGGCTGATCAGAATCCTGATGAAATAGATGTGCAACAGCAGCAGGAAGTTTCAACTCCAAGAT
TATTTAAAGATGGGACTGACAAGAAAGAATATCCTGTCGATCTCACTCTTCCTGATATCAATTATGGTATATATGGTACTGATGAATTTAGAATGTATACATTTAAGGTGAAGCCTTGCTCA
AGGGCATATTCCCATGATTGGACAGAATGCCCATTTGTTCATCCTGGTGAGAATGCAAGGCGGCGAGATCCAAGGAAATACCATTATAGTTGTGTCCCATGCCCTGAGTTCCGGAAGGGTTC
ATGCAGGCAAGGTGATGCTTGTGAATATGCACATGGTATTTTTGAGTGCTGGCTTCACCCTGCCCAATATCGAACACGTCTCTGCAAAGATGAGACTAATTGCTCAAGAAGGGTTTGTTTCT
TTGCTCACAAGCCTGAAGAGCTTCGACCCTTGTATGCCTCAACAGGTTCAGCTGTGCCTTCACCAAGATCTCTCTCAGCCAACGGTTCAGCACTAGACATGGGTTCTATCAGTCCACTTGCC
CTCGGTTCTCCATCTGTCATGATACCACCTACATCAACCCCACCCGTGACTCCTACTGGGTCCTTATCTCCTCTGGGTAGTTGGCCCAGTCAGTCTAATATTGTGCCCCTTAACTTGCAGCT
TCCTGGTAGTAGGCTGAAATCTGCACTGAGTGCTAGAGATATGGATATGGATGCAGAGTTGTTTGCGCTTGATAGTCACCATTGCCGGCAACAACTGATGGATGAGATTTCTGGTCTCCCCT
CACCTTCCAGTTGGAACAATGGTTTGTCCACTGCTTCAGCTTTTGCTATCTTTGGTGATCGAACTACGGAGTTGACTAGGCTTGGAGGAGTGAAGCCAACTAATCTTGAGGGTATTTTTGGA
TCTCTTGATCCTACAATTTTGCCTCAATTGCAAGGACTCCCAGGGGATGCCACTGCATCGCAATTACAGTCTCCAACAGAGATTCGGATGCGCCAGAACATTAACCAGCTGCTTCGCTCAAG
CTACCCTACCAACTTCCCATCTTCTCCTGTGAGGACACCATCTTTCAGGATTGACTCATCTGGTGCAGCAGCAGCAGCAGTTTTGAATTCAAGGGCTGCCTTTGCAAATCGGAGCCAGAGTT
TCATTGAGAGAAGTGCTGTGAACCGTCATACTGGGTTTTCTTCACCAACTTCTTCAGCAACTATACTGCCTTCTAATCTTTCAGACTGGGGTTCACCTGATGGCAAATTAGAATGGGGCATG
CAAGGACAAGGGCTTGATAAACTGCGAAAGTCTGCTTCCTTTGGCATTCGAAGCAATGGCAGCAGTTTGGCAGTAGCTGCAGTCTCAGAGCCTGCAACTGTTGACAAGCTAGATGTGTCATG
GGTTCAGTCCTTGGTAAAGGATACCCCTCCTCAGCATTCTGGGCACTTCAGCTTTGAGGAACAGCAACAGCAATGCCTTATTAACACTGGAGGTTCAGAAATGCTTCCAGCTTGGGTAGAGC
AATTATACATTGAGCAGGAGCAGATGGTGGCTTAA 5'
```

Figure S28: Direct binding of ncM12154 and its target. Sequence and position of direct binding prediction between lncRNA (ncM12154) and mRNA of target gene Manes.01G160800 encoding for zinc finger (CCCH-type) family protein using LncTar with normalized free energy (ndG) < -0.1, which reflects the relative stability of internal base pairs. The top is lncRNA (ncM12154) sequence in 5' to 3'. The middle indicates the complementary of binding region on position 1-320 of lncRNA (ncM12154) and on position 1035-1354 of mRNA target. The bottom is Manes.01G160800.1 mRNA sequence in 3' to 5'.

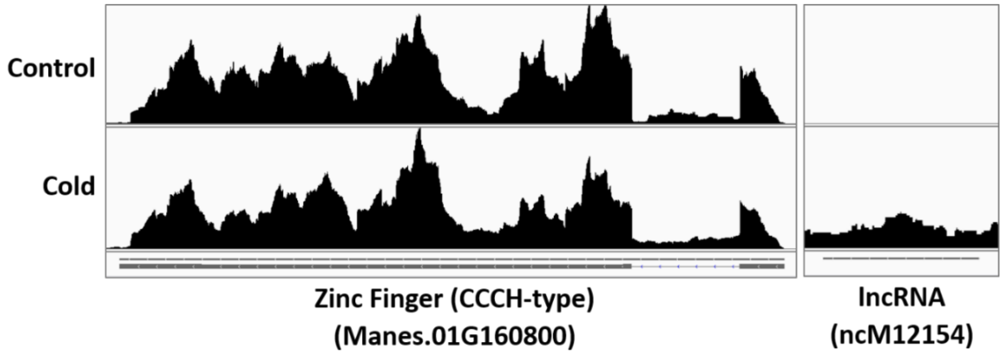

Figure S29: RNA-seq read alignment on ncM12154 and its target. Read alignment of lncRNA ncM12154 and its predicted *trans*-regulatory target gene, Manes.01G160800 in control and cold condition from RNA-seq dataset of Li et al [15]. The black peak represents read coverage and abundance.

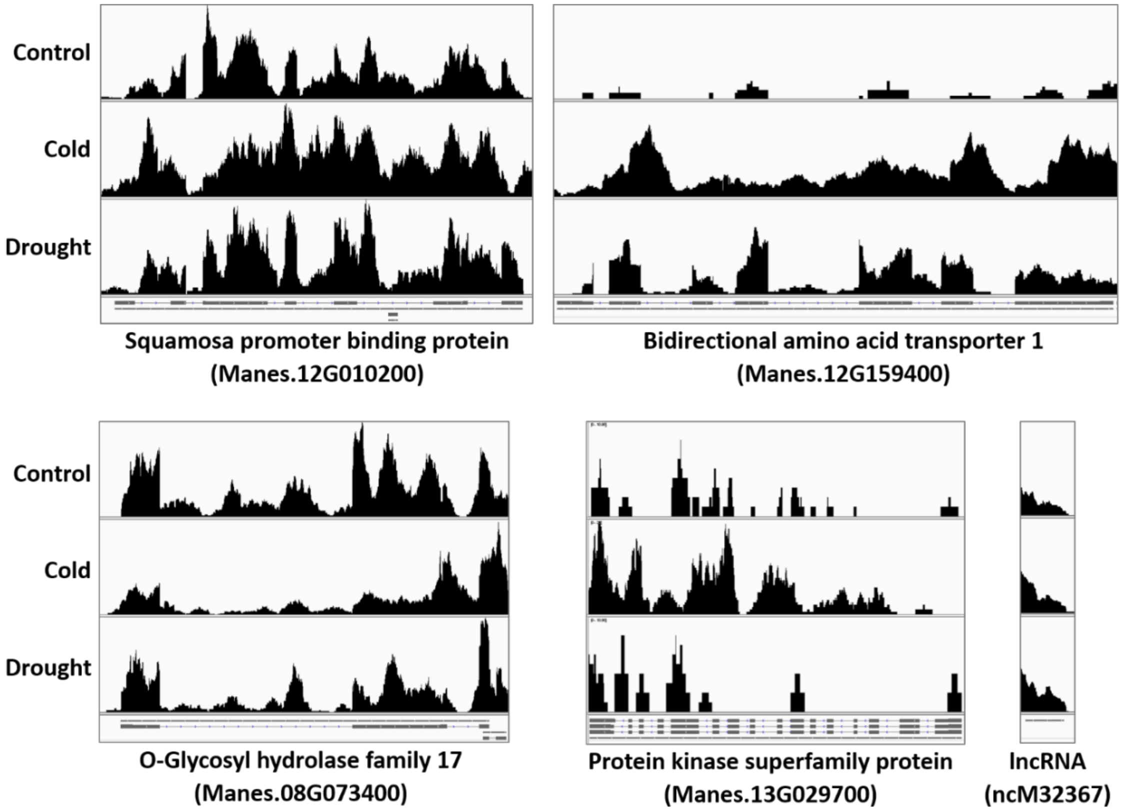

Figure S30: RNA-seq read alignment on ncM32367 and its target. Read alignment of lncRNA ncM32367 and its predicted *trans*-regulatory target gene, Manes.12G010200, Manes.12G159400, Manes.08G073400 and manes.13G029700 in control, cold and drought condition from RNA-seq dataset of Li et al [15]. The black peak represents read coverage and abundance.
